# Gut microbiota restricts intestinal lipid uptake via modulation of bile phosphatidylcholine metabolism in mice

**DOI:** 10.1038/s41564-026-02434-z

**Published:** 2026-07-29

**Authors:** Sarah Brunner, Johannes Plagge, Maria Zimmermann-Kogadeeva, Marcus Höring, Gerhard Liebisch, Marijana Basic, Silvia Bolsega, Klaus-Peter Janssen, Emma Slack, Sophia von Gamm, Alina Viehof-Beckmann, Thomas Clavel, Michael Zimmermann, Joerg Heeren, Piero Giansanti, Anna S. Weiss, Sven Hermeling, Aline Dupont, Anna-Lena Ullrich, Florian Jokisch, Claudine Seeliger, Andre Bleich, Maria Hidrobo, Bärbel Stecher, Olivia I. Coleman, Claudia Moresi, Giorgia Greter, Markus Arnoldini, Josef Scheiber, Silke Matysik, Martin Klingenspor, Bernhard Küster, Dirk Haller, Ralph Burkhardt, Folkert Kuipers, Josef Ecker

**Affiliations:** 1 Institute of Clinical Chemistry and Laboratory Medicine, Functional Lipidomics and Metabolism Research, University Hospital Regensburg, Regensburg, Germany; 2 ZIEL Institute for Food and Health, Research Group Lipid Metabolism, Technical University of Munich, Freising, Germany; 3 Genome Biology Unit, European Molecular Biology Laboratory, Heidelberg, Germany; 4 Institute for Laboratory Animal Science, Hannover Medical School, Hannover, Germany; 5 Department of Surgery, School of Medicine and Health, University Hospital rechts der Isar, Technical University of Munich, Munich, Germany; 6 Laboratory for Food Immunology, ETH Zürich, Zürich, Switzerland; 7 Functional Microbiome Research Group, Institute of Medical Microbiology, Rheinisch-Westfälische Technische Hochschule Aachen University Hospital, Aachen, Germany; 8 Molecular Systems Biology Unit, European Molecular Biology Laboratory, Heidelberg, Germany; 9 Department of Biochemistry and Molecular Cell Biology, University Medical Center Hamburg-Eppendorf, Hamburg, Germany; 10 Bavarian Center for Biomolecular Mass Spectrometry at the University Hospital rechts der Isar, Technical University of Munich, Munich, Germany; 11 Max von Pettenkofer Institute of Hygiene and Medical Microbiology, Faculty of Medicine, Munich, Germany; 12 Institute of Medical Microbiology, Rheinisch-Westfälische Technische Hochschule Aachen University Hospital, Aachen, Germany; 13 Partner site LMU Munich, German Center for Infection Research, Munich, Germany; 14 Chair of Intestinal Microbiome, School of Life Sciences, Technical University of Munich, Freising, Germany; 15 Department of Nutrition and Immunology, Technical University of Munich, Freising, Germany; 16 BioVariance GmbH, Waldsassen, Germany; 17 Chair of Molecular Nutritional Medicine, TUM School of Life Sciences, Technical University of Munich, Freising, Germany; 18 Chair of Proteomics and Bioanalytics, Technical University of Munich, Freising, Germany; 19 Bavarian Center for Biomolecular Mass Spectrometry, Technical University of Munich, Freising, Germany; 20 European Research Institute for the Biology of Ageing, University of Groningen, University Medical Center Groningen, Groningen, the Netherlands

**Keywords:** Lipids, Mass spectrometry, Microbiome

## Abstract

The gut microbiota influences host metabolism, but the mechanisms of lipid uptake from food remain mysterious. Here we used stable isotope-labelled tracers in gnotobiotic mouse models, which revealed that host uptake of dietary lipids depends on microbial colonization. Systemic lipid metabolism modelling predicted that the gut microbiota restricts intestinal lipid absorption, and labelled lipid administration verified that the gut contents of microbiota-colonized mice contained up to 12-fold more lipids than those of germ-free animals. A combination of lipidomics and proteomics showed that gut microbes trigger Myd88 signalling, leading to a downregulation of hepatic Cyp7b1 activity and increased taurocholate production. Taurocholate stimulates phospholipase A1 activity in bile, causing the degradation of phosphatidylcholine that is essential for luminal micelle formation and lipid uptake. A diverse microbiome was associated with lower phosphatidylcholine content. This previously unrecognized host–gut microbiota interplay via enzymes in bile could provide future targets to modulate dietary lipid absorption.

## Abstract (web summary)

Lipidomics and proteomics in mice colonized with variable gut microbiota reveal an important role of gut microbes in restricting lipid uptake via triggering Myd88 signalling and the degradation of phosphatidylcholine in bile.

## Main

Lipids are a critical energy source providing more than 30% of total caloric intake in adults^1^. Systemic uptake of dietary lipids, predominantly occurring as triglycerides (TG), is a multistep process^2^. It comprises their emulsification with biliary bile acids (BA) and phospholipids, hydrolysis to fatty acids (FA) by pancreatic TG lipase (PNLIP), before uptake into enterocytes. After re-esterification to complex lipids, they are packaged into chylomicrons that are secreted into the lymph entering the circulation. Although nutrient uptake and the body’s energy balance were associated with the gut microbiota^3^, microbial impact on systemic uptake of dietary lipids is not very well understood.

The gut microbiota is a complex and dynamic ecosystem of ~39 trillion cells with a mass of ~0.2 kg for a 70-kg adult, located in the gastrointestinal tract (GIT)^4^. It makes non-digestible nutrients available for the host and impacts host metabolism^5^. Acetate originating from gut microbial degradation of fibre is a precursor for hepatic synthesis of lipids distributed into the circulation^6^. Primary BA are synthesized in the liver, conjugated to taurine or glycine, secreted into bile and stored in the gallbladder. After release into the gut lumen, BA can be metabolized by the gut microbiota and reabsorbed into the circulation^7^. Evidence that the gut microbiota impacts dietary lipid uptake is available. The fermentation products lactate and acetate from *Lacticaseibacillus paracasei* and *Escherichia coli* impede chylomicron secretion from mouse enterocytes^8^, whereas *Clostridium bifermentans* promotes TG uptake in mice fed with a high-fat diet^9^.

We previously reported that levels of dietary-derived polyunsaturated FA including docosahexaenoic acid (FA22:6 *n-3*) in plasma were inversely correlated with the presence of the gut microbiota^6^. Therefore, we here investigated the gut microbiota’s fundamental role in intestinal lipid absorption using stable isotope-labelled lipids and mass spectrometry in germ-free (GF), specific pathogen-free (SPF) and gnotobiotic mice colonized with different microbial consortia.

## Results

### The gut microbiota reduces systemic uptake of dietary lipids

**Fig. 1:**
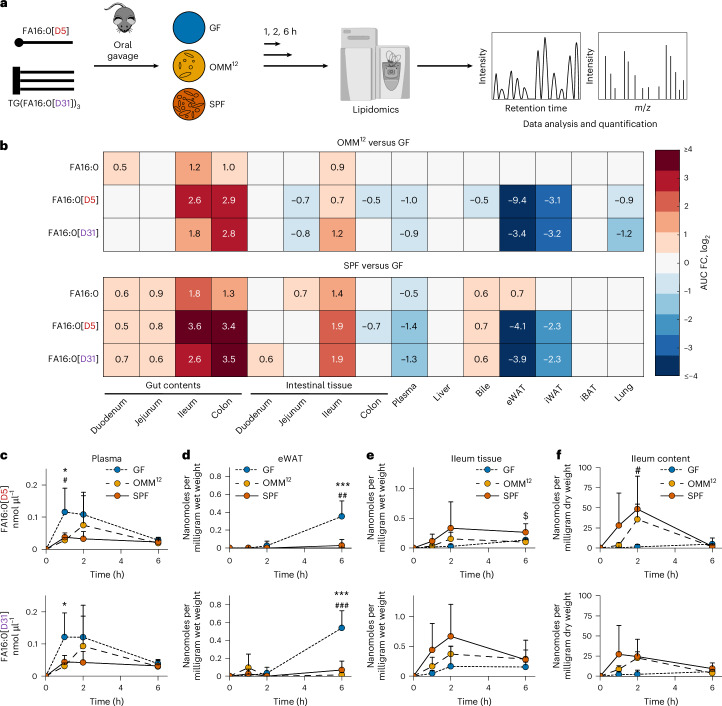
The gut microbiota reduces systemic lipid uptake. **a**, The experimental labelling strategy. **b**, A heat map depicting FCs (>0.5) of AUC from the 0-6 h time courses after tracer gavage for GF versus OMM^12^ and GF versus SPF mice. GF: *n* = 5 mice at 1 h and 2 h, *n* = 4 mice at 6 h; OMM^12^: *n* = 4 mice at 1 h, *n* = 5 at 2 h and 6 h; SPF: *n* = 5 mice per timepoint. **c**–**f**, FA16:0[D5] and FA16:0[D31] in plasma (**c**), eWAT (**d**), ileum tissue (**e**) and content (**f**). Means ± s.d. GF: *n* = 5 mice at 1 h and 2 h, *n* = 4 mice at 6 h; OMM^12^: *n* = 4 mice at 1 h, *n* = 5 at 2 h and 6 h; SPF: *n* = 5 mice per timepoint. *^,^^#^*P* < 0.05, **^,^^##^*P* < 0.01, ***^,^^###^*P* < 0.00, one-way ANOVA with Tukey post hoc test, OMM^12^(*) or SPF(^#^) compared with GF. Note, when comparing **b** with **c**–**f**, in **b**, the FCs of the AUCs for all timepoints (0–6 h) are presented, whereas in **c**–**f**, the time courses of the absolute concentrations at 0 h, 1 h, 2 h and 6 h are shown. Source data

To investigate whether the gut microbiota impacts dietary lipid uptake and flux, we applied stable isotope labelling in vivo. GF, Oligo-MM^12^ (OMM^12^) and SPF mice simultaneously received deuterium-labelled palmitic acid (FA16:0[D5]) and tripalmitin (TG(FA16:0[D31])_3_) per oral gavage (Fig. 1a). SPF mice harbour diverse gut microbiota^6^, OMM^12^ mice are colonized with 12 microbial strains representing the major murine gut microbiota phyla^10^. The body mass between GF, OMM^12^ and SPF mice on a standard chow diet is not different^11^. Total FA were quantified 1, 2 and 6 h after gavage by gas chromatography–mass spectrometry (GC-MS) in gut contents, plasma, bile and tissues. The flux kinetics are: in the first 20 min, the lipid mix reaches the half of the jejunum. After 1 h, tracers can be detected in colon tissue and they peak in blood plasma, before peripheral tissues including liver at 2 h and white adipose tissues at 6 h are reached. If the gut microbiota affects TG lipolysis in the gut lumen, different time-dependent concentrations profiles (1–6 h) of FA16:0[D5] and FA16:0[D31] will be expected in plasma.

**Extended Data Fig. 1:**
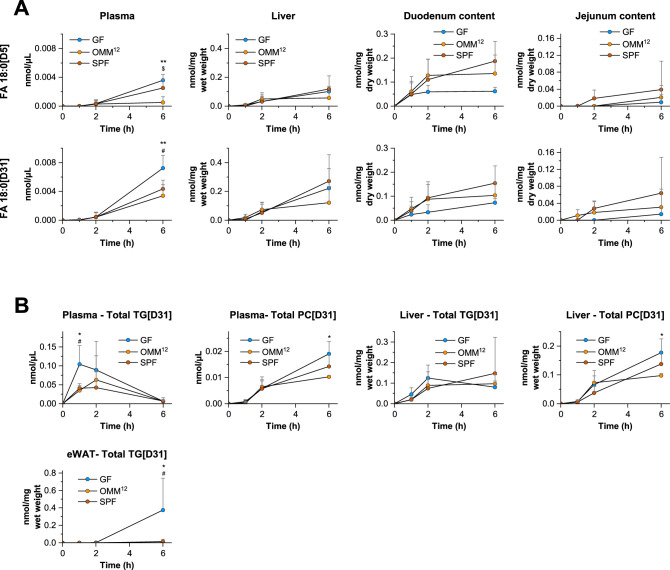
Metabolism of labelled FA 16:0 to FA 18:0 and complex lipids (GF, OMM^12^, SPF). (**a**) Levels of FA 18:0[D5] and FA 18:0[D31] in plasma, liver, duodenum content and jejunum content at 1, 2 and 6 h. Means +SD. GF: n = 5 mice at 1 h and 2 h, n = 4 mice at 6 h; OMM^12^: n = 4 mice at 1 h, n = 5 at 2 h and 6 h; SPF: n = 5 mice per time-point. */^#^/^$^p < 0.05, **/^##^/^$$^p < 0.01, one-way ANOVA with Tukey post-hoc test. (*) OMM^12^ vs. GF, (^#^) SPF vs. GF, (^$^) SPF vs. OMM^12^. (**b**) Levels of TG[D31] in plasma, liver and eWAT, and PC[D31] in plasma and liver at 1, 2, and 6 h. Means +SD. GF: n = 5 mice at 1 h and 2 h, n = 4 mice at 6 h; OMM^12^: n = 4 mice at 1 h, n = 5 at 2 h and 6 h; SPF: n = 5 mice per time-point. */^#^/^$^p < 0.05, one-way ANOVA with Tukey post-hoc test. (*) OMM^12^ vs. GF, (^#^) SPF vs. GF, (^$^) SPF vs. OMM^12^. Source data

Mice with gut microbiota had a reduced uptake of tracers into the circulation and peripheral tissues. In plasma and white adipose tissue, the areas under the curve (AUCs) for FA16:0[D5] and FA16:0[D31] were much smaller in SPF and OMM^12^ compared with GF mice (log_2_ fold changes (log_2_(FC)) between −0.9 and −9.4; Fig. 1b–d). In ileum and colon contents, the AUCs of labelled FA were much larger in colonized compared with GF animals (log_2_(FC) between 1.8 and 3.6; Fig. 1b,e,f), because less labelled FA reached the circulation in SPF and OMM^12^ compared with GF mice. The results were almost identical for FA16:0[D5] and FA16:0[D31] indicating that TG lipolysis is not affected by the gut microbiota. The elongation products FA18:0[D5/31] were detected in intestinal contents, plasma and liver (Extended Data Fig. 1a). FA were integrated in phosphatidylcholine (PC) and TG of plasma, liver and epididymal white adipose tissue (eWAT), with increased levels in GF mice (Extended Data Fig. 1b).

These data show that the gut microbiota substantially modulates luminal and systemic contents of labelled FA.

### The gut microbiota limits FA absorption from the gut lumen into intestinal tissue

**Fig. 2:**
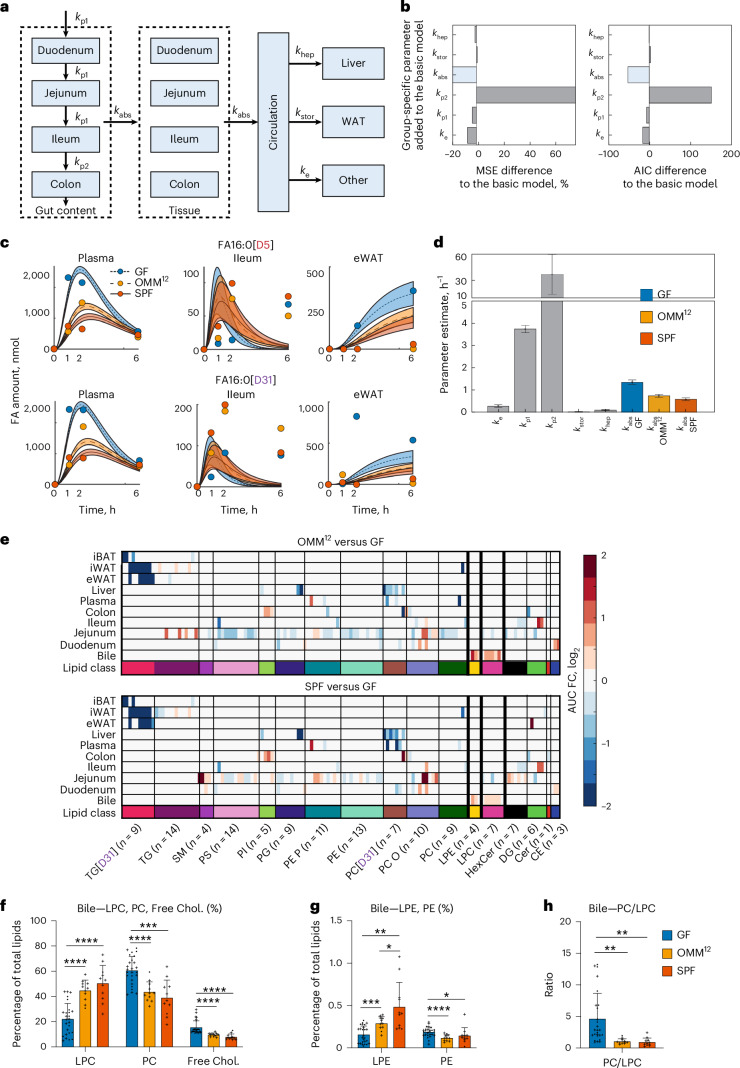
The gut microbiota limits intestinal lipid absorption and modifies phospholipid composition of bile. **a**, The FA flux model. Processes considered are indicated with coefficients *k*. **b**, A comparison of each of mouse group-specific models (with one specific parameter) to the basic model in terms of MSE reduction and AIC difference. For *k*_abs_, the group-specific model outperforms the basic model. **c**, Results of the best model (with group-specific *k*_abs_) fit to the labelled substrate distribution data in different tissues for GF, OMM^12^ and SPF simultaneously. Lines with shaded areas represent model fit and 50% confidence intervals for the model simulation results. **d**, The best model (with group-specific *k*_abs_ coefficient) parameters for general and mouse group-specific coefficients. Bars represent the best model (with group-specific *k*_abs_ coefficient) parameter estimates for general and mouse group-specific coefficients. Whiskers correspond to standard error of parameter estimates. **e**, A heat map depicting lipid species FCs after tracer gavage (0–6 h, AUC) for GF versus OMM^12^ or SPF. GF: *n* = 5 mice at 1 h and 2 h, *n* = 4 mice at 6 h; OMM^12^: *n* = 4 mice at 1 h, *n* = 5 at 2 h and 6 h; SPF: *n* = 5 mice per timepoint. Only FCs with *P* < 0.1 are shown after repeated ANOVA measures. Numbers of analysed lipid species are indicated in brackets. **f**,**g**, Relative abundance of LPC, PC, free cholesterol (Free Chol.) (**f**) and LPE, PE (**g**) of GF, OMM^12^ and SPF bile. **h**, PC/LPC ratio of GF, OMM^12^ and SPF bile. Means + s.d. GF: *n* = 26, OMM^12^: *n* = 11, SPF: *n* = 11. *P < 0.05, **P < 0.01, ***P < 0.001, ****P < 0.0001, two-sided *t*-test with unequal variance. + indicates female, • indicates male. Source data

**Extended Data Fig. 2:**
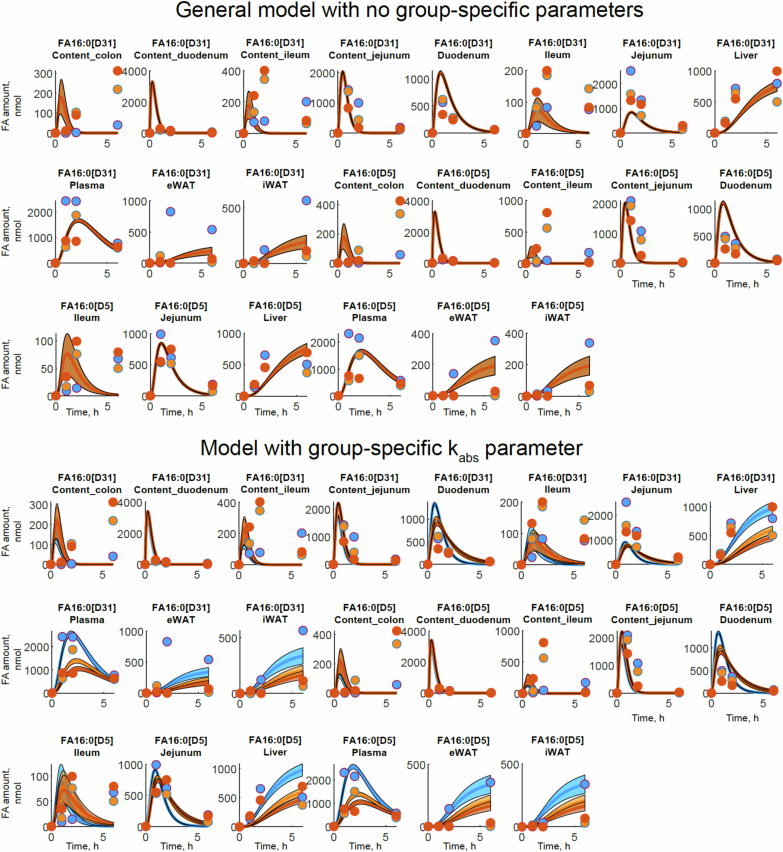
Model selection based on the group-specific intestinal absorption parameter. Comparison of the general model with no group-specific parameters to the model with a group-specific intestinal absorption coefficient parameter, which best explains the differences in FA 16:0[D5] and FA16:0[D31] kinetics in most tissues. Lines with shaded areas represent model fit and 50% confidence interval for the model simulation results. Source data

**Extended Data Fig. 3:**
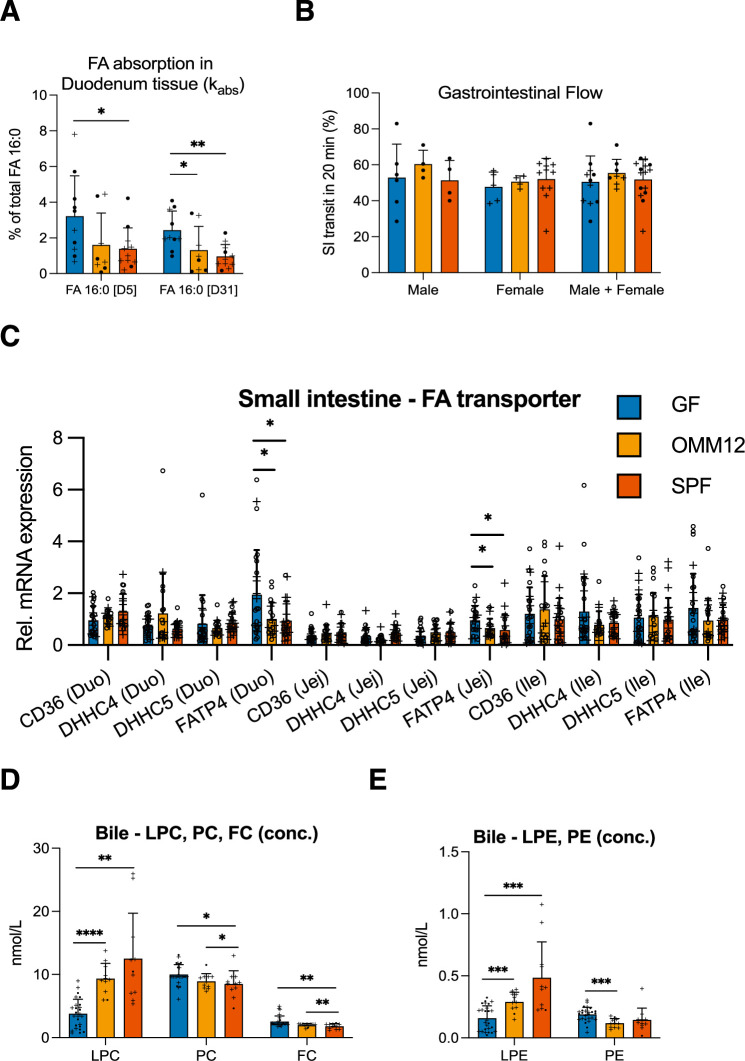
FA absorption, gastrointestinal flow and FA transporter expression in the small intestine, and lipid class composition of bile (GF, OMM^12^, SPF). (**a**) FA16:0[D5] and FA16:0[D31] in duodenum tissue of GF, OMM^12^ and SPF mice 20 min after gavage with tracers. Means +SD. GF: n = 10, OMM^12^: n = 8, SPF: n = 11. *p < 0.05, **p < 0.01, two-sided t-test with unequal variance. (**b**) Gastric flow in small intestine of GF, OMM^12^ and SPF mice. Shown is the distance that the Evans blue dye reached as percentage of the total small intestinal (SI) length at 20 min post-gavage. Means +SD. GF: n = 11, OMM^12^: n = 8, SPF: n = 15. (**c**) *Cd36*, *Dhhc5*, *Dhhc5* and *Fatp4* mRNA expression in duodenum (Duo), jejunum (Jej), and ileum (Ile) tissues of GF, OMM^12^ and SPF mice. Means +SD. GF: n = 26 (Duo), n = 23 (Jej), n = 28 (Ile); OMM^12^: n = 19 (Duo), n = 20 (Jej), n = 19 (Ile); SPF: n = 21 (Duo), n = 19 (Jej), n = 24 (Ile). *p < 0.05, two-sided t-test with unequal variance. (**d-e**) LPC, PC and FC concentrations (**d**) and LPE and PE concentrations (**e**) of GF, OMM^12^ and SPF bile. Means +SD. GF: n = 26 mice, OMM^12^: n = 11 mice, SPF: n = 11 mice. *p < 0.05, **p < 0.01, ***p < 0.001, ****p < 0.0001, two-sided t-test with unequal variance. + indicates female, • indicates male. Source data

To analyse what processes of lipid flux are potentially affected by microbiota, lipid metabolism was modelled at the system level. We used the quantitative time courses of FA16:0[D5] and FA16:0[D31] determined in gut contents, plasma and six tissues to build a physiology-based kinetic model describing in vivo FA flux^12^ (Fig. 2a and Supplementary Data 1). It includes the parameters of gastrointestinal flow in the small and large intestine (*k*_p1_ and *k*_p2_), FA absorption from the gut lumen into intestinal tissue and further to blood (*k*_abs_), FA distribution from circulation to white adipose tissue (*k*_stor_), liver (*k*_hep_) and other tissues (modelled together as *k*_e_). We set up a basic model with six parameters, assuming them being microbiota-independent, and fit their values to FA16:0[D5] and FA16:0[D31] measurements in all model compartments for the three mouse groups simultaneously. To determine the effect of gut microbial presence on FA flux processes, for each of the model parameters, we tested whether defining this parameter as mouse group-specific improves the model performance. We built six models with eight parameters, where one of the parameters from the basic model is changed to three mouse group-specific parameters, and fitted parameter values to the same data as for the basic model. We found that setting the intestinal absorption parameter *k*_abs_ as group-specific substantially improves the model performance (mean squared error (MSE) reduction >20%, difference in Akaike information criterion (AIC) >50 compared with the basic model; Fig. 2b and Supplementary Data 1). This suggests that despite being penalized for increased complexity, the model with group-specific intestinal absorption coefficient best explains the differences in FA16:0[D5] and FA16:0[D31] kinetics in most tissues (Fig. 2c and Extended Data Fig. 2), with robust estimates of the corresponding parameters (Fig. 2d). The coefficient of intestinal FA absorption, *k*_abs_, is estimated to be twofold higher in GF mice compared with OMM^12^ or SPF mice (Fig. 2d). This is supported by the finding that 20 min after oral gavage, in duodenum tissue, 2.4–3.2% of total FA16:0 originated from absorption (FA16:0[D31] and FA16:0[D5]), whereas in OMM^12^ and SPF mice, it was only 1.3–1.6% and 1.0–1.4% (Extended Data Fig. 3a). *k*_p1_ specifying FA gastrointestinal flow in the small intestine was not different between the mouse groups, which was validated experimentally. Transit times of the lipid mix are similar in the small intestine of male and female GF, OMM^12^ and SPF mice (Extended Data Fig. 3b).

The advantage of using model selection with AIC is that it provides a balance between model complexity and its goodness-of-fit and helps to identify parameters, which, when added to the model, improve its fitting to experimental data across several tissues simultaneously. Our results indicate that the differences between labelled FA profiles observed in the three mouse groups across different tissues (Fig. 1b–f) can be best explained by reduced intestinal FA absorption in the presence of the microbiota.

### The gut microbiota impacts biliary glycerophospholipid levels relevant for intestinal FA absorption

Cluster of differentiation 36 (*Cd36*) and FA transporter 4 (*Slc27a4FATP4*) are relevant for FA transport into enterocytes^13^. *Cd36* mRNA is lower expressed in epithelial cells from the ileum of GF mice compared with conventional mice^14^. In our study, no difference in *Cd36* mRNA expression between GF, OMM^12^ and SPF mice in duodenum, ileum and jejunum tissue was found (Extended Data Fig. 3c). Palmitoylation of CD36 mediated by palmitoylacyltransferases zinc finger DHHC-type containing 4 and 5 (ZDHHC4 and ZDHHC5 and DHHC4 and DHHC5) affects its membrane localization and activity^15,16^. However, the gut microbiota, such as *Lactobacillus*, can affect CD36 palmitoylation^17^, and the G protein-coupled BA receptor 1 (Gpbar1, TGR5)^18^, for which deoxycholate is an agonist^19^, similar *Dhhc4* and *Dhhc5* mRNA expressions were detected in the duodenum, jejunum and ileum of GF, OMM^12^ and SPF mice (Extended Data Fig. 3c). *Slc27a4* mRNA expression was elevated in the duodenum and jejunum tissue of GF compared with OMM^12^ and SPF mice (Extended Data Fig. 3c), which could contribute to their higher intestinal absorption of FA16:0[D5] and FA16:0[D31].

**Extended Data Fig. 4:**
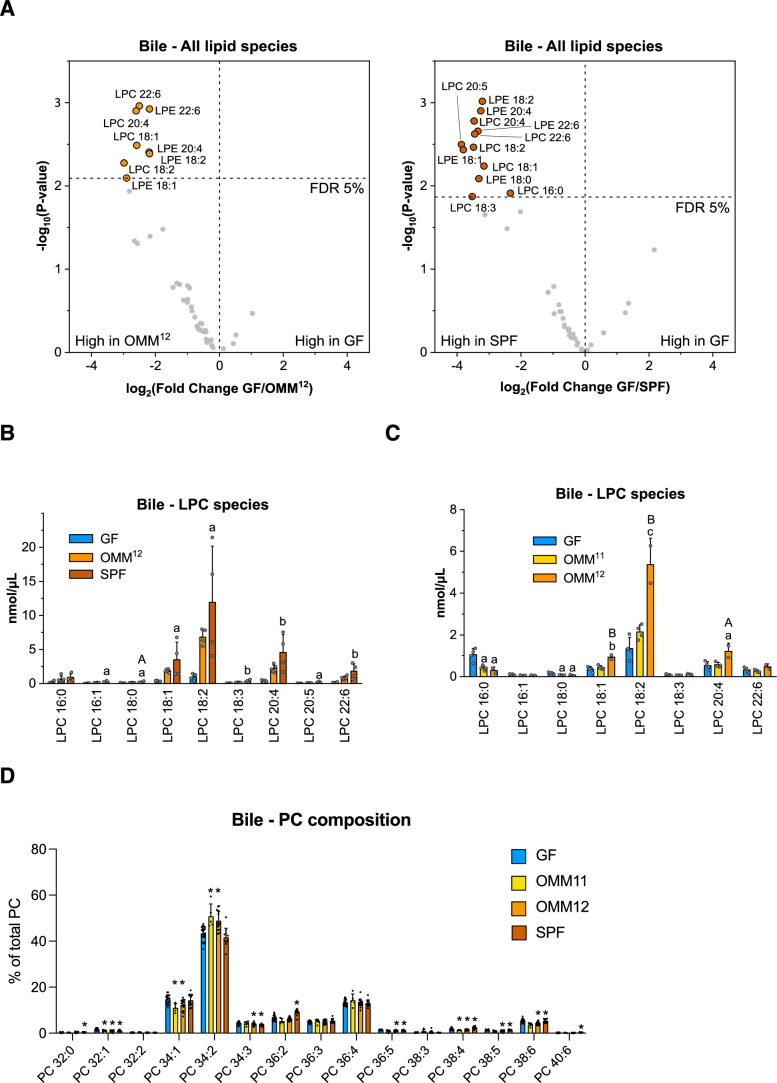
LPC and PC in bile (GF, OMM^11^, OMM^12^, SPF). (**a**) Volcano plots showing log_2_ fold changes of biliary lipid species for GF vs. OMM^12^ or SPF, 6 h post gavage. GF: n = 4, OMM^12^: n = 5, SPF: n = 4. Two-sided t-test with unequal variance and FDR correction at 5 %. (**b**) LPC levels in bile of GF, OMM^12^ and SPF mice. Means + SD. GF: n = 4 mice, OMM^12^: n = 5 mice, SPF: n = 4 mice. a/A p < 0.05, b/B p < 0.01, one-way ANOVA with Tukey post-hoc test. a, b significant vs. GF; A, B significant vs. OMM^12^. (**c**) LPC levels in bile of GF, OMM^11^ and OMM^12^ mice. Means + SD. GF: n = 4 mice, OMM^11^: n = 4 mice, OMM^12^: n = 2 mice. a p < 0.05 (vs. GF), one-way ANOVA with Tukey post-hoc test. (**d**) PC contents of bile of GF, OMM^11^, OMM^12^ and SPF mice. Means + SD. GF: n = 20 mice, OMM^11^: n = 5 mice, OMM^12^: n = 16 mice, SPF: n = 11 mice. *p < 0.05 (vs. GF), two-sided t-test with unequal variance. Source data

Next, we focused on bile, because it emulsifies luminal dietary lipids inducing the formation of micelles necessary for intestinal lipid absorption. The lipid composition of gallbladder bile depended on gut microbial colonization (Fig. 2e). Although GF mouse bile primarily contained PC, SPF bile was dominated by lysophosphatidylcholine (LPC) (Fig. 2f,g). The concentrations of lysophosphatidylethanolamine and LPC (Extended Data Fig. 3d,e), especially unsaturated LPC18:1, LPC18:2, LPC20:4 and LPC22:6 (Extended Data Fig. 4a,b), increased threefold from GF < OMM^12^ < SPF leading to altered PC/LPC (Fig. 2h).

**Fig. 3:**
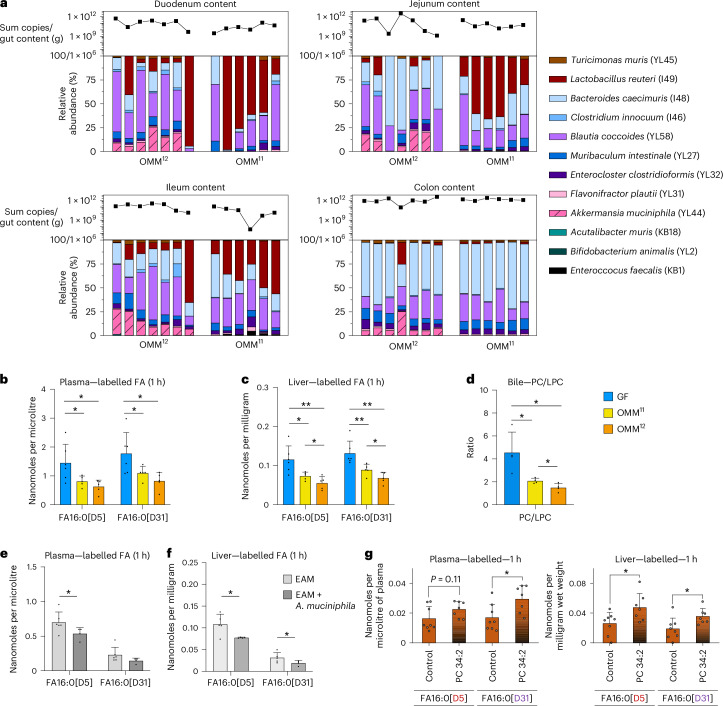
Systemic lipid uptake depends on biliary PC/LPC modulated by gut microbiota. **a**, The gut microbial load and composition in OMM^12^ and OMM^11^ gut content. OMM^12^, *n* = 7 mice, OMM^11^, *n* = 6 mice. **b**,**c**, The FA16:0[D5] and FA16:0[D31] levels in plasma (**b**) and liver (**c**) of GF, OMM^11^ and OMM^12^, 1 h after gavage with tracers. Means + s.d. GF, *n* = 6 mice, OMM^12^, *n* = 5 mice, OMM^11^, *n* = 5 mice. *P < 0.05, **P < 0.01, one-sided *t*-test with unequal variance. (**d**) PC/LPC in the bile of GF, OMM^11^ and OMM^12^, 1 h post-gavage. Means + s.d. GF, *n* = 4 mice, OMM^12^, *n* = 4 mice, OMM^11^, *n* = 5 mice. *P < 0.05, one-sided *t*-test with unequal variance. **e**,**f**, FA16:0[D5] and FA16:0[D31] levels in plasma (**e**) and liver (**f**) of EAM-colonized GF mice with or without *A. muciniphila*, 1 h after gavage with tracers. Means + s.d. EAM, *n* = 6 mice, EAM + *A. muciniphila*, *n* = 4 mice. *P < 0.05, one-sided *t*-test with unequal variance. **g**, The FA16:0[D5] and FA16:0[D31] in plasma and liver of SPF mice 1 h after gavage with tracers ± 100 nmol PC34:2. Means + s.d., control, *n* = 8 mice, control + PC34:2, *n* = 7 mice. *P < 0.05, two-sided *t*-test with unequal variance. • indicates male. Source data

To provide further evidence for these findings, we eliminated *Akkermansia muciniphila* from OMM^12^ to generate OMM^11^ mice. *A. muciniphila* was associated with reduced plasma lipid levels and lipid storage^20,21^, and 16S ribosomal RNA (16S rRNA) sequencing revealed it as an abundant member of the OMM^12^ microbiota in all gut segments (Fig. 3a). GF, OMM^11^ and OMM^12^ mice were gavaged with FA16:0[D5] and TG(FA16:0[D31])_3_. At 1 h later, we found that FA16:0[D5] and FA16:0[D31] levels in plasma and liver as well as biliary PC/LPC ratios could be ranked from highest to lowest: GF > OMM^11^ > OMM^12^ (Fig. 3b–d). Unsaturated LPC increased with gut microbiota diversity (Extended Data Fig. 4c). To support that *A. muciniphila* can affect systemic lipid absorption, mice colonized with easy accessible microbiota (EAM) without or with *A. muciniphila* were investigated. The EAM consortium consists of *Bacteroides thetaiotaomicron*, *E. coli* and *Eubacterium rectale*, representing the three most abundant bacterial phyla of human gut microbiota^22^. The addition of *A. muciniphila* to the EAM consortium decreased the amounts of FA16:0[D5] and FA16:0[D31] in plasma and liver 1 h after tracer gavage (Fig. 3e,f) providing further evidence that *A. muciniphila*, in addition to other microbial species, contributes to lipid absorption.

Following food intake, the gallbladder releases bile into the gut lumen. Thus, we tested whether luminal glycerophospholipid contents affect systemic lipid uptake in mice. Luminal PC enhances TG transport from the lumen to lymph^23^, and dietary and biliary PC promotes intestinal chylomicron output in rodents^24,25^. SPF mice were administered the tracer mix ± 100 nmol PC34:2 as major PC species in mouse bile, with slightly elevated contents in OMM^11^ and OMM^12^ compared with GF and SPF mice (Extended Data Fig. 4d). After 1 h, FA16:0[D5] and FA16:0[D31] levels were ~2.0-fold elevated in plasma and liver in the presence of PC34:2 (Fig. 3g).

We conclude that gut microbial presence reduces biliary PC contents, which decreases intestinal lipid uptake, and this effect can be modified by changing microbiota composition.

### PC is metabolized to LPC by PLA1 in bile

**Extended Data Fig. 5:**
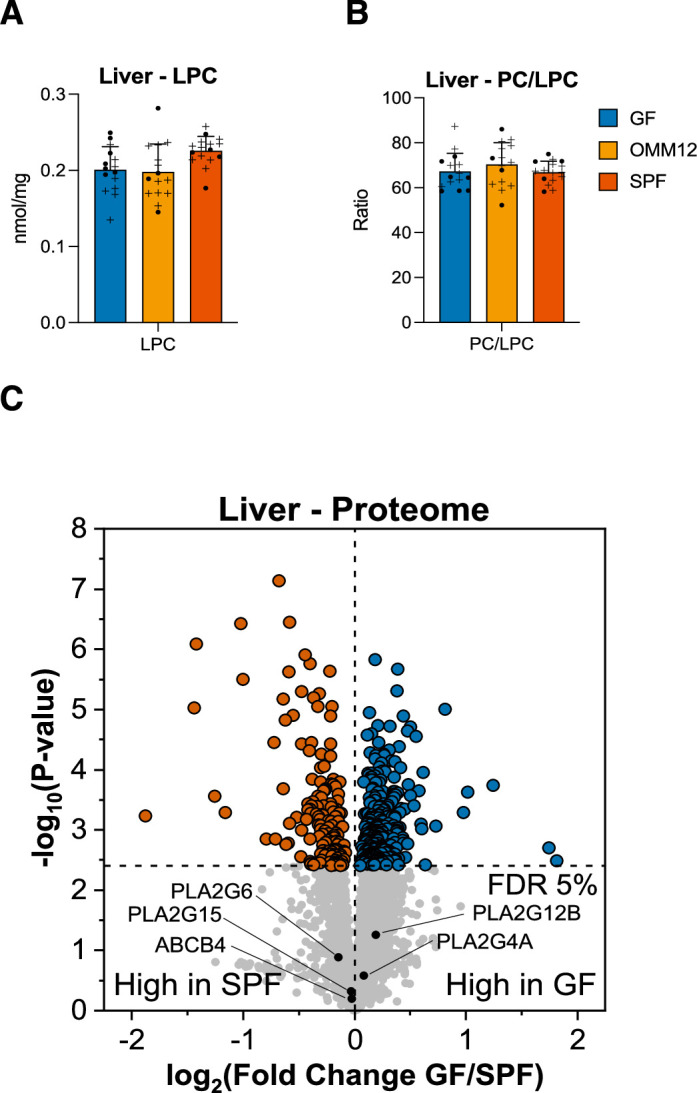
Total PC, PC/LPC (GF, OMM^12^, SPF) and proteome (GF, SPF) of liver. (**a-b**) LPC concentrations (**a**) and PC/LPC (**b**) in liver of GF, OMM^12^ and SPF. Means +SD. GF: n = 15 mice, OMM^12^: n = 15 mice, SPF: n = 16 mice. *p < 0.05 (vs. GF), two-sided t-test with unequal variance. (**c**) Volcano plot showing significantly different proteins in livers of GF vs. SPF. The dataset was described previously^6^. GF: n = 5, SPF: n = 5. Two-sided t-test with unequal variance and FDR correction at 5 %. + indicates female, • indicates male. Source data

PC can be metabolized to LPC by phospholipases in the liver, before the secretion of PC into the bile via ATP binding cassette B4 (ABCB4) transporter^26^. Comparing GF with colonized animals, we did not find differences in LPC levels and PC/LPC in liver or other tissues, except for bile (Fig. 2e and Extended Data Fig. 5a,b). In addition, protein abundances of hepatic ABCB4 and phospholipases were similar between colonized and GF animals (Extended Data Fig. 5c).

**Fig. 4:**
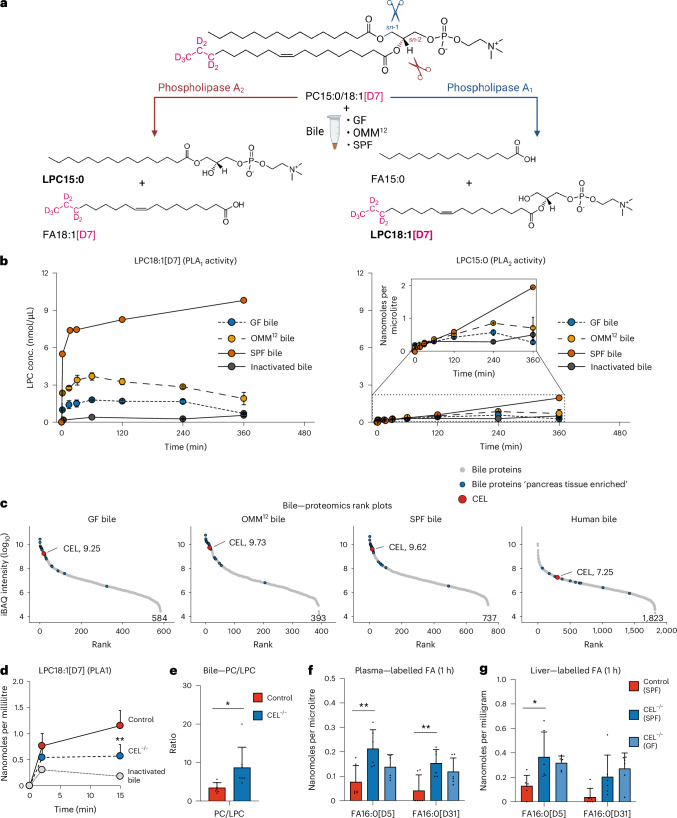
PC is metabolized to LPC in bile by a PLA1 activity of CEL. **a**, The PLA activity assay. Bold labels indicate the LPC species quantified as readouts of PLA activity. **b**, LPC18:1[D7] (PLA1) and LPC15:0 (PLA2) after incubation with GF, OMM^12^ or SPF bile. GF bile incubated for 15 min at 95 °C is the negative control. Means ± s.d. *N* = 3 technical replicates per timepoint and bile (GF, OMM^12^, SPF, GF heat inactivated). **c**, The rank order of proteins detected in GF, OMM^12^, SPF and human bile. CEL and pancreatic proteins are indicated. For mouse bile, samples were pooled from *n* = 6 mice per group (GF, OMM^12^, SPF). Human bile originates from *n* = 1 patient. **d**, LPC18:1[D7] (PLA1) after incubation with *Cel*^−/−^ or control bile (SPF). Control bile incubated for 15 min at 95 °C is the negative control. Means + s.d. Independent replicates per timepoint of control, *n* = 7, *Cel*^−/−^, *n* = 7, control heat inactivated, *n* = 4. *P < 0.05, **P < 0.01 two-sided *t*-test with unequal variance. **e**, The PC/LPC in the bile of *Cel*^−/−^ or controls (SPF). Means + s.d. Control (SPF), *n* = 7 mice, Cel^−/−^ (SPF), *n* = 7 mice. *P < 0.05, two-sided *t*-test with unequal variance. **f**,**g**, FA16:0[D5] and FA16:0[D31] levels in plasma (**f**) and liver (**g**) of SPF controls, SPF *Cel*^−/−^ and GF *Cel*^−/−^ *mice*, 1 h after gavage. Means + s.d. Control (SPF), *n* = 6 mice, *Cel*^−/−^ (SPF), *n* = 6 mice, *Cel*^−/−^ (GF), *n* = 6 mice. *P < 0.05, **P < 0.01, two-sided *t*-test with unequal variance. • indicates male. Source data

**Extended Data Fig. 6:**
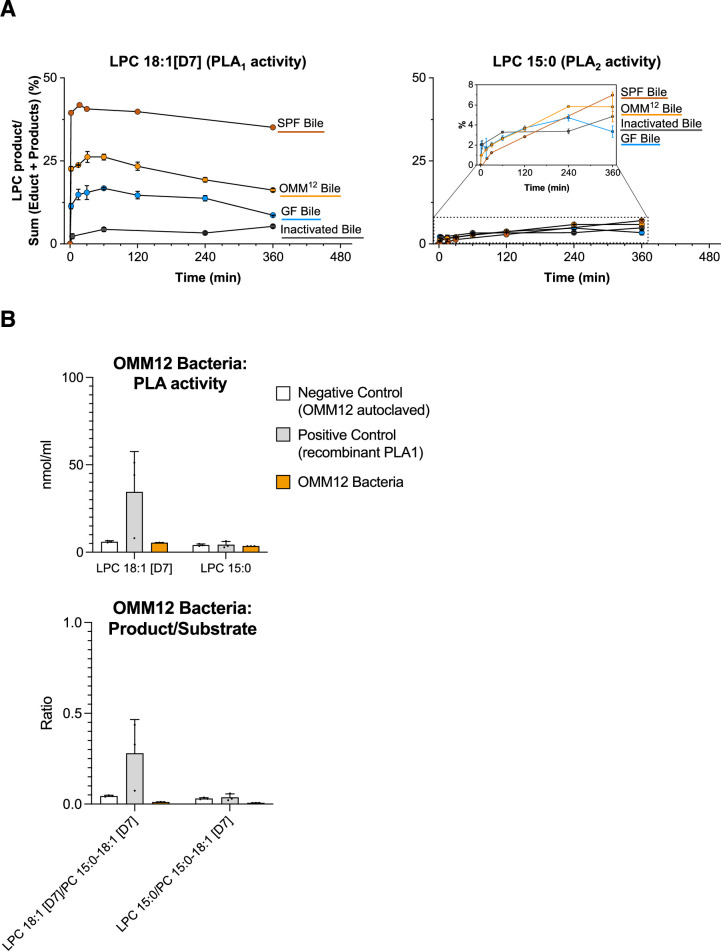
PLA1 and PLA2 activities in bile of GF, OMM^12^ and SPF mice, and of OMM^12^ bacteria. (**a**) Percentage of PC15:0-18:1[D7] substrate converted to LPC18:1[D7] (PLA1) and LPC15:0 (PLA2) after incubation for 2–360 min with GF, OMM^12^ or SPF bile. GF bile incubated for 15 min at 95 °C was used as a negative control (Inactivated Bile). Means ± SD. N = 3 technical replicates per time-point and bile (GF, OMM^12^, SPF, GF heat inactivated). (**b**) LPC18:1[D7] (PLA1) and LPC15:0 (PLA2) levels; and LPC 18:1[D7]/PC15:0-18:1[D7] (PLA1) and LPC15:0/PC15:0-18:1[D7] (PLA2) ratios after 24 h incubation of PC15:0-18:1[D7] with active OMM^12^ bacteria, autoclaved OMM^12^ bacteria (negative control) or OMM^12^ bacteria plus added PLA1 from *Aspergillus oryzae* (positive control). Means +SD. N = 3 independent incubations per condition (active OMM^12^ bacteria, negative control, positive control). Source data

To test whether LPC is generated in the bile, mouse bile samples were incubated with PC15:0/18:1[D7] at 10 nmol µl^−1^ (equivalent to total PC amounts of SPF bile) (Fig. 4a). The concentrations of the phospholipase A (PLA)1 and PLA2 products, LPC18:1[D7] and LPC15:0, respectively, were measured after 2–360 min of incubation using high-resolution mass spectrometry (HR-MS) (Fig. 4b). In SPF animals, after 2 min more than 40% of the substrate was metabolized to LPC18:1[D7] (PLA1) (Extended Data Fig. 6a). SPF PLA1 activities were ~2-fold higher than those of OMM^12^ mice and ~4-fold higher than those of GF mice (Fig. 4b), explaining the differences in PC/LPC between the groups (Fig. 2h). Neither LPC15:0, nor LPC18:1[D7] was detected in heat-treated GF bile (Fig. 4b) verifying that PC to LPC metabolism depends on enzyme activity.

To exclude that the gut microbiota is a relevant source of PLA activity in the gut lumen, OMM^12^ bacteria were incubated with PC15:0/18:1[D7] for 24 h, revealing no substantial degradation to LPC18:1[D7] (PLA1) or LPC15:0 (PLA2) (Extended Data Fig. 6b). The result was identical when autoclaved OMM^12^ bacteria were incubated with PC15:0/18:1[D7] (negative control), whereas the addition of a recombinant PLA1 induced LPC18:1[D7] generation (positive control), demonstrating that no microbial PLA activity is present.

To identify PLA proteins, the bile proteome was analysed. In total, 393–737 proteins were detected (Fig. 4c and Supplementary Data 2). Only one protein with a known PLA activity was identified: carboxyl ester lipase (CEL), found among the 50 most abundant proteins, with similar amounts in GF, OMM^12^ and SPF bile. CEL, formerly known as bile salt-stimulated lipase having a broad substrate specificity including PC^27^, could also be detected in human bile. It is synthesized in the pancreas, consistent with the large fraction of pancreatic proteins enriched in bile. To confirm an involvement of CEL in gut microbiota-dependent lipid absorption, mice with a global CEL deficiency were bred. An investigation of PLA1 activity in bile after incubation with PC15:0/18:1[D7] for 2 and 15 min revealed a reduction when CEL was absent (Fig. 4d). LPC18:1[D7] levels were twofold lower in CEL-deficient compared with control mice. Accordingly, PC/LPC was 2.5-fold higher in CEL knockouts (KOs) than in controls (Fig. 4e). At 1 h after gavage with FA16:0[D5] and TG(16:0[D31])_3_, the concentrations of FA16:0[D5] and FA16:0[D31] were 2.5–5.0-fold elevated in plasma and liver in SPF CEL-KO mice compared with SPF controls, demonstrating that CEL is involved in lipid absorption (Fig. 4f,g). The result that FA16:0[D5] and FA16:0[D31] levels were not different in GF CEL-KO and SPF CEL-KO mice indicates that the gut microbiota limits systemic lipid absorption via CEL.

These data show that gallbladder bile contains a considerable PLA1 activity that is highest in SPF mice, followed by OMM^12^ and GF animals. The most likely PLA1 candidate is CEL, because it is the only protein with known phospholipase activity detected in bile samples. CEL deficiency decreases PLA_1_ activity in bile and increases systemic lipid uptake in SPF mice.

### Biliary PLA1 activity is stimulated by TCA depending on gut microbial colonization

**Fig. 5:**
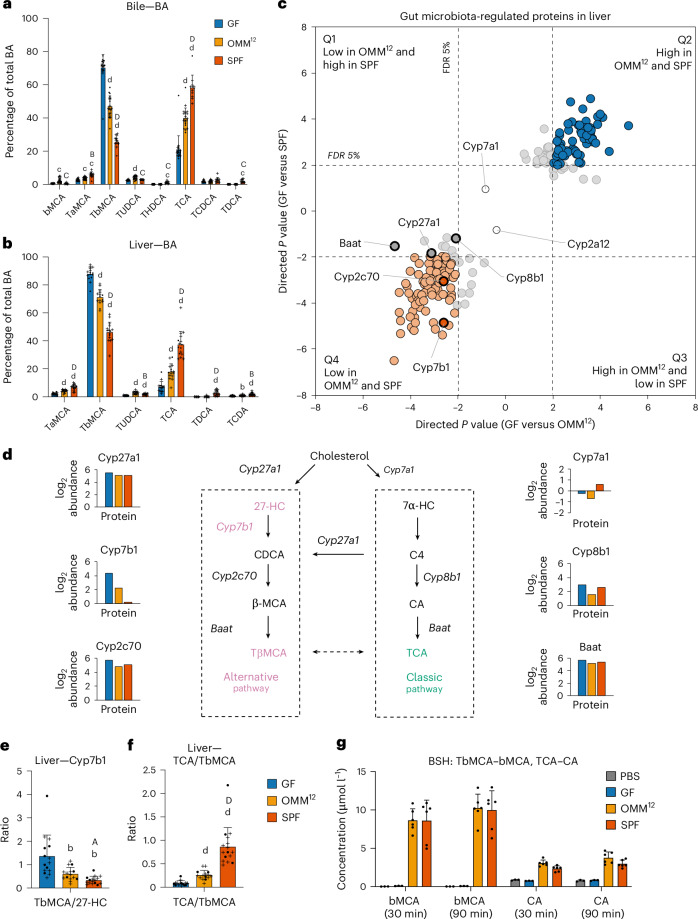
The gut microbiota alters BA metabolism in bile and liver by inhibition of hepatic CYP7B1. **a**, The BA in bile of GF, OMM^12^ and SPF (with contributions>1%). Means + s.d. GF, *n* = 19 mice, OMM^12^, *n* = 20 mice, SPF, *n* = 11 mice. a/A, *P* < 0.05; b/B, *P* < 0.01; c/C, *P* < 0.001; d/D, *P* < 0.0001, two-sided *t*-test with unequal variance. **b**, The BA in liver of GF, OMM^12^ and SPF (with contributions >1%). Means + s.d. GF, *n* = 15 mice, OMM^12^, *n* = 15 mice, SPF, *n* = 16 mice. a/A, *P* < 0.05; b/B, *P* < 0.01; c/C, *P* < 0.001; d/D, *P* < 0.0001, two-sided *t*-test with unequal variance. **c**, The integration of significant different proteins identified in the comparisons GF versus OMM^12^ and GF versus SPF. GF, *n* = 3, OMM^12^, *n* = 3 mice, SPF, *n* = 3 mice. Two-sided *t*-test with unequal variance and FDR correction at 5%. Directed *P* values are defined as ‘–log_10_(*P*) times the direction of the effect’. **d**, The classic and alternative BA synthesis pathways. Bar plots indicate hepatic protein abundances as log_2_ determined with proteomics in GF, OMM^12^ and SPF mice. Means, *n* = 3 per group. **e**, 27-HC/TβMCA in liver. Means + s.d. GF, *n* = 15 mice, OMM^12^, *n* = 15 mice, SPF, *n* = 16 mice. a/A, *P* < 0.05; b/B, *P* < 0.01, two-sided *t*-test with unequal variance. **f**, The TCA/TβMCA in liver of GF, OMM^12^ and SPF mice. Means + s.d. GF, *n* = 15 mice, OMM^12^, *n* = 15 mice, SPF, *n* = 16 mice. d/D, *P* < 0.0001, two-sided *t*-test with unequal variance. **a**,**b**,**e**,**f**, SPF versus GF (a, b, c, d) or OMM^12^ (A, B, C, D). **g**, The BSH activity: levels of βMCA and CA generated from TβMCA and TCA after incubation with caecal contents of GF, OMM^12^ and SPF mice or with PBS (as negative control) for 30 and 90 min. Means + s.d. Independent replicates per timepoint and incubation, PBS, *n* = 3, GF, *n* = 3, OMM^12^, *n* = 6, SPF, *n* = 6. + indicates female, • indicates male. Source data

**Extended Data Fig. 7:**
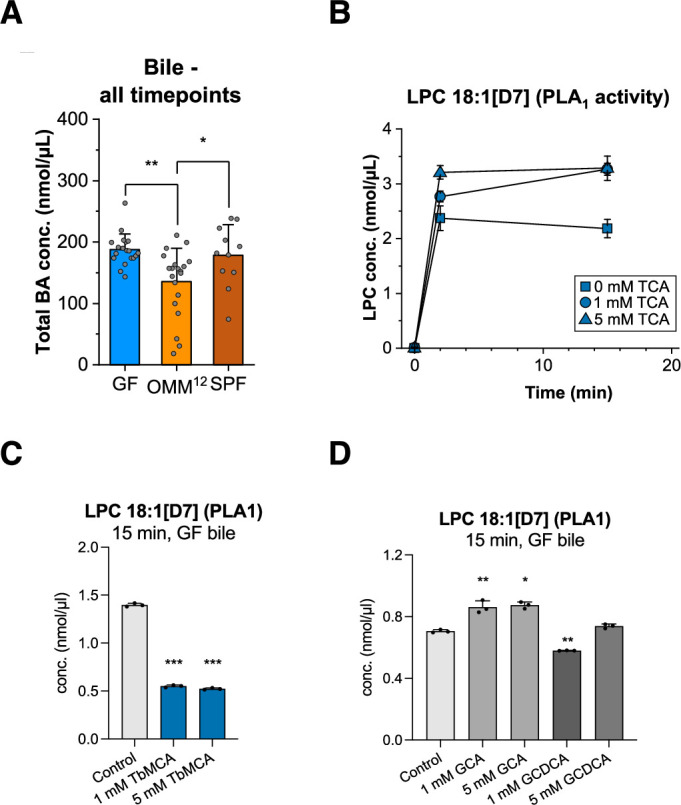
Total bile acid levels in bile (GF, OMM^12^, SPF) and the effect of bile acids on PLA1 activity. (**a**) Total BA concentrations in the bile of GF, OMM^12^ and SPF mice. Means + SD. GF: n = 19 mice, OMM^12^: n = 20 mice, SPF: n = 11 mice. *p < 0.05, **p < 0.01, one-way ANOVA with Tukey post-hoc test. (**b**) LPC18:1[D7] levels (PLA1) after incubation of GF bile ±1 of 5 mM TCA. Means ± SD. N = 3 technical replicates per time-point and condition. (**c-d**) LPC18:1[D7] levels (PLA1) after incubation with GF bile ±1–5 mM TβMCA (**c**) or ±1–5 mM GCA and GCDCA (**d**). Means +SD. N = 3 independent incubations per condition (control, 1–5 mM TβMCA, 1–5 mM GCA, –5 mM GCDCA). *p < 0.05, **p < 0.01, ***p < 0.001, relative to the control groups, two-sided t-test with unequal variance. Source data

CEL activity depends on taurocholic acid (TCA)^28,29^. To investigate whether a TCA-stimulated PC to LPC conversion might explain our findings, we quantified BA in GF, OMM^12^ and SPF bile. Although total BA levels were similar (Extended Data Fig. 7a), their composition differed (Fig. 5a). Whereas GF bile mainly contained tauro-β-muricholic acid (TβMCA), SPF bile was dominated by TCA, suggesting that TCA stimulates PC to LPC conversion. This is confirmed by the finding that TCA elevates PLA1-mediated LPC18:1[D7] generation from PC15:0/18:1[D7] in GF bile (Extended Data Fig. 7b).

**Extended Data Fig. 8:**
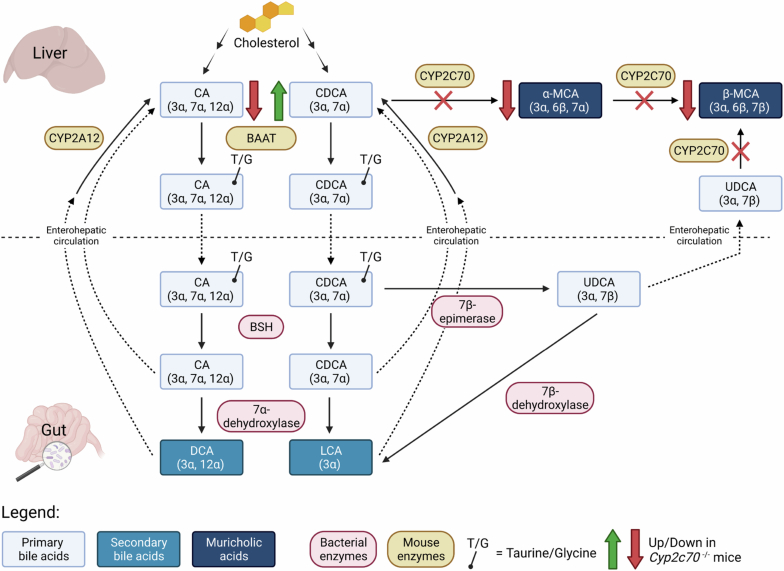
Bile acid metabolism in *Cyp2c70* KO mice. Scheme illustrating bile acid synthesis and conversion in mice as well as the consequences of *Cyp2c70* KO. CA and CDCA are synthesized by the liver from cholesterol. They are mainly conjugated to taurine and to a minor degree to glycine by bile acid-CoA:amino acid N-acyltransferase (BAAT). In the gut, conjugated BAs are deconjugated by bacterial bile salt hydrolases (BSH) and can be further metabolized by bacterial enzymes before entering the enterohepatic circulation. The muricholic acid α-MCA is generated from CDCA via 6β-hydroxylation and converted into β-MCA by 7β-epimerization. As a minor pathway, UDCA can also be converted into β-MCA. The enzyme CYP2C70 catalyzes these reactions. In the bile of *Cyp2c70* KO mice, muricholic acids (MCA) are absent while the concentration of the main MCA precursor CDCA is increased. CA concentration in *Cyp2c70* KO mice is reduced due to the lower expression of CYP8B1, a key enzyme for CA synthesis in the liver. CA: Cholic acid, CDCA: Chenodeoxycholic acid, α-MCA: α-Muricholic acid, β -MCA: β -Muricholic acid, DCA: Deoxycholic acid, LCA: Lithocholic acid, UDCA: Ursodeoxycholic acid. Figure created in BioRender; Ecker, J. https://biorender.com/2is2fow (2026).

**Extended Data Fig. 9:**
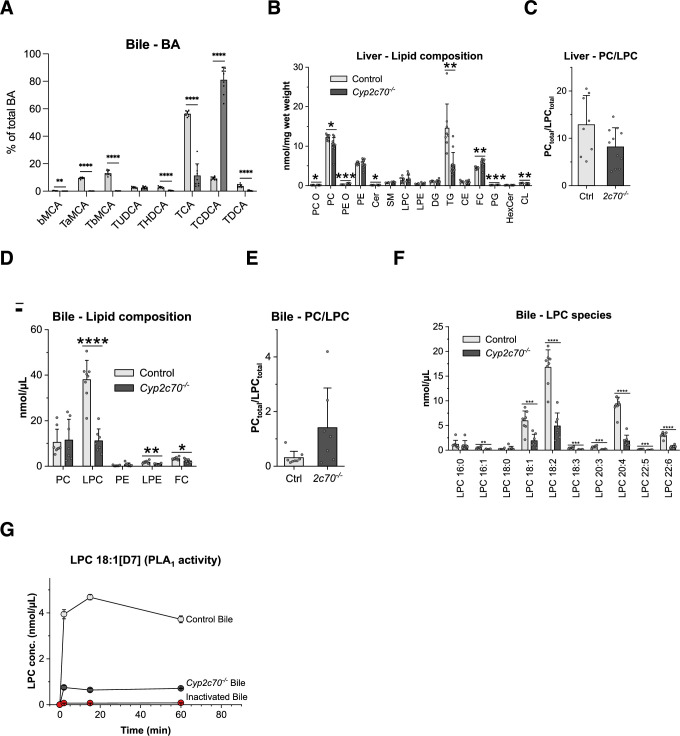
Bile acids and PLA1 activity in bile, lipid composition in liver and bile of *Cyp2c70* KO mice. (**a**) BA in bile of *Cyp2c70 −/−* and controls (with contributions>1%). Means + SD. Control: n = 9 mice, *Cyp2c70 −/−*: n = 8 mice. **p < 0.05, **p < 0.01, ***p < 0.001, ****p < 0.0001, two-sided t-test with unequal variance. (**b-c**) Lipid class composition (**b**) and PC/LPC (C) *Cyp2c70 −/−* and controls. Means + SD. Control: n = 8 mice, *Cyp2c70 −/−*: n = 11 mice. **p < 0.05, **p < 0.01, ***p < 0.001, two-sided t-test with unequal variance. (**d-e**) Lipid class composition and PC/LPC ratio in bile from *Cyp2c70 −/−* and control livers. Means + SD. Control: n = 8 mice, *Cyp2c70 −/−*: n = 7 mice. **p < 0.05, **p < 0.01, ***p < 0.001, two-sided t-test with unequal variance. (**f**) LPC species levels in bile of *Cyp2c70−/−* mice and controls. Means + SD. Control: n = 8 mice, *Cyp2c70 −/−*: n = 7 mice. **p < 0.05, **p < 0.01, ***p < 0.001, ****p < 0.0001, two-sided t-test with unequal variance. (**g**) LPC18:1[D7] levels (PLA1) after incubation for 2–60 min with *Cyp2c70 −/−* or control bile. Control mouse bile incubated for 15 min at 95 °C is the negative control. Means ± SD. N = 3 technical replicates per time-point and bile (control, *Cyp2c70 −/−*, inactivated). Source data

To substantiate this hypothesis, we investigated biliary PC metabolism in mice with a gene deficiency in cytochrome P450 2c70 (*Cyp2c70*) generating 6-hydroxylated BA (Extended Data Fig. 8)^30^. In *Cyp2c70*-KO mice, in bile, TβMCA was absent and TCA levels were tenfold lower than in controls (Extended Data Fig. 9a). Although LPC levels and PC/LPC were unchanged in liver (Extended Data Fig. 9b,c), in bile, LPC concentrations, particularly those of unsaturated species, were reduced (Extended Data Fig. 9d–f). PC15:0/18:1[D7] to LPC18:1[D7] (PLA1) conversion was ~5-fold reduced in the bile of *Cyp2c70*-KO mice (Extended Data Fig. 9g).

Of note, addition of TβMCA, whose levels are strongly lowered in bile of SPF and OMM^12^ mice (Fig. 5a) reduced PLA1-mediated LPC18:1[D7] generation from PC15:0/18:1[D7] almost twofold (Extended Data Fig. 7c). Glyco-conjugated cholic acid, a major human BA, increases PLA1 activity by 20%, whereas glyco-conjugated chenodeoxycholic acid had no effect (Extended Data Fig. 7d).

These findings demonstrate that, in bile, PC to LPC conversion by PLA1 is stimulated by TCA and inhibited by TβMCA, whose levels depend on microbial colonization.

### The gut microbiota increases TCA contents by CYP7B1-dependent decrease of alternative BA synthesis in liver

**Extended Data Fig. 10:**
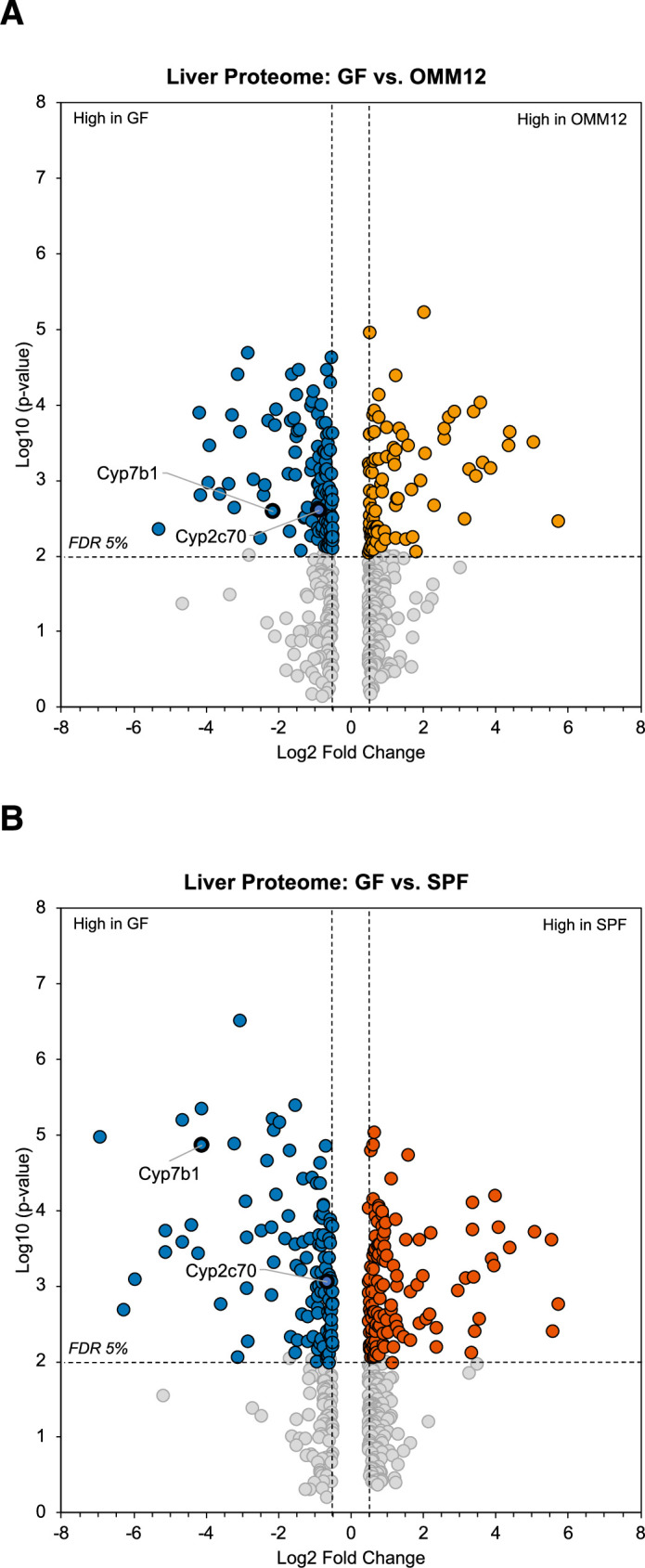
Liver proteome of GF, OMM^12^ and SPF mice. Volcano plots showing significantly different proteins (Log2FC > 0.5 or <−0.5) in livers of (**a**) GF vs. OMM^12^ and (**b**) GF vs. SPF mice. The datasets are included as Supplementary Data 2. GF: n = 3 mice, OMM^12^: n = 3 mice, SPF: n = 3 mice, two-sided t-test with unequal variance and FDR correction at 5%. Source data

To clarify how the gut microbiota modulates biliary TCA, we investigated BA synthesis in liver secreting BA into bile. The differences of TCA and TβMCA fractions between GF, OMM^12^ and SPF mice in liver (Fig. 5b) were similar to the ones observed in bile (Fig. 5a). To get a more comprehensive view, the liver proteome was quantified. In total, 6102 proteins were identified (Supplementary Data 2), with 221 being differentially abundant (log_2_(FC) >0.5 or <−0.5, false discovery rate (FDR) <0.05) between GF and OMM^12^ mice and 239 between GF and SPF mice (Extended Data Fig. 10a,b). To find verified candidates regulated by the gut microbiota, we combined the results for both comparisons resulting in 61 overlapping proteins that were up- (Fig. 5c, Q2) and 87 overlapping proteins that were downregulated significantly (Fig. 5c, Q4) in SPF and OMM^12^ versus GF groups, including two candidates of BA metabolism, CYP7B1 and CYP2C70.

BA are synthesized from cholesterol by: (1) the classic pathway generating chenodeoxycholic acid (CDCA) by cytochrome P450 family 27 subfamily A member 1 (CYP27A1) or (T)CA by cytochrome P450 family 8 subfamily B member 1 (CYP8B1) and (2) the alternative pathway, in which CYP7B1 generates CDCA that is metabolized to (T)βMCA by CYP2C70 in mice (Fig. 5d). CYP7B1 is the rate-limiting enzyme of the alternative pathway^7^. Its protein abundance decreased with gut colonization (Fig. 5d), as well as the ratio between its product and substrate, TβMCA and 27-hydroxycholesterol (27-HC; Fig. 5e). We conclude that the reduced abundance of CYP7B1 is a major driver for the concomitant increase of the TCA to TβMCA ratio (Fig. 5f).

To test the activity of bacterial bile salt hydrolase (BSH) cleaving the amide bond linking BA to taurine, caecal contents of OMM^12^ and SPF mice were incubated with TβMCA and TCA for 30 and 90 min, revealing similar β-muricholic acid (βMCA) and cholic acid (CA) levels (Fig. 5g). BSH activity for TβMCA and TCA in GF mice was identical as in the incubations with phosphate-buffered saline (PBS) (negative control). We conclude that increase of the TCA to TβMCA ratio in GF > OMM^12^ > SPF is not related to bacterial BSH. Instead, the major driver is the decreased abundance of CYP7B1 associated with gut microbial colonization.

### *CYP7B1*, the ratio between TCA and TβMCA in liver as well as PLA1 in bile are modulated by the MYD88 signalling axis

**Fig. 6:**
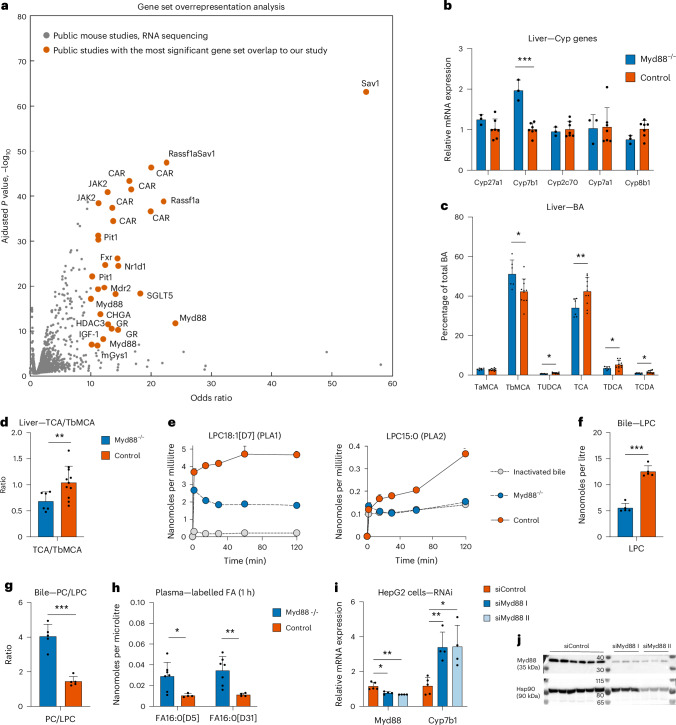
The gut microbiota modulates hepatic Cyp7b1 and biliary PLA1 via MYD88. **a**, A scatter plot depicting odds ratio versus adjusted *P* value for Fisher’s exact test comparing gene sets from each of the comparisons from the publicly available studies to the set of differentially abundant proteins in the liver of colonized versus GF mice. Orange shows comparisons with adjusted *P* value <0.001, odds ratio >10 and number of overlapping genes >10. Labels indicate mouse genotypes reported in each comparison. **b**, The hepatic mRNA expression of Cyp genes in *Myd88*-KO and control mice. Means + s.d. *Myd88* KO, *n* = 3 mice, control, *n* = 7 mice. ***P < 0.001, two-sided *t*-test with unequal variance. **c**, The BA in liver of *Myd88*-KO and control mice (with contributions >1%). Means + s.d. *Myd88* KO, *n* = 6 mice, control, *n* = 11 mice. *P < 0.05, **P < 0.01, two-sided *t*-test with unequal variance. **d**, The TCA/TβMCA in liver of *Myd88*-KO mice and controls. Means + s.d. *Myd88* KO, *n* = 6 mice, control, *n* = 11 mice. *P < 0.05, **P < 0.01, two-sided *t*-test with unequal variance. **e**, LPC18:1[D7] and LPC15:0 levels after incubation for 2–120 min with *Myd88*^−^-KO or control bile. The control mouse bile at 95 °C for 15 min is a negative control. *N* = 3 technical replicates per timepoint and bile type (control, *Myd88* KO, control heat inactivated). **f**,**g**, LPC levels (**f**) and PC/LPC (**g**) in bile of *Myd88*-KO and controls. Means + s.d. *Myd88* KO, *n* = 5 mice, control, *n* = 5 mice. *P < 0.05, **P < 0.01, ***P < 0.001, two-sided *t*-test with unequal variance. **h**, The FA16:0[D5] and FA16:0[D31] levels in plasma of *Myd88*-KO mice and controls, 1 h after gavage. Means + s.d. *Myd88* KO, *n* = 7 mice, control, *n* = 4 mice. *P < 0.05, **P < 0.01, two-sided *t*-test with unequal variance. **i**, The *Myd88* and *Cyp7b1* mRNA expression of HepG2 cells treated with a non-targeting control siRNA (siControl) or two different siRNA against *Myd88* (siMyd88I/II). Means + s.d. Independent incubations of siControl, *n* = 5, siMyd88I, *n* = 4, siMyd88II, *n* = 4. *P < 0.05, **P < 0.01, two-sided *t*-test with unequal variance. **j**, The MYD88 and HSP90 (housekeeper) protein expression of after immunoblot analyses of HepG2 cells treated with a siControl or two different siRNA against *Myd88* (siMyd88I/II). Independent incubations of siControl, *n* = 6, siMyd88I, *n* = 3, siMyd88II, *n* = 3. • indicates male. Source data

To investigate how the gut microbiota modulates *Cyp7b1*, we compared differentially abundant proteins in colonized mice (*n* = 191, abs(log(FC)) ≥ log_2_(1.5), FDR <0.05 in both SPF and OMM^12^ groups versus GF) to differentially genes from publicly available datasets. We retrieved 216 RNA sequencing and 528 Affymetrix Microarray datasets from Expression Atlas^31^ and performed overrepresentation analysis between the sets of differentially expressed genes using Fisher’s exact test. In the top 30 of the most similar expression profiles, we found multiple occurrences of mouse groups with deletions in salvador family WW domain containing protein 1 (*Sav1*), nuclear receptor subfamily 1 group 1 member 3 (*Nr1i3*, constitutive androstane receptor (*Car*)), Janus kinase 2 (*Jak2*), Ras association domain family member 1a (*Rassf1a*) and *Myd88* genes (Fig. 6a). As the adaptor protein MYD88 is crucial for sensing gut microbes via Toll-like receptors (TLRs)^32^, and a hepatocyte-specific deletion elevates *Cyp7b1* mRNA expression^33^, it was considered as most promising candidate. We could detect a twofold induced *Cyp7b1* transcription (Fig. 6b) and reduced TCA but increased TβMCA contents in livers of mice with a *Myd88* global gene deficiency compared with those from control animals (Fig. 6c) and a shift towards classical BA synthesis indicated by the lowered TCA/TβMCA (Fig. 6d). Tauro ursodeoxycholic acid (TUDCA) and tauro deoxycholic acid (TDCA) contents were reduced in livers of *Myd88*-KO animals compared with controls, as observed for the GF versus SPF comparison (Fig. 6c). An analysis of PLA1 activity in bile after incubation with PC15:0/18:1[D7] for 2–120 min revealed more than 50% lower LPC18:1[D7] levels in *Myd88*-KO compared with control animals (Fig. 6e). Total LPC levels were 2.3-fold increased but PC/LPC 2.8-fold decreased in *Myd88*-KO animals as well as the systemic uptake of FA16:0[D5] and FA16:0[D31] (Fig. 6f–h) strengthening a relevance of MYD88 for lipid absorption. To confirm an involvement of *Myd88* in regulation of *Cyp7b1* expression, we knocked down *Myd88* in the HepG2 hepatocyte cell line (Fig. 6i–j). In cells treated with small interfering RNAs (siRNAs) against *Myd88*, *Cyp7b1* transcription was 3.5-fold increased, which is in line with mRNA expression profiles detected in liver samples from *Myd88*-KO animals (Fig. 6b).

These results show that MYD88 signalling modulates *Cyp7b1* expression, the ratio between TCA and TβMCA in liver and PLA1-dependent PC to LPC degradation in bile.

## Discussion

We unveiled that gut microbiota regulates enzymes in gallbladder bile to modify intestinal uptake of dietary lipids. The amount of lipids entering the circulation (based on the AUCs of FA16:0[D5] and FA16:0[D31]) was reduced in OMM^12^ by 45–51% and in SPF by 58–63% compared with GF mice within 6 h. This reduction is much larger compared with effects of anti-obesity drugs. Orlistat, a gastric and pancreatic lipase inhibitor, lowers dietary lipid absorption by ~30%^13^, and semaglutide, a glucagon-like peptide 1 (GLP-1) analogue, reduces postprandial TG accumulation in serum by ~24%^34^. Recombinant GLP-1 alleviates intestinal absorption of triolein by ~10–24% in rats^35^.

We aimed to investigate lipid flux as close to physiological conditions as possible and applied stable isotope-labelled lipids that can be traced with mass spectrometry. Comparing our results with those from others, the individual experimental conditions must be considered. Wang and colleagues reported that GF mice fed a high-fat diet contained lower amounts of unsaturated FAs in their intestinal epithelial cells compared with those of conventional mice using a colorimetric assay, which does not detect saturated FA^36^. The application of lipoprotein lipase (LPL) inhibitor blocking peripheral lipid uptake before gavage of [^3^H]-labelled TG, revealed a higher accumulation of [^3^H] over 7 h in plasma of SPF compared with GF on a chow and high-fat diet^9^. Surprisingly, [^3^H]-TG uptake kinetics were largely similar in plasma of LPL inhibitor treated and untreated mice, in which, after a first peak, a decrease of systemic lipids would be expected owing to flux in peripheral tissues, as observed here. Gao et al., showed that gut microbiota-induced interleukin-22 production in small intestinal T cells reduces absorption of [^3^H]-labelled TG in enterocytes of mice fed a standard diet, which can be restored after antibiotic treatment^37,38^. Although others showed that microbial colonization of GF mice decreases whole gut transit times^39,40^, here, the transit times of the gavaged lipid mix in the small intestine, which is most relevant for systemic FA uptake, were similar in GF, OMM^12^ and SPF mice.

Astonishingly, we identified 393–1,823 proteins in gallbladder bile. We proved a PLA1 activity of CEL regulated by the gut microbiota. In CEL-deficient SPF mice, PLA1 activity in bile was reduced and the lipid uptakes in plasma and liver increased, but similar to those determined in GF CEL-KO mice. CEL, synthesized in the exocrine pancreas^27^, could reach the bile via the pancreatic duct draining into the common bile duct^41^. It requires BA containing 3α,7α-OH groups, in particular TCA (3α,7α,12α-OH)^27,28^, which binds to CEL’s active site inducing a hydrophobic pocket for substrates^29^. In SPF bile, TCA is the major BA and positively correlates with LPC levels. Human bile contains high amounts of glyco-conjugated 3α,7α-OH-containing CA (GCA)^42^, which stimulates PLA1 activity and comprises CEL, implying a potential translational relevance of our findings.

Lipid uptake was not tested in *Cyp2c70*-KO mice owing to their drastically altered BA profiles, per se affecting intestinal lipid absorption^43^. Deficiency in *Cyp2c70* reduces 12α-OH BAs, which are important for micelle formation, and female KO mice have liver pathologies^30,43^. Such cofounders complicate also the comparison with lipid flux studies taken out in *Cyp7b1*-^44^, *Cyp8b1*-^45^, *Cyp2c70*-^43^ and *Cyp2c*-KO^46^ mice. *Cyp7b1* KOs have threefold lower total circulating BA levels and an increased risk for liver disease^44^. *Cyp8b1*-KO causes a MCA-dominated BA profile and eliminates 12α-OH BAs^45^. *Cyp2c-*deficiency depletes MCAs, reduces 12α-OH BAs and induces liver damage^46^. Although sex hormones are well known to affect BA and lipid metabolism^47^, we did not find differences in lipid uptake between female and male mice, which were mixed for several experiments of this study.

Gut microbial colonization shifted BA metabolism towards the classic pathway by downregulating *Cyp7b1*. Further, we show that *Cyp7b1* (also reported previously^33^), the ratio between TCA and TβMCA and PLA1 activity are modulated by MYD88. Other regulators of microbiome-dependent BA metabolism might be farnesoid X receptor (FXR) and CAR. However, FXR is only activated in the intestine by the gut microbiota, but not in the liver^48,49^. In liver, FXR induces small heterodimer partner, which represses *Cyp7a1* and *Cyp8b1* and decreases classic BA synthesis^7^. CAR is activated by diindoles produced by the gut microbiota^50^, and CAR ligands can increase *Cyp7a1* expression and TCA levels in liver^51^. Our results indicate the opposite, a gut microbiota-induced shift to BA synthesis via the classic pathway.

Kinetic multi-compartment modelling revealed that intestinal FA absorption is modulated by the gut microbiota. Micelle formation in the gut lumen depends on biliary PC, which enlarges the lipid–water interfacial area by decreasing the emulsified particles size leading to less interfacial tensions. The emulsification capacity of PC in the intestinal lumen is magnitudes higher than that of BA^52^, yet the concentrations of BA are ~10-fold higher than those of PC, suggesting that biliary PC contents and BA composition determine intestinal FA absorption. A function of TCA is to make dietary lipids available for PNLIP^53^. Our results show that TG hydrolysis is not involved in the gut microbiota’s influence on lipid uptake, because the time profiles for FA16:0[D5] and TG-derived FA16:0[D31] were similar in all groups. We could detect labelled FA18:0 in gut contents, suggesting an elongation by the host and entry into enterohepatic circulation or by the gut microbiota. However, the observed levels of FA18:0[D5/31] are 100-fold lower than those of FA16:0[D5/31] and, thus, cannot explain the differences in lipid uptake between GF and colonized mice. We speculate that intestinal absorption of cholesterol might be stimulated by gut microbiota-dependent PC degradation in bile, because luminal PC interferes with cholesterol absorption^54–56^.

In the context of obesity or pathologies with excessive fat intake as a risk factor, including intestinal cancer^57^, manipulating the microbiome to limit intestinal lipid absorption might offer new therapeutic perspectives. Promoting gut microbial diversity seems to be of major relevance, because for most parameters analysed in this study (*k*_abs_, PLA1 activity, TCA and TβMCA levels and CYP7B1 protein abundance) the values of OMM^12^ mice ranged between those of GF and SPF mice. In humans, gut microbes could be enriched by intake of defined bacterial consortia, possibly containing *A. muciniphila*, via probiotic formulations or prebiotics or diet-based approaches, respectively^58^.

Our work unveils a metabolic handshake between gut microbes and the host to control systemic lipid uptake mediated by biliary PLA1 activity. We showed previously that the gut microbiota promotes hepatic de novo lipid synthesis by providing acetate derived from dietary fibre as precursor^6^. Consequently, we assume that a gut microbiota-induced restriction of intestinal lipid absorption sustains physiological lipid levels. It is not known why systemic uptake rates of dietary lipids vary among healthy individuals^59^. We speculate that interpersonal differences in gut microbiota composition might be a critical factor.

## Methods

### Ethics

All mouse experiments were approved by the General Administration of Bavaria (no. 55.2-1-54-2532-192-2016, no. ROB-55.2-2532.Vet_02-21-124) and by the Swiss Kantonal authorities (license ZH120/19 and ZH058/19; Kantonales Veterinäramt Zürich) and performed according to the legal and ethical requirements.

### Mouse breeding and housing

GF, gnotobiotic (OMM^12^ and OMM^11^ colonized) and SPF C57BL/6JZtm mice (12–14 weeks old; male and female; ~1:1) were obtained from the Central Animal Facility of Hannover Medical School. GF and gnotobiotic mice were bred in plastic film isolators under controlled conditions (20–22 °C, 50–55% humidity, 12 h light–dark cycles). GF and gnotobiotic mice received pelleted 50-kGy gamma-irradiated feed (V1124-927, Ssniff Spezialdiäten GmbH) and autoclaved water ad libitum. SPF mice were housed in individually ventilated cages (XJ Edge) under identical environmental conditions and received the same diet and chlorinated water ad libitum. Routine microbiological monitoring confirmed absence of common murine pathogens (SPF) and contaminants (gnotobiotic)^60,61^. The strains of the OMM^12^ consortium are: *Turicimonas muris* (YL45), *Lactobacillus reuteri* (I49), *Bacteroides caecimuris* (I48), *Clostridium innocuum* (I46), *Blautia coccoides* (YL58), *Muribaculum intestinale* (YL27), *Enterocloster clostridioformis* (YL32), *Flavonifractor plautii* (YL31), *A. muciniphila* (YL44), *Acutalibacter muris* (KB18), *Bifidobacterium animalis* (YL2), *Enterococcus faecalis* (KB1)^10^.

To obtain mice colonized with the EAM strains, wild-type C57BL/6 GF mice (12 weeks old; male) maintained on chow diet and housed at the ETH Phenomics Center (20–22 °C, 50–55% humidity, 12 h light–dark cycles) in sterile flexible film isolators (regularly screened for axenic status) were used. GF mice were exported aseptically to sterile positive-pressure Isocages with sterile food, water and bedding and colonized by oral gavage with pure cultures of *E. coli* O9 HS, *Bacteroides thetaiotaomicron* VPI-5482 and *Eubacterium rectale* ATCC33656 ± *A. muciniphila*^22^. Colonization was confirmed by quantitative PCR (qPCR). The lipid uptake experiments were performed after 1 week of colonization.

*Cyp2c70*^−/−^ mice^30^ and littermate controls (12 weeks old; male) were housed at University Medical Center Hamburg-Eppendorf under standard conditions (22 °C, 12 h light–dark cycle) with ad libitum access to chow and water.

*Cel*^−/−^ (Cel^tm1(KOMP)Vlcg^/Mmucd) and littermate control mice (12 weeks old; male) were bred at the Central Animal Facility of the Hannover Medical School (Germany). The mouse strain, C57BL/6N-Cel^tm1(KOMP)Vlcg^/Mmucd, RRID MMRRC_047050-UCD, was obtained from the Mutant Mouse Resource and Research Center (MMRRC, UC Davis) and was donated to the MMRRC by The KOMP Repository (UC Davis; originating from David Valenzuela, Regeneron Pharmaceuticals)^62^. The breeding of GF *Cel*^−/−^ mice was performed in plastic film isolators, and SPF mice were housed in individually ventilated cages (XJ Edge) under controlled conditions (20–22 °C, 50–55% humidity, 12 h light–dark cycles). Both GF *Cel*^−/−^ and SPF *Cel*^−/−^ mice received pelleted 50-kGy gamma-irradiated feed (V1124-927, Ssniff Spezialdiäten GmbH, Germany) and autoclaved water ad libitum. Routine microbiological monitoring confirmed the absence of common murine pathogens (SPF) and contaminants (gnotobiotic)^60,61^. GF *Cel*^−/−^ and SPF *Cel*^−/−^ mice showed no abnormalities compared with littermate controls.

*Myd88*^−/−^ (Myd88^tm1Aki^)^63^ and littermate control mice (14–16 weeks old; male) were bred and maintained at the Institute of Laboratory Animal Science at the RWTH University Hospital Aachen under SPF conditions (20–22 °C, 50–55% humidity, 12 h light–dark cycles) in individually ventilated cages and ad libitum access to chow diet and water.

### Human bile

Bile was obtained from a female patient (38 years) undergoing cholecystectomy (no malignant disease) at the Department of Surgery (Technical University of Munich). Ethics are approved by the Ethics Committee of the School of Medicine and Health (no. 1926/07; no. 5428/12). Written consent was obtained from the patient.

### Stable isotope labelling of lipid flux

For the lipid flux experiments, mice were starved for 2 h before oral gavage of 100 µl of olive oil containing 10 µmol FA16:0[D5] (hexadecanoic-15,15,16,16,16-d_5_ acid) and 3.33 µmol TG (FA16:0[D31])_3_ (glyceryl tri(hexadecanoate-d_31_)) (CDN isotopes). After 20 min, 1 h, 2 h and 6 h, mice were euthanized by CO_2_ asphyxiation, tissues were collected, snap-frozen in liquid nitrogen and stored at −80 °C. For the experiment investigating the impact of PC supplementation on lipid uptake, SPF mice were starved for 2 h before oral gavage of labelled lipids in olive oil, ± 0.1 µmol of 1-palmitoyl-2-linoleoyl-*sn*-glycero-3-phosphatidylcholine (PC 34:2, Larodan). After 1 h, mice were euthanized by CO_2_ asphyxiation.

### Plasma, bile, tissue and gut content collection

EDTA plasma was collected from the heart by centrifugation (10 min, 1,500*g*, 4 °C). The liver was perfused with saline (0.9% NaCl) to remove blood, and the left lobe was sampled. Bile was collected directly from the gallbladder using an insulin syringe, snap-frozen in liquid nitrogen and stored at −80 °C. Beginning at the base of the stomach, the small intestine was divided into duodenum (first 8 cm), ileum (last 8 cm) and jejunum (middle). The large intestine was collected in one piece. Gut content was collected, and tissues were rinsed with saline to remove residual gavage lipids. Inguinal white adipose tissue (iWAT) and eWAT as well as inguinal brown adipose tissue (iBAT) were collected. All samples were snap-frozen in liquid nitrogen and stored at −80 °C.

Whenever male and female animals were mixed, we balanced groups as well as possible. However, although this was achievable for plasma and tissue samples, it was not possible for bile collection, as mice have limited gallbladder bile volumes, and the amount of bile varied between individuals. No sex-specific differences in BA or lipid levels were observed in plasma, bile and tissues. Males are indicated with ‘•’ and females with ‘+’ in the main figures.

### Gastric flow quantification

Male and female GF, OMM^12^ and SPF C57BL/6JZtm mice (12–14 weeks) were starved for 2 h before receiving an oral gavage of 100 µl of olive oil containing 50 mg ml^−1^ Evans Blue (Sigma-Aldrich). After 20 min, mice were euthanized by CO_2_ asphyxiation, the intestinal system was removed and the distance from the base of the stomach to the blue dye front in the small intestine was measured.

### Physiology-based kinetic modelling

The multi-compartment kinetic model of FA metabolism in the mouse contained 11 compartments (duodenum, jejunum, ileum and colon lumen, corresponding tissues, plasma, liver and fat tissue). One additional compartment (small_intestine_gi) served as reservoir for initial FA abundance. The plasma compartment incorporated processes outside the GIT, liver and fat tissue. Labelled FA administration was modelled as an input to the small_intestine_gi compartment of the initial amount of D. Label propagation through the body was driven by the flow of gastrointestinal material in different GIT sections and lumen:tissue and tissue:serum diffusion and absorption coefficients. Model parameters and equations are provided in Supplementary Data 1. Equations were defined for FA amounts. Parameters for FA16:0[D5] and FA16:0[D31] were assumed identical and fitted simultaneously to both datasets. For parameter fitting, FA concentrations were converted into amounts using estimated compartment volumes (Supplementary Data 1). The model was created using the MatLab 2023a SimBiology Toolbox (MathWorks).

To assess model improvement when parameters were made mouse group-specific, a general model with six parameters was built, which included equations for FA16:0[D5] and FA16:0[D31] from each mouse group, with parameters fitted simultaneously across groups. Next, each of the six parameters was defined as mouse group-specific, and equations were adjusted accordingly. The modified models thus contained eight parameters (one parameter replaced by three group-specific parameters). For each modified model, MSE, AIC and Bayesian information criterion were recorded. The model with the largest AIC decrease was considered the best improvement.

### qPCR of 16S rRNA genes

Genomic DNA (gDNA) was extracted using a phenol-chloroform based protocol^64^. Faecal pellet or intestinal content was weighed and resuspended in 500 µl extraction buffer (200 mM Tris–HCl, 200 mM NaCl, 20 mM EDTA in ddH_2_O, pH 8, autoclaved), 210 µl 20% SDS and 500 µl phenol:chloroform:isoamylalcohol (25:24:1, pH 7.9). In total, 0.1 mm zirconia–silica beads (Roth) were added, and bacteria were lysed with a bead beater (TissueLyser LT, Qiagen) for 4 min at 50 Hz. After centrifugation (14,000*g*, 5 min, room temperature), the aqueous phase was transferred into a new tube, and 500 µl phenol:chloroform:isoamylalcohol (25:24:1, pH 7.9) were added and again spun down. The aqueous phase was mixed with 1 ml 96% ethanol and 50 µl of 3 M sodium acetate. After centrifugation (30 min, 14,000*g*, 4 °C), the supernatant was discarded, and the gDNA pellet was washed with 500 µl ice-cold 70% ethanol and centrifuged (14,000*g*, 4 °C, 15 min). The pellet was resuspended in 150 µl Tris–HCl, pH 8. The resulting gDNA was purified using the NucleoSpin gDNA clean-up kit (Macherey-Nagel) and stored at −20 °C.

For the qPCR, as a template, 2.5 µl of gDNA (2 ng µl^−1^) were used^10^. Strain-specific 16S rRNA primers and hydrolysis probes were used for amplification (LightCycler 96, Roche). The FastStart Essential DNA Probes Master (Roche) was used for the reactions. Standard curves using linearized plasmids containing the 16S rRNA gene sequence of the individual strains were used for the absolute quantification of 16S rRNA gene copy numbers of individual strains. The readout of 16S rRNA copies per 5 ng gDNA was normalized to the individual sample weight to determine 16S rRNA copies per gram of intestinal content (absolute abundance).

### qPCR of mammalian genes

Frozen tissue samples were ground under liquid nitrogen using a mortar and pestle, and total RNA was extracted using QIAshredder columns and the RNEasy Mini Kit (both Qiagen). The purity and integrity of the RNA were assessed using the Agilent 2100 Bioanalyzer (Agilent Technologies). In total, 1 µg RNA was reverse transcribed using the Reverse Transcription System from Promega. RT-qPCR analysis was performed using the LightCycler 480 (Roche). Quantification relative to GF mouse tissue was carried out using the ${2}^{-\Delta\Delta{\rm{C}}{\rm{t}}}$ method^65^, *β-actin* and *GAPDH* served as reference genes. The primers used were

Mouse:

*Cd36*: fw-3′-GGACATTGAGATTCTTTTCCTCTG-5′/rev-3′-GCAAAGGCATTGGCTGGAAGAAC-5′

*Dhhc4*: fw-3′-ACACACCTCAGCCTGGCTACTA-5′/rev-3′-ATGGCGGCTGATGTGCTACTGC-5′

*Dhhc5*: fw-3′-CTCCTTGGATGTGGCAAAGG-5′/rev-3′-TCCATGCTGGAGGAAGGTAAC-5′

*Fatp4*: fw-3′-GCACAGCAGGTATTATCGTATGG-5′/rev-3′-TGCTGAGTGGTAGAGGGGGA-5′; *Cyp7a1*: fw-3′-ACCTGCAAACTGATGGGGAAATAT-5′/rev-3′-TTGGGTCTATGCTTCTGTGTCCAAA-5′

*Cyp7b1*: fw-3′-TTCAGGAAAGGCAAGATCTGCTGA-5/rev-3′-CCCAGAGAAAGCCAAGATGATG-5′

*Cyp8b1*: fw-3′-CTGGGTCCTCTTATTCCTGCTG-5′/rev-3′-TCAGGGCACTGGGAGTGAAA-5′

*Cyp2c70*: fw-3′-CGGATTCATCGTTACTCAGTAG-5′/rev-3′-TTCTTTCCCATCCCCATAGACCT-5′

*Cyp27a1*: fw-3′-TGTGCTGCACTTGCCCGACC-5′/rev-3′-GGAGGTTGTCCACATTGG-5′

*β-actin*: fw-3′-GTGCGTGACATCAAAGAG-5′/rev-3′-GCCACAGGATTCCATACC-5′.

Human (HepG2):

*Myd88*: fw-3′-GAGGCTGAGAAGCCTTTACAGG-5/rev-3′-GCAGATGAAGGCATCGAAACGC-5′

*Cyp7b1*: fw-3′-CACCAGAGAACAATTGGACAGCC-5/rev-3′-GCTACCAAGTCTCCCTTTCGCA-5′

*Gapdh*: fw-3′-GGCCTCCAAGGAGTAAGACC-5/rev-3′-AGGGGAGATTCAGTGTGGTG-5′

### Bile and liver proteomics

C57BL/6 mice (male, 12–14 weeks) were fasted for 2 h and euthanized, and bile samples were collected by puncture of the gallbladder using an insulin syringe. Samples were frozen in liquid nitrogen and stored at −80 °C. Bile from six mice was pooled. Human bile was obtained from the Department of Surgery, Technical University of Munich as described under the ‘Human bile’ section and stored at −80 °C.

Bile proteins were purified and digested on-column using the S-Trap Micro MS Sample Prep Kit (Protifi) according to the manufacturer’s protocol, whereas mouse livers were lysed via beads-beating in lysis buffer containing 8 M urea, 40 mM Tris–HCl (pH 8.5) and 1× EDTA-free complete protease inhibitor tablet and further processed^66^. See the Supplementary Note for more details.

### Search for common regulators of *Cyp7b1*

Differentially abundant liver proteins (abs(log_2_(FC)) ≥ abs(log_2_(1.5)), FDR < 0.05) in OMM^12^ and SPF groups versus GF (*n* = 191) were compared with differentially abundant genes across publicly available studies from Expression Atlas (https://www.ebi.ac.uk/gxa/home). Filtering for ‘*Mus musculus*’ and ‘genotype or phenotype’ yielded 216 RNA sequencing and 528 Affymetrix Microarray datasets. FCs between different study groups were downloaded and combined in one table. The comparison between the set of 191 differentially abundant proteins and differentially abundant genes in each study (abs(log_2_(FC)) ≥ abs(log_2_(1.5))) used Fisher’s exact test (fishertest, MatLab) to calculate *P* values and odds ratios. *P* values were adjusted for multiple hypothesis testing (Benjamini–Hochberg; mafdr function in MatLab 2019b (‘bhfdr’, 1)).

### Phospholipase activity assays

Mice were fasted for 2 h and euthanized, and bile samples were collected by direct puncture of the gallbladder using an insulin syringe. Samples were immediately frozen in liquid nitrogen and stored at −80 °C. As a negative control, proteins were denatured by heat (95 °C, 15 min, 700 rpm, 1% of SDS), then centrifuged (16,100*g*, 4 °C, 5 min). For determining the effect of BA concentration on PLA activity, 1 mM and 5 mM BAs where dissolved in the bile samples beforehand. The phospholipase substrate PC15:0/18:1[D7] (1 mg ml^−1^ in chloroform, Avanti Polar Lipids) was evaporated in reaction tubes, and tubes were prewarmed at 37 °C. Baseline samples before the addition of bile to the substrate were drawn, diluted 1:100 in ddH_2_O and frozen in liquid nitrogen. To start the reactions, bile was added and vortexed for 30 s. The final substrate concentration in the bile was set to 10 nmol µl^−1^. Samples were incubated at 37 °C and 700 rpm. At the respective time points, samples were drawn, diluted 1:100 in ddH_2_O and frozen in liquid nitrogen to quench the reactions.

To determine microbial PLA activity, the OMM^12^ strains (in 1:1 ratios) cultivated in anaerobic *Akkermansia* medium at 37 °C were added to evaporated PC15:0/18:1[D7] (10 nmol µl^−1^) and incubated for 24 h. As positive control, PLA1 from *Aspergillus oryzae* (2.75 LU ml^−1^; Sigma-Aldrich) was added.

### BSH activity assays

Fresh caecal contents of GF (*n* = 1), OMM^12^ (*n* = 6) and SPF (*n* = 5) mice were diluted 1:10 (w/v) in anoxic, sterile PBS containing 20% (v/v) glycerol and 0.05% (w/v) L-cystein. Samples were snap-frozen and stored at −75 °C. Anaerobic sterile brain hear infusion broth (Oxoid, CM1135) containing 0.05% (w/v) L-cystein, 0.02% dithiothreitol (w/v), 10 μM sodium taurocholate (BRP, European Pharmacopoeia Reference Standard, S0900000) and 10 μM TβMCA sodium salt (Avanti, 700244 P) was prepared. In a 2-ml deep-well plate, 1.710 ml of brain hear infusion broth containing a total of 20 μM bile salts was mixed with 90-μl caecal samples or PBS and incubated at 37 °C. Incubation and preparation were conducted in an anaerobic chamber (89.3% N_2_, 6% CO_2_, 4.7% H_2_). Samples (100 μl) were taken before the start of incubation and 30 and 90 min after incubation. The samples were centrifuged (12,000*g*, 10 min, 4 °C), the supernatant transferred into new tubes, snap-frozen and used for further analysis.

### Total FA analysis

For liver, lung and intestinal tissues, 1 mg of tissue was used for the FA derivatization. For fat tissues, 0.2 mg of tissue was used. For plasma analysis, 10 µl was used. Bile was diluted 1:100 in ddH_2_O, and 20 µl of the dilutions was used. For gut content measurements, 125 µl of the gut contents in 70% isopropanol ( = 0.2 mg dry weight equivalent) was used. Total FA were quantified as methyl esters by gas chromatography–mass spectrometry^67^. Unlabelled FA species were quantified by single-ion monitoring (saturated, *m*/*z* 74; mono-unsaturated, *m*/*z* 55; di-unsaturated, *m*/*z* 67; polyunsaturated, *m*/*z* 79). Isotopically labelled FA species were quantified by single-ion monitoring of their respective molecular ions using the calibration curves of the unlabelled species. The internal standard was non-naturally occurring FA 21:0 iso.

### Lipidomics

Samples were subjected to lipid extraction in presence of internal standards^68^. The following sample amounts were used: 10 µl plasma, tissue homogenates representing a wet weight of 2 mg and bile 100 nl (or 500 nl for phospholipase activity assays). Dried chloroform residues were dissolved in either in 10 mM ammonium acetate in methanol/chloroform (3:1, v/v) (for low mass resolution tandem mass spectrometry) or chloroform/methanol/2-propanol (1:2:4 v/v/v) with 7.5 mM ammonium formate (for high-resolution mass spectrometry).

The quantification of lipids was performed by direct flow injection analysis (FIA) using a triple quadrupole mass spectrometer (FIA–MS/MS; QQQ triple quadrupole) and a hybrid quadrupole-Orbitrap mass spectrometer (high mass resolution). FIA–MS/MS was carried out in positive ion mode using the analytical set-up and strategy described previously^69^. The fragment ions of *m*/*z* 184 and *m*/*z* 264 were used for LPC^69^ and sphingosine-based ceramides (Cer)/hexosylceramides (HexCer)^70^, respectively. The following neutral losses (in Daltons) were applied: phosphatidylethanolamine (PE) 141; phosphatidylserine (PS) 185; phosphatidylglycerol (PG) 189; phosphatidylinositol (PI) 277 (ref. ^71^). PE-based plasmalogens (PE P) were analysed according to the principles described by Zemski-Berry^72^. TG, diglycerides and cholesteryl ester were recorded in positive ion mode^68^. PC and sphingomyelin were measured in negative ion mode. Multiplexed acquisition was used for the [M + NH_4_]^+^ of free cholesterol^73^. The quantification was performed by multiplication of the spiked internal standard amount with analyte-to-internal standard ratio^68^. Labelled complex lipids were analysed at the respective accurate *m*/*z* and quantified using the respective internal standard. Lipid species were annotated according to the latest proposal for shorthand notation of lipid structures that are derived from mass spectrometry^74^. For QQQ analysis, glycerophospholipid species annotation was based on the assumption of even numbered carbon chains only. Cer and HexCer species annotation is based on the assumption that a sphingoid base with two hydroxyl groups is present.

### BA and oxysterol analyses

BA were quantified by liquid chromatography–tandem mass spectrometry^75^. Bile samples corresponding to 100 nl and liver samples corresponding to 2 mg wet weight were subjected to analysis; 27-HC was quantified using gas chromatography–tandem mass spectrometry^76^.

### Cell culture experiments

#### RNA interference in HepG2 cells

The siRNA-mediated gene knockdown was performed by forward transfection using Dicer-substrate siRNA (DsiRNA) or a non-targeting negative control DsiRNA (Integrated DNA Technologies) according to the manufacturer’s instructions with minor modifications (Lipofectamine RNAiMAX; Invitrogen, protocol pub. no. MAN0007825 Rev.1). HepG2 cells (HB-8065 ATCC), authenticated by STR profiling (ExPasy Cellosaurus), were seeded in DMEM (D5796, Sigma-Aldrich) supplemented with 10% FBS Superior (Biochrom), penicillin–streptomycin (1:250) and gentamicin (1:250). Cells were transfected at ~70% confluency using a mixture of Opti-MEM Reduced Serum Medium (Gibco), Lipofectamine RNAiMAX (3 μl ml^−1^) and DsiRNA (15 nM). Antibiotics were omitted during the first 24 h of transfection. Cells were incubated for 72 h before downstream analyses.

DsiRNA sequences: siMyd88I (5′-CAUCACUGUCUGCGACUACACCAAC-3′;3′-AAGUAGUGACAGACGCUGAUGUGGUUG-5′); siMyd88II (5′-GUUGUCUCUGAUGAUUACCUGCAGA-3′;3′-ACCAACAGAGACUACUAAUGGACGUCU-5′); non-targeting siControl (5′-CGUUAAUCGCGUAUACGCGUAT-3′; 3′-AUACGCGUAUUAUACGCGAUUAACGAC-5′).

#### Immunoblotting of Myd88

For immunoblotting, a Minigel-Tank and Blot-Module system was used (Thermo Fisher Scientific, NW2000). Proteins were separated in pre-cast gradient Bis-Tris gels (NuPAGE 4–12%; Thermo Fisher Scientific, NP0336) according to the manufacturer’s recommendations. In total, 30 µg protein per lane and 3 µg ladder were used (PageRuler Plus prestained; Thermo Fisher Scientific, 26616); electrophoresis was run at 120 V for 80 min. The gel containing separated proteins was assembled between presoaked filter papers (Thermo Fisher Scientific, 84784) and sponges according to the manufacturer’s instructions and transferred onto a nitrocellulose membrane (Thermo Fisher Scientific, 88018) at 10 V for 60 min. Membranes were stained with Ponceau S to verify equal protein loading and efficient transfer. They were blocked with 5% BSA diluted in Tris-buffered saline with Tween 20 (0.1% Tween 20) for 1 hour at room temperature. Primary antibodies were incubated overnight at 4 °C, secondary antibodies for 1 hour at room temperature. Between incubations, membranes were washed for 30 minutes conducting Tris-buffered saline with Tween 20 changes every 5 min. Before signal detection (Bio-Rad ChemiDoc Imaging System), membranes were incubated with chemiluminescent horseradish peroxidase (HRP) substrate (Immobilon Classico/Crescendo Western HRP Substrate; Millipore or SuperSignal West Femto/Atto substrate; Thermo Scientific) according to the manufacturer’s instructions. Used antibodies included: HSP90 (4877S Cell Signaling, 1:1,000), MYD88 (4283S Cell Signaling, 1:500) and anti-rabbit HRP secondary antibody (7074P2 Cell Signaling, 1:4,000).

### Statistics

FA and lipidomic data were analysed according to established principles^6^. For lipid time profile comparison, the AUC was calculated as the sum of mean values across timepoints. Significance of differences between mouse group time profiles was assessed with repeated measured analysis of variance (ANOVA) analysis (fitrm function in MatLab 2019b). *P* values were adjusted for multiple hypothesis testing (Benjamini–Hochberg; mafdr function in MatLab 2019b (‘bhfdr’, 1)).

### Reporting summary

Further information on research design is available in the Nature Portfolio Reporting Summary linked to this article.

## Supplementary information

Supplementary Information Supplementary Note.

Reporting summary

Peer Review File

Supplementary Data 1 Model parameters and equations of physiology-based kinetic modelling.

Supplementary Data 2 Bile proteome (mouse-GF, OMM^12^, SPF; human) and liver proteome (mouse-GF, OMM^12^, SPF).

## Source data

Source Data Fig. 1 Statistical source data.

Source Data Fig. 2 Statistical source data.

Source Data Fig. 3 Statistical source data.

Source Data Fig. 4 Statistical source data.

Source Data Fig. 5 Statistical source data.

Source Data Fig. 6a–i Statistical source data.

Source Data Fig. 6j Unprocessed western blots.

Source Data Extended Data Fig. 1 Statistical source data.

Source Data Extended Data Fig. 2 Statistical source data.

Source Data Extended Data Fig. 3 Statistical source data.

Source Data Extended Data Fig. 4 Statistical source data.

Source Data Extended Data Fig. 5 Statistical source data.

Source Data Extended Data Fig. 6 Statistical source data.

Source Data Extended Data Fig. 7 Statistical source data.

Source Data Extended Data Fig. 9 Statistical source data.

Source Data Extended Data Fig. 10 Statistical source data.

## Data Availability

Mass spectrometry bile and liver proteomics data have been deposited in the ProteomeXchange Consortium via the PRIDE partner repository with the dataset identifier PXD033894 and PXD060110. The publicly available RNAseq and Affymetrix Microarray data downloaded via Expression Atlas at https://www.ebi.ac.uk/gxa/home are available via Zenodo at 10.5281/zenodo.15092709 (ref. ^77^). Lipidomics data, model parameters and equations used for physiology-based kinetic multi-compartment modelling are reported in Supplementary Data 1. Source data are provided with this paper.

## Code availability

The code for the physiology-based kinetic modelling and liver proteomics analysis is available via GitHub at https://github.com/mszimmermann/Microbiome_effect_on_lipid_metabolism.

## References

[1] Hooper, L. et al. Effects of total fat intake on body weight. *Cochrane Database Syst. Rev.* **8**, 1465–1858 (2015). 10.1002/14651858.CD011834 PMC10403157 26250104

[2] Hussain, M. M. Intestinal lipid absorption and lipoprotein formation. *Curr. Opin. Lipidol.* **25**, 200–206 (2014). 24751933 10.1097/MOL.0000000000000084 PMC4265799

[3] Jumpertz, R. et al. Energy-balance studies reveal associations between gut microbes, caloric load, and nutrient absorption in humans. *Am. J. Clin. Nutr.* **94**, 58–65 (2011). 21543530 10.3945/ajcn.110.010132 PMC3127503

[4] Sender, R., Fuchs, S. & Milo, R. Revised estimates for the number of human and bacteria cells in the body. *PLoS Biol.* **14**, e1002533 (2016). 27541692 10.1371/journal.pbio.1002533 PMC4991899

[5] Fan, Y. & Pedersen, O. Gut microbiota in human metabolic health and disease. *Nat. Rev. Microbiol.* **19**, 55–71 (2021). 32887946 10.1038/s41579-020-0433-9

[6] Kindt, A. et al. The gut microbiota promotes hepatic fatty acid desaturation and elongation in mice. *Nat. Commun.* **9**, 3760 (2018). 30218046 10.1038/s41467-018-05767-4 PMC6138742

[7] Fleishman, J. S. & Kumar, S. Bile acid metabolism and signaling in health and disease: molecular mechanisms and therapeutic targets. *Signal. Transduct. Target. Ther.* **9**, 97 (2024). 38664391 10.1038/s41392-024-01811-6 PMC11045871

[8] Araujo, J. R. et al. Fermentation products of commensal bacteria alter enterocyte lipid metabolism. *Cell Host Microbe* **27**, 358–375 (2020). 32101704 10.1016/j.chom.2020.01.028

[9] Martinez-Guryn, K. et al. Small intestine microbiota regulate host digestive and absorptive adaptive responses to dietary lipids. *Cell Host Microbe* **23**, 458–469 (2018). 29649441 10.1016/j.chom.2018.03.011 PMC5912695

[10] Brugiroux, S. et al. Genome-guided design of a defined mouse microbiota that confers colonization resistance against *Salmonella enterica* serovar *Typhimurium*. *Nat. Microbiol.* **2**, 16215 (2016). 27869789 10.1038/nmicrobiol.2016.215

[11] Hoces, D. et al. Metabolic reconstitution of germ-free mice by a gnotobiotic microbiota varies over the circadian cycle. *PLoS Biol.* **20**, e3001743 (2022). 36126044 10.1371/journal.pbio.3001743 PMC9488797

[12] Zimmermann, M. et al. Separating host and microbiome contributions to drug pharmacokinetics and toxicity. *Science* **363**, eaat9931 (2019). 30733391 10.1126/science.aat9931 PMC6533120

[13] Wit, M. et al. When fat meets the gut-focus on intestinal lipid handling in metabolic health and disease. *EMBO Mol. Med.* **14**, e14742 (2022). 35437952 10.15252/emmm.202114742 PMC9081902

[14] Wang, Y. et al. The intestinal microbiota regulates body composition through NFIL3 and the circadian clock. *Science* **357**, 912–916 (2017). 28860383 10.1126/science.aan0677 PMC5702268

[15] Wang, J. et al. DHHC4 and DHHC5 facilitate fatty acid uptake by palmitoylating and targeting CD36 to the plasma membrane. *Cell Rep.* **26**, 209–221 (2019). 30605677 10.1016/j.celrep.2018.12.022

[16] Hao, J. W. et al. CD36 facilitates fatty acid uptake by dynamic palmitoylation-regulated endocytosis. *Nat. Commun.* **11**, 4765 (2020). 32958780 10.1038/s41467-020-18565-8 PMC7505845

[17] Li, Q. et al. Extracellular vesicles from *Limosilactobacillus johnsonii* enhance milk fat synthesis by inducing CD36 dynamic palmitoylation and activating PPARγ signalling. *J. Extracell. Vesicles* **14**, e70143 (2025). 40767021 10.1002/jev2.70143 PMC12326193

[18] Wang, H. et al. Inhibition of fatty acid uptake by TGR5 prevents diabetic cardiomyopathy. *Nat. Metab.* **6**, 1161–1177 (2024). 38698281 10.1038/s42255-024-01036-5 PMC11199146

[19] Zhai, H. et al. Takeda G protein-coupled receptor 5-mechanistic target of rapamycin complex 1 signaling contributes to the increment of glucagon-like peptide-1 production after roux-en-Y gastric bypass. *eBioMedicine* **32**, 201–214 (2018). 29859856 10.1016/j.ebiom.2018.05.026 PMC6020750

[20] Everard, A. et al. Cross-talk between *Akkermansia muciniphila* and intestinal epithelium controls diet-induced obesity. *Proc. Natl Acad. Sci. USA* **110**, 9066–9071 (2013). 23671105 10.1073/pnas.1219451110 PMC3670398

[21] Depommier, C. et al. Supplementation with *Akkermansia muciniphila* in overweight and obese human volunteers: a proof-of-concept exploratory study. *Nat. Med.* **25**, 1096–1103 (2019). 31263284 10.1038/s41591-019-0495-2 PMC6699990

[22] Lan, J. et al. Non-invasive monitoring of microbiota and host metabolism using secondary electrospray ionization-mass spectrometry. *Cell Rep. Methods* **3**, 100539 (2023). 37671025 10.1016/j.crmeth.2023.100539 PMC10475793

[23] Tso, P. et al. Role of biliary phosphatidylcholine in the absorption and transport of dietary triolein in the rat. *Gastroenterology* **80**, 60–65 (1981). 6893826

[24] Mansbach, C. M. 2nd & Gorelick, F. Development and physiological regulation of intestinal lipid absorption. II. Dietary lipid absorption, complex lipid synthesis, and the intracellular packaging and secretion of chylomicrons. *Am. J. Physiol. Gastrointest. Liver Physiol.* **293**, G645–G650 (2007). 17627968 10.1152/ajpgi.00299.2007

[25] Kennelly, J. P. et al. Intestinal de novo phosphatidylcholine synthesis is required for dietary lipid absorption and metabolic homeostasis. *J. Lipid Res.* **59**, 1695–1708 (2018). 30007917 10.1194/jlr.M087056 PMC6121921

[26] van Helvoort, A. et al. MDR1 P-glycoprotein is a lipid translocase of broad specificity, while MDR3 P-glycoprotein specifically translocates phosphatidylcholine. *Cell* **87**, 507–517 (1996). 8898203 10.1016/s0092-8674(00)81370-7

[27] Hui, D. Y. & Howles, P. N. Carboxyl ester lipase: structure-function relationship and physiological role in lipoprotein metabolism and atherosclerosis. *J. Lipid Res.* **43**, 2017–2030 (2002). 12454261 10.1194/jlr.r200013-jlr200

[28] Fontbonne, H. et al. Human bile salt-dependent lipase efficiency on medium-chain acyl-containing substrates: control by sodium taurocholate. *J. Biochem.* **149**, 145–151 (2011). 21081507 10.1093/jb/mvq132

[29] Wang, X. et al. The crystal structure of bovine bile salt activated lipase: insights into the bile salt activation mechanism. *Structure* **5**, 1209–1218 (1997). 9331420 10.1016/s0969-2126(97)00271-2

[30] de Boer, J. F. et al. Cholangiopathy and biliary fibrosis in Cyp2c70-deficient mice are fully reversed by ursodeoxycholic acid. *Cell. Mol. Gastroenterol. Hepatol.* **11**, 1045–1069 (2021). 33309945 10.1016/j.jcmgh.2020.12.004 PMC7898074

[31] Moreno, P. et al. Expression Atlas update: gene and protein expression in multiple species. *Nucleic Acids Res.* **50**, D129–D140 (2022). 34850121 10.1093/nar/gkab1030 PMC8728300

[32] Fitzgerald, K. A. & Kagan, J. C. Toll-like receptors and the control of immunity. *Cell* **180**, 1044–1066 (2020). 32164908 10.1016/j.cell.2020.02.041 PMC9358771

[33] Duparc, T. et al. Hepatocyte MyD88 affects bile acids, gut microbiota and metabolome contributing to regulate glucose and lipid metabolism. *Gut* **66**, 620–632 (2017). 27196572 10.1136/gutjnl-2015-310904 PMC5529962

[34] Dahl, K. et al. Oral semaglutide improves postprandial glucose and lipid metabolism, and delays gastric emptying, in subjects with type 2 diabetes. *Diabetes Obes. Metab.* **23**, 1594–1603 (2021). 33710717 10.1111/dom.14373 PMC8251575

[35] Qin, X. et al. GLP-1 reduces intestinal lymph flow, triglyceride absorption, and apolipoprotein production in rats. *Am. J. Physiol. Gastrointest. Liver Physiol.* **288**, G943–G949 (2005). 15677555 10.1152/ajpgi.00303.2004

[36] Mansouri, S. et al. MPYS modulates fatty acid metabolism and immune tolerance at homeostasis independent of type I IFNs. *J. Immunol.* **209**, 2114–2132 (2022). 36261171 10.4049/jimmunol.2200158 PMC9679991

[37] Gao, Y. et al. T cell cholesterol transport links intestinal immune responses to dietary lipid absorption. *Science* **390**, eadt4169 (2025). 41066556 10.1126/science.adt4169 PMC13034976

[38] Burkhardt, R. & Ecker, J. A tripartite alliance for dietary fat absorption. *Science* **390**, 128–129 (2025). 41066588 10.1126/science.aeb5717

[39] Kashyap, P. C. et al. Complex interactions among diet, gastrointestinal transit, and gut microbiota in humanized mice. *Gastroenterology* **144**, 967–977 (2013). 23380084 10.1053/j.gastro.2013.01.047 PMC3890323

[40] Frith, M. E. et al. Microbiota-dependent early-life programming of gastrointestinal motility. *iScience* **27**, 110895 (2024). 39351201 10.1016/j.isci.2024.110895 PMC11440258

[41] Higashiyama, H. et al. Anatomy of the murine hepatobiliary system: a whole-organ-level analysis using a transparency method. *Anat. Rec.* **299**, 161–172 (2016). 10.1002/ar.23287 26559382

[42] Combes, B. et al. Biliary bile acids in primary biliary cirrhosis: effect of ursodeoxycholic acid. *Hepatology* **29**, 1649–1654 (1999). 10347103 10.1002/hep.510290618 PMC4004074

[43] Li, R. et al. Low production of 12α-hydroxylated bile acids prevents hepatic steatosis in Cyp2c70^−/−^ mice by reducing fat absorption. *J. Lipid Res.* **62**, 100134 (2021). 34626589 10.1016/j.jlr.2021.100134 PMC8596750

[44] Evangelakos, I. et al. Cold-induced lipoprotein clearance in Cyp7b1-deficient mice. *Front. Cell Dev. Biol.* **10**, 836741 (2022). 35478959 10.3389/fcell.2022.836741 PMC9038073

[45] Bertaggia, E. et al. Cyp8b1 ablation prevents Western diet-induced weight gain and hepatic steatosis because of impaired fat absorption. *Am. J. Physiol. Endocrinol. Metab.* **313**, 121–133 (2017). 10.1152/ajpendo.00409.2016 PMC5582885 28377401

[46] Oteng, A. B. et al. Cyp2c-deficiency depletes muricholic acids and protects against high-fat diet-induced obesity in male mice but promotes liver damage. *Mol. Metab.* **53**, 101326 (2021). 34438105 10.1016/j.molmet.2021.101326 PMC8449133

[47] Phelps, T. et al. The influence of biological sex and sex hormones on bile acid synthesis and cholesterol homeostasis. *Biol. Sex Differ.* **10**, 52 (2019). 31775872 10.1186/s13293-019-0265-3 PMC6880483

[48] Sayin, S. I. et al. Gut microbiota regulates bile acid metabolism by reducing the levels of tauro-β-muricholic acid, a naturally occurring FXR antagonist. *Cell Metab.* **17**, 225–235 (2013). 23395169 10.1016/j.cmet.2013.01.003

[49] Sun, L., Cai, J. & Gonzalez, F. J. The role of farnesoid X receptor in metabolic diseases, and gastrointestinal and liver cancer. *Nat. Rev. Gastroenterol. Hepatol.* **18**, 335–347 (2021). 33568795 10.1038/s41575-020-00404-2

[50] Liu, J. et al. Diindoles produced from commensal microbiota metabolites function as endogenous CAR/Nr1i3 ligands. *Nat. Commun.* **15**, 2563 (2024). 38519460 10.1038/s41467-024-46559-3 PMC10960024

[51] Lickteig, A. J. et al. Activation of constitutive androstane receptor (CAR) in mice results in maintained biliary excretion of bile acids despite a marked decrease of bile acids in liver. *Toxicol. Sci.* **151**, 403–418 (2016). 26984780 10.1093/toxsci/kfw054 PMC4880140

[52] Linthorst, J. M., Bennett Clark, S. & Holt, P. R. Triglyceride emulsification by amphipaths present in the intestinal lumen during digestion of fat. *J. Colloid Interface Sci.* **60**, 1–10 (1977).

[53] Brownlee, I. A. et al. Physiological parameters governing the action of pancreatic lipase. *Nutr. Res. Rev.* **23**, 146–154 (2010). 20193096 10.1017/S0954422410000028

[54] Greten, H. et al. The effect of polyunsaturated phosphatidylcholine on plasma lipids and fecal sterol excretion. *Atherosclerosis* **36**, 81–88 (1980). 7387779 10.1016/0021-9150(80)90201-4

[55] Kesaniemi, Y. A. & Grundy, S. M. Effects of dietary polyenylphosphatidylcholine on metabolism of cholesterol and triglycerides in hypertriglyceridemic patients. *Am. J. Clin. Nutr.* **43**, 98–107 (1986). 3942098 10.1093/ajcn/43.1.98

[56] Beil, F. U. & Grundy, S. M. Studies on plasma lipoproteins during absorption of exogenous lecithin in man. *J. Lipid Res.* **21**, 525–536 (1980). 7400685

[57] Bardou, M., Barkun, A. N. & Martel, M. Obesity and colorectal cancer. *Gut* **62**, 933–947 (2013). 23481261 10.1136/gutjnl-2013-304701

[58] Gagliardi, A. et al. Rebuilding the gut microbiota ecosystem. *Int. J. Environ. Res. Public Health* **15**, 1679 (2018). 30087270 10.3390/ijerph15081679 PMC6121872

[59] Karrasch, T. et al. Short-term regulation of visfatin release in vivo by oral lipid ingestion and in vitro by fatty acid stimulation. *Exp. Clin. Endocrinol. Diabetes* **122**, 126–134 (2014). 24554513 10.1055/s-0033-1363262

[60] Mahler Convenor, M. et al. FELASA recommendations for the health monitoring of mouse, rat, hamster, guinea pig and rabbit colonies in breeding and experimental units. *Lab. Anim.* **48**, 178–192 (2014). 24496575 10.1177/0023677213516312

[61] Basic, M. et al. Monitoring and contamination incidence of gnotobiotic experiments performed in microisolator cages. *Int. J. Med. Microbiol.* **311**, 151482 (2021). 33636479 10.1016/j.ijmm.2021.151482

[62] Valenzuela, D. M. et al. High-throughput engineering of the mouse genome coupled with high-resolution expression analysis. *Nat. Biotechnol.* **21**, 652–659 (2003). 12730667 10.1038/nbt822

[63] Adachi, O. et al. Targeted disruption of the MyD88 gene results in loss of IL-1- and IL-18-mediated function. *Immunity* **9**, 143–150 (1998). 9697844 10.1016/s1074-7613(00)80596-8

[64] Herp, S. et al. *Mucispirillum schaedleri* antagonizes salmonella virulence to protect mice against colitis. *Cell Host Microbe* **25**, 681–694 (2019). 31006637 10.1016/j.chom.2019.03.004

[65] Langmann, T., Mauerer, R. & Schmitz, G. Human ATP-binding cassette transporter TaqMan low-density array: analysis of macrophage differentiation and foam cell formation. *Clin. Chem.* **52**, 310–313 (2006). 16449213 10.1373/clinchem.2005.059774

[66] Giansanti, P. et al. Mass spectrometry-based draft of the mouse proteome. *Nat. Methods* **19**, 803–811 (2022). 35710609 10.1038/s41592-022-01526-y PMC7613032

[67] Ecker, J. et al. A rapid GC–MS method for quantification of positional and geometric isomers of fatty acid methyl esters. *J. Chromatogr. B* **897**, 98–104 (2012). 10.1016/j.jchromb.2012.04.015 22542399

[68] Horing, M. et al. Accurate quantification of lipid species affected by isobaric overlap in Fourier-transform mass spectrometry. *J. Lipid Res.* **62**, 100050 (2021). 33600775 10.1016/j.jlr.2021.100050 PMC8010702

[69] Liebisch, G. et al. High-throughput quantification of phosphatidylcholine and sphingomyelin by electrospray ionization tandem mass spectrometry coupled with isotope correction algorithm. *Biochim. Biophys. Acta* **1686**, 108–117 (2004). 15522827 10.1016/j.bbalip.2004.09.003

[70] Liebisch, G. et al. Quantitative measurement of different ceramide species from crude cellular extracts by electrospray ionization tandem mass spectrometry (ESI–MS/MS). *J. Lipid Res.* **40**, 1539–1546 (1999). 10428992

[71] Matyash, V. et al. Lipid extraction by methyl-*tert*-butyl ether for high-throughput lipidomics. *J. Lipid Res.* **49**, 1137–1146 (2008). 18281723 10.1194/jlr.D700041-JLR200 PMC2311442

[72] Zemski Berry, K. A. & Murphy, R. C. Electrospray ionization tandem mass spectrometry of glycerophosphoethanolamine plasmalogen phospholipids. *J. Am. Soc. Mass Spectrom.* **15**, 1499–1508 (2004). 15465363 10.1016/j.jasms.2004.07.009

[73] Horing, M. et al. Quantification of cholesterol and cholesteryl ester by direct flow injection high-resolution fourier transform mass spectrometry utilizing species-specific response factors. *Anal. Chem.* **91**, 3459–3466 (2019). 30707563 10.1021/acs.analchem.8b05013

[74] Liebisch, G. et al. Update on LIPID MAPS classification, nomenclature, and shorthand notation for MS-derived lipid structures. *J. Lipid Res.* **61**, 1539–1555 (2020). 33037133 10.1194/jlr.S120001025 PMC7707175

[75] Hermeling, S. et al. Rapid quantification of murine bile acids using liquid chromatography-tandem mass spectrometry. *Anal. Bioanal. Chem.* **417**, 687–696 (2024). 39621039 10.1007/s00216-024-05668-0 PMC11772536

[76] Matysik, S., Klunemann, H. H. & Schmitz, G. Gas chromatography-tandem mass spectrometry method for the simultaneous determination of oxysterols, plant sterols, and cholesterol precursors. *Clin. Chem.* **58**, 1557–1564 (2012). 22997279 10.1373/clinchem.2012.189605

[77] Zimmermann-Kogadeeva, M., Ecker, J. & Plagge, J. Microbiome effect on lipid metabolism. *Zenodo*10.5281/zenodo.15092709 (2025).

