# Supplementary material for: Gut microbiota restricts intestinal lipid uptake via modulation of bile phosphatidylcholine metabolism in mice
Source: Nat Microbiol. 2026 Jul 29;11(8):2349–64. doi: 10.1038/s41564-026-02434-z (PMC13423828; doi:10.1038/s41564-026-02434-z)
Supplement: Supplementary file 1 — Supplementary Note. [file 41564_2026_2434_MOESM1_ESM.pdf]

nature microbiology

Supplementary information https://doi.org/10.1038/s41564-026-02434-z

Gut microbiota restricts intestinal lipid uptake via modulation of bile phosphatidylcholine metabolism in mice

In the format provided by the authors and unedited

Supplementary Note

Proteomics of bile and liver

Bile samples were processed into protein LoBind tubes (Eppendorf) to reduce protein loss during the purification/digestion. In brief, 11.5 μL of human bile or 30 μL of mouse bile were mixed 1:1 with 2 × lysis buffer (10 % SDS, 100 mM TEAB, pH 8.5) and vortexed. Reductant (120 mM TCEP) was added to the bile/lysis buffer mixture at a ratio of 1:23 and the samples were incubated at 55 °C for 15 min at 300 rpm. Alkylation of disulfides was performed by adding the alkylator (500 mM MMTS in isopropanol) at a ratio of 1:23 based on the initial sample/lysis buffer volume and incubating at RT for 10 min and 300 rpm. Acidifier (27.5 % aqueous phosphoric acid) was added at a 1:10 ratio to the sample and vortexed. 6 × the volume of binding/wash buffer (100 mM TEAB in 90 % methanol) was added to the sample and mixed. Samples were loaded onto the S-Trap column and trapped by centrifugation at 10,000 × g for 30 sec. Trapped proteins were washed 4 × with 150 μL of binding/wash buffer at 10,000 × g for 30 sec, followed by a final centrifugation at 10,000 × g for 1 min to dry the column. For protein digestion, Trypsin Gold Mass Spectrometry Grade (Promega) was resuspended at 1 μg/μL in 50 mM acetic acid (MS grade), aliquoted and stored at –80 °C. 2 μg of the resuspended Trypsin Gold was diluted in digestion buffer (50 mM TEAB) for a final volume of 20 μL and added to the S-Trap column. Columns were incubated overnight at 37 °C in a water bath without shaking. Peptides were eluted in three steps, by adding 40 μL of elution buffer 1 (50 mM TEAB), elution buffer 2 (0.2 % MS grade formic acid) and elution buffer 3 (50 % MS grade acetonitrile), each followed by a centrifugation step at 10,000 × g for 1 min. Pooled eluates were dried down at RT in a vacuum concentrator (RC 10.10, Jouan) and resuspended in 0.1 % formic acid (MS grade) for LC-MS/MS analysis.

Mouse livers were lysed via beads-beating in lysis buffer containing 8 M urea, 40 mM Tris/HCl (pH 8.5), 1x EDTA-free cOmplete protease inhibitor tablet (Roche). Proteins were digested with trypsin at 1:100 enzyme:protein ratio at 37 °C overnight. Digests were acidified by addition of neat formic acid (FA) to a final concentration of 5% (v/v). Digests were desalted using tC18 RP solid-phase extraction cartridges (Waters) and labeled with TMTpro.[1] TMT-labeled sample samples were reconstituted in 25 mM ammonium bicarbonate (pH 8) and fractionated via a C18 column (XBridgeBEH130, 3.5 μm, 2.1 x 150 mm, Waters). Peptides were eluted with increasing acetonitrile (ACN) concentration into 96 fractions and further pooled to 48 fractions.[2] Pooled fractions were dried down at RT in a vacuum concentrator and resuspended in 0.1 % formic acid for LC-MS/MS analysis.

Mass spectrometry

Bile samples were analyzed on a μLC-MS/MS system using a modified Vanquish pump coupled to a Q Exactive Orbitrap HF-X mass spectrometer (both Thermo Fisher Scientific). Chromatographic separation was performed via direct sample injection onto the head of a 15 cm Acclaim PepMap 100 C18 column (2 μm particle size, 1 mm ID, Thermo Fisher Scientific) at a flow rate of 50 μL/min.[3] Solvent A was 0.1 % FA, 3 % DMSO in water, and solvent B was 0.1 % FA, 3 % DMSO in ACN. Samples were separated with a linear gradient of 3 % to 28 % B in 30 min, and the total analysis time was 32 min. The IonMax source was used to acquire the data with HESI-II probe depth at "position A". The mass spectrometer was operated in positive ion mode using an electrospray voltage of 3.5 kV, capillary temperature of 325 °C and vaporizer temperature of 125 °C. The flow rates of sheath gas, aux gas and sweep gas were set to 32, 5, and 0, respectively. A data-dependent acquisition method was used, which automatically switched between MS and MS/MS. Survey full-scan MS spectra were recorded in the orbitrap at a resolution of 60,000 at m/z 200 and an AGC target value of 3e6 with a maximum injection time (maxIT) of 50 ms. The MS1 mass range was set to 360–1300. The isolation width was set to 1.3 m/z, and the first mass was fixed at 100 m/z. After the survey scan, the 12 most abundant precursors passing the intensity threshold of 9.1e4 were selected for HCD fragmentation, and MS2 spectra were recorded in the orbitrap with an AGC target value of 1e5 and maxIT of 22 ms. The normalized collision energy was set to 28. MS1 and MS2 spectra were acquired in profile and centroid mode, respectively. The dynamic exclusion value was set to 15 s.

Liver samples were analyzed on an Orbitrap Eclipse Tribrid mass spectrometer coupled to a Dionex UltiMate 3000 RSLCnano System (both Thermo Fisher Scientific). Samples were injected onto a trap column (75 µm × 2 cm, packed in-house with 5 µm C18 resin, Dr. Maisch), washed with 0.1% FA (5 µL/min, 10 min) and subsequently transferred to an analytical column (75 µm × 48 cm, packed in-house with 3 µm C18 resin; Reprosil Gold, Dr. Maisch). Peptides were separated at 300 nL/min using a 50-min linear gradient from 8 to 34% solvent B (0.1% FA, 5% DMSO in ACN) in solvent A (0.1% FA in 5% DMSO in water). The MS was operated in positive polarity and data-dependent MS3-mode. Every 3 s, a full scan (MS1) was recorded from 360 to 1500 m/z at a resolution of 60k in the Orbitrap in profile mode. The MS1 automatic gain control (AGC) target was set at 100%, and the maximal injection time (maxIT) was set at 50 ms. Based on the full scans, precursors were targeted for MS2 scans if the charge was between 2 and 6. The MS2 quadrupole isolation window was set at 0.7 Th. Peptides were fragmented via HCD (NCE of 34%). The MS2 spectrum was acquired with 15k resolution in the Orbitrap. The MS2 AGC target was set at 200%, and the maxIT was set at 22 ms. Precursors that have been targeted for fragmentation were excluded for 60 s. TMT reporter ions were measured in a consecutive MS3 scan based on the previous MS2 scan. The top 10 fragment ions of the MS2 scans were isolated in the ion trap in parallel (synchronous precursor selection; SPS) and further, HCD-fragmented with an NCE of 55%. The MS3 spectrum was acquired with 30k resolution in the Orbitrap, with the enhanced resolution mode set to TMT. The MS3 AGC target was set at 200%, and the maxIT was set at 54 ms.

Data Processing and Analysis

Raw data files were processed with MaxQuant.[4](version 1.6.10.43) or Proteome Discoverer (version 2.5) and Sequest HT, using the default parameters. Spectra were searched against the UniProtKB database (*Mus musculus*, UP000000589, 55,431 entries downloaded on 12.2019, or *Homo sapiens*, UP000005640, 79,071 entries downloaded on 02.2021). Enzyme specificity was set to trypsin and up to 2 missed cleavages were allowed. Cysteine carbamidomethylation or beta-methylthiolation was set as a fixed modification while protein N-term acetylation and methionine oxidation were selected as variable modifications. For liver samples, TMTpro (peptide n-termini and lysine) was set as a fixed modification. Precursor tolerance was set at 5 ppm, and fragment ion tolerance at 20 ppm. Results were adjusted to 1 % false discovery rate at protein and peptide levels. Identifications were filtered to remove contaminants and decoy hits using Perseus [5] (v. 1.6.13.0) before any subsequent analysis. To identify bile proteins of putative pancreatic origin, the dataset was compared to 65 human proteins classified as “enriched in pancreas tissue” by the Human Protein Atlas (proteinatlas.org) [6].

References

1. Zecha J, Satpathy S, Kanashova T, et al. TMT Labeling for the Masses: A Robust and Cost-efficient, In-solution Labeling Approach. Mol Cell Proteomics 2019;18:1468-1478.
2. Chang YC, Gnann C, Steimbach RR, et al. Decrypting lysine deacetylase inhibitor action and protein modifications by dose-resolved proteomics. Cell Rep 2024;43:114272.
3. Bian Y, Zheng R, Bayer FP, et al. Robust, reproducible and quantitative analysis of thousands of proteomes by micro-flow LC-MS/MS. Nat Commun 2020;11:157.
4. Cox J, Mann M. MaxQuant enables high peptide identification rates, individualized p.p.b.-range mass accuracies and proteome-wide protein quantification. Nat Biotechnol 2008;26:1367-72.
5. Tyanova S, Cox J. Perseus: A Bioinformatics Platform for Integrative Analysis of Proteomics Data in Cancer Research. Methods Mol Biol 2018;1711:133-148.
6. Uhlen M, Fagerberg L, Hallstrom BM, et al. Proteomics. Tissue-based map of the human proteome. Science 2015;347:1260419.
