# Supplementary material for: Gut microbiota restricts intestinal lipid uptake via modulation of bile phosphatidylcholine metabolism in mice
Source: Nat Microbiol. 2026 Jul 29;11(8):2349–64. doi: 10.1038/s41564-026-02434-z (PMC13423828; doi:10.1038/s41564-026-02434-z)
Supplement: Supplementary file 2 — Reporting summary [file 41564_2026_2434_MOESM2_ESM.pdf]

nature portfolio

Corresponding author(s): Josef Ecker

Last updated by author(s): May 13, 2026

Reporting Summary

Nature Portfolio wishes to improve the reproducibility of the work that we publish. This form provides structure for consistency and transparency in reporting. For further information on Nature Portfolio policies, see our Editorial Policies and the Editorial Policy Checklist.

Statistics

For all statistical analyses, confirm that the following items are present in the figure legend, table legend, main text, or Methods section.

n/a	Confirmed	
☐	☒	The exact sample size (*n*) for each experimental group/condition, given as a discrete number and unit of measurement
☐	☒	A statement on whether measurements were taken from distinct samples or whether the same sample was measured repeatedly
☐	☒	The statistical test(s) used AND whether they are one- or two-sided<br>*Only common tests should be described solely by name; describe more complex techniques in the Methods section.*
☐	☒	A description of all covariates tested
☐	☒	A description of any assumptions or corrections, such as tests of normality and adjustment for multiple comparisons
☐	☒	A full description of the statistical parameters including central tendency (e.g. means) or other basic estimates (e.g. regression coefficient) AND variation (e.g. standard deviation) or associated estimates of uncertainty (e.g. confidence intervals)
☐	☒	For null hypothesis testing, the test statistic (e.g. *F*, *t*, *r*) with confidence intervals, effect sizes, degrees of freedom and *P* value noted<br>*Give P values as exact values whenever suitable.*
☒	☐	For Bayesian analysis, information on the choice of priors and Markov chain Monte Carlo settings
☒	☐	For hierarchical and complex designs, identification of the appropriate level for tests and full reporting of outcomes
☒	☐	Estimates of effect sizes (e.g. Cohen's *d*, Pearson's *r*), indicating how they were calculated

Our web collection on statistics for biologists contains articles on many of the points above.

Software and code

Policy information about availability of computer code

Data collection	The code for the physiology-based kinetic modelling and liver proteomics analysis is available at: https://github.com/mszimmermann/Microbiome_effect_on_lipid_metabolism.
Data analysis	The code for the physiology-based kinetic modelling and liver proteomics analysis is available at: https://github.com/mszimmermann/Microbiome_effect_on_lipid_metabolism.

For manuscripts utilizing custom algorithms or software that are central to the research but not yet described in published literature, software must be made available to editors and reviewers. We strongly encourage code deposition in a community repository (e.g. GitHub). See the Nature Portfolio guidelines for submitting code & software for further information.

Data

Policy information about availability of data

All manuscripts must include a data availability statement. This statement should provide the following information, where applicable:

- Accession codes, unique identifiers, or web links for publicly available datasets
- A description of any restrictions on data availability
- For clinical datasets or third party data, please ensure that the statement adheres to our policy

Mass spectrometry bile and liver proteomics data have been deposited in the ProteomeXchange Consortium via the PRIDE partner repository with the dataset identifier PXD033894 and PXD060110. The publicly available RNAseq and Affymetrix Microarray data downloaded from Expression Atlas (https://www.ebi.ac.uk/

gxa/home) are deposited in Zenodo archive at: https://zenodo.org, DOI 10.5281/zenodo.15092709. Lipidomics data, model parameters and equations used for physiology-based kinetic multi-compartment modelling are reported in Supplementary Data 1. The data underlying the Figures and Extended Data Figures are supplied in the Source Data files.

Research involving human participants, their data, or biological material

Policy information about studies with human participants or human data. See also policy information about sex, gender (identity/presentation), and sexual orientation and race, ethnicity and racism.

Reporting on sex and gender	Bile (1 sample) was obtained from a female (sex) patient (38y) undergoing cholecystectomy (no malignant disease) at the Department of Surgery (TUM, Munich, Germany).
Reporting on race, ethnicity, or other socially relevant groupings	n/a
Population characteristics	n/a
Recruitment	n/a
Ethics oversight	Ethics are approved by the Ethics Committee of the School of Medicine and Health at the Technical University of Munich/ TUM (# 1926/07;#5428/12).

Note that full information on the approval of the study protocol must also be provided in the manuscript.

Field-specific reporting

Please select the one below that is the best fit for your research. If you are not sure, read the appropriate sections before making your selection.

☒ Life sciences ☐ Behavioural & social sciences ☐ Ecological, evolutionary & environmental sciences

For a reference copy of the document with all sections, see nature.com/documents/nr-reporting-summary-flat.pdf

Life sciences study design

All studies must disclose on these points even when the disclosure is negative.

Sample size	For all mouse experiments animal group/sample sizes were estimated according to personal experience. They primarily depend on animal availability and breeding.
Data exclusions	Data/samples were only excluded, when there were obvious problems in the animal experiments, sampling or in the technical measurements.
Replication	Mass spectrometric analyses included quality controls to ensure precision and accuracy. Proteomic analyses were not replicated, due to limited amounts of sample material, time and costs.
Randomization	Omics analyses were randomized.
Blinding	Omics analyses were blinded.

Reporting for specific materials, systems and methods

We require information from authors about some types of materials, experimental systems and methods used in many studies. Here, indicate whether each material, system or method listed is relevant to your study. If you are not sure if a list item applies to your research, read the appropriate section before selecting a response.

Materials & experimental systems

n/a	Involved in the study	
☐	☒	Antibodies
☐	☒	Eukaryotic cell lines
☒	☐	Palaeontology and archaeology
☐	☒	Animals and other organisms
☒	☐	Clinical data
☒	☐	Dual use research of concern
☒	☐	Plants

Methods

n/a	Involved in the study	
☒	☐	ChIP-seq
☒	☐	Flow cytometry
☒	☐	MRI-based neuroimaging

Antibodies

Antibodies used	HSP90 (4877S Cell Signaling, 1:1000), MYD88 (4283S Cell Signaling, 1:500) and anti-rabbit HRP secondary antibody (7074P2 Cell signaling, 1:4000).
Validation	HSP90 (C45G5) Rabbit Monoclonal Antibody detects endogenous levels of total HSP90 protein. This antibody does not cross-react with other HSPs. Species Reactivity: Human, Mouse, Rat, Monkey. MyD88 (D80F5) Rabbit Monoclonal Antibody detects endogenous levels of total MyD88 protein. Species Reactivity: Human, Mouse, Rat, Hamster, Monkey. From the manufactures websites.

Eukaryotic cell lines

Policy information about cell lines and Sex and Gender in Research

Cell line source(s)	HepG2 from ATCC, HB-8065
Authentication	Authenticated by STR profiling (ExPasy Cellosaurus) in 2025.
Mycoplasma contamination	The cell line was tested negative for mycoplasma contamination
Commonly misidentified lines (See ICLAC register)	*Name any commonly misidentified cell lines used in the study and provide a rationale for their use.*

Animals and other research organisms

Policy information about studies involving animals; ARRIVE guidelines recommended for reporting animal research, and Sex and Gender in Research

Laboratory animals	C57BL/6JZtm; Wildtype, Cyp2c70-/-, Myd88-/-, Cel-/-
Wild animals	n/a
Reporting on sex	Male and female mice were mixed for the experiments performed in this study. We did not find any sex-dependent results.
Field-collected samples	n/a
Ethics oversight	All mouse experiments were approved by the General Administration of Bavaria (#55.2-1-54-2532-192-2016,#ROB-55.2-2532.Vet_02-21-124), by the Swiss Kantonal authorities (License ZH120/19 and ZH058/19; Kantonales Veterinäramt Zürich)

Note that full information on the approval of the study protocol must also be provided in the manuscript.

Plants

Seed stocks	*Report on the source of all seed stocks or other plant material used. If applicable, state the seed stock centre and catalogue number. If plant specimens were collected from the field, describe the collection location, date and sampling procedures.*
Novel plant genotypes	*Describe the methods by which all novel plant genotypes were produced. This includes those generated by transgenic approaches, gene editing, chemical/radiation-based mutagenesis and hybridization. For transgenic lines, describe the transformation method, the number of independent lines analyzed and the generation upon which experiments were performed. For gene-edited lines, describe the editor used, the endogenous sequence targeted for editing, the targeting guide RNA sequence (if applicable) and how the editor was applied.*
Authentication	*Describe any authentication procedures for each seed stock used or novel genotype generated. Describe any experiments used to assess the effect of a mutation and, where applicable, how potential secondary effects (e.g. second site T-DNA insertions, mosiacism, off-target gene editing) were examined.*
