# Supplementary figures and images for: Gut microbiota restricts intestinal lipid uptake via modulation of bile phosphatidylcholine metabolism in mice
Source: Nat Microbiol. 2026 Jul 29;11(8):2349–64. doi: 10.1038/s41564-026-02434-z (PMC13423828; doi:10.1038/s41564-026-02434-z)

## Ponceau staining (whole protein)

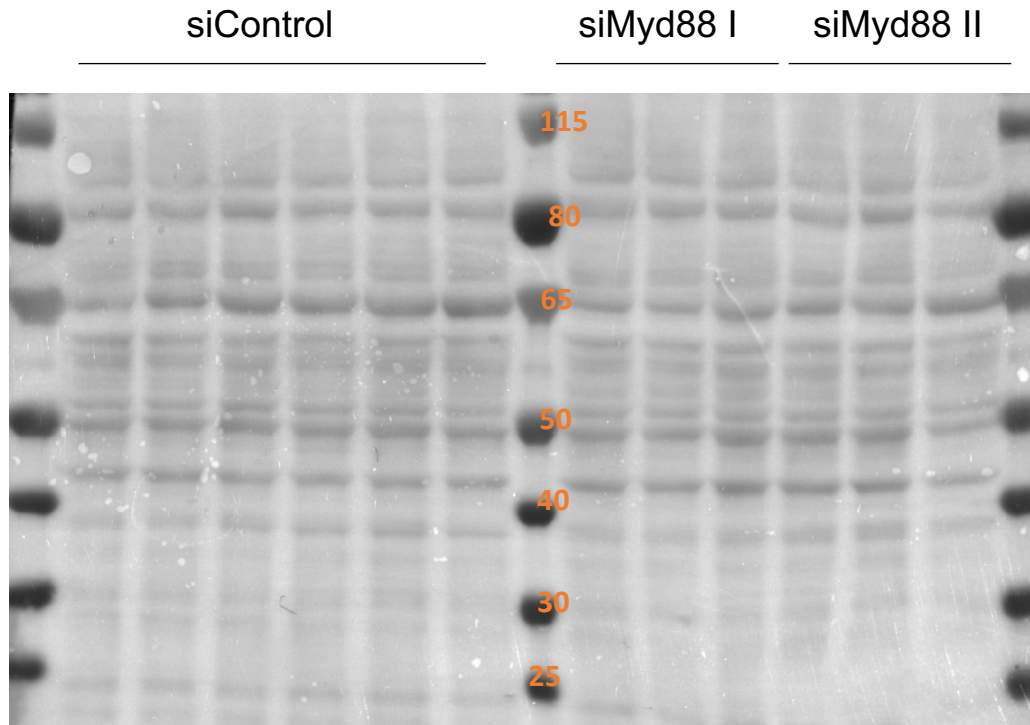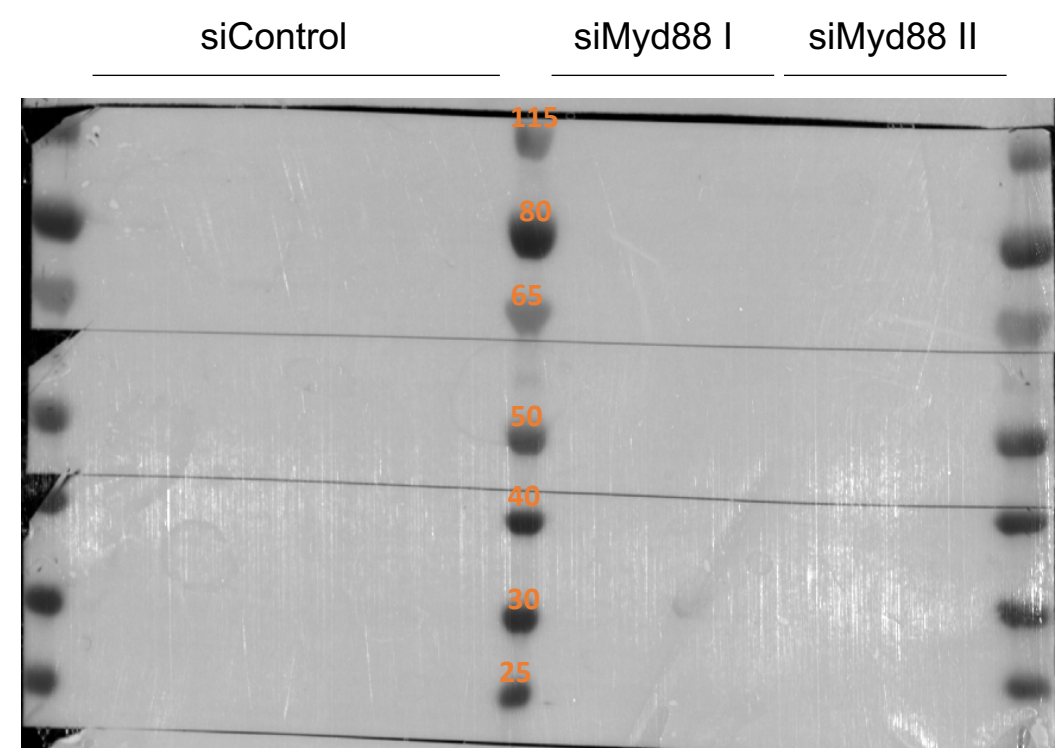

Pageruler 22616 (MOPS/BIS-Tris 4-12%)

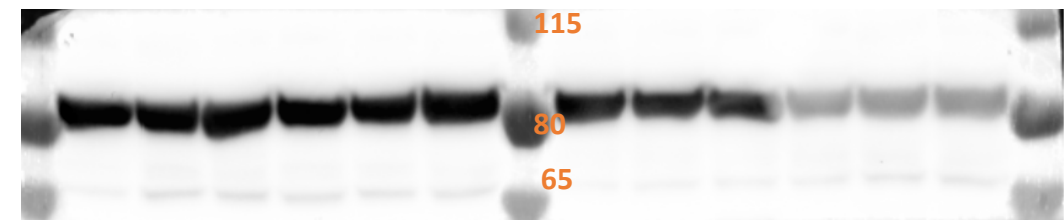

**HSP90 1:1000 (~90 kDa)**

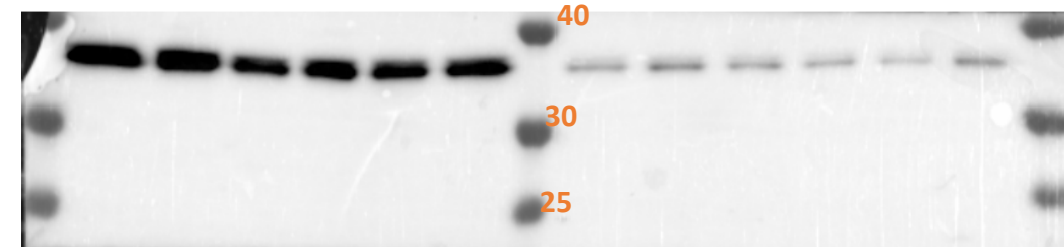

**Myd88 1:1000 (~35 kDa)**

Supplement: Supplementary file 12 — Unprocessed western blots. [file 41564_2026_2434_MOESM12_ESM.pdf]
