# Supplementary material for: Gut microbiota restricts intestinal lipid uptake via modulation of bile phosphatidylcholine metabolism in mice
Source: Nat Microbiol. 2026 Jul 29;11(8):2349–64. doi: 10.1038/s41564-026-02434-z (PMC13423828; doi:10.1038/s41564-026-02434-z)
Supplement: Supplementary file 3 — Peer Review File [file 41564_2026_2434_MOESM3_ESM.pdf]

nature portfolio

Peer Review File

Gut microbiota restricts intestinal lipid uptake via modulation of bile phosphatidylcholine metabolism in mice

Corresponding Author: Professor Josef Ecker

Version 0:

Reviewer comments:

Reviewer #1

(Remarks to the Author)
The authors describe a link between lipid absorption and the gut microbiota. They propose a connection between myd88, PLA1, and bile acid metabolism. I think the labeling experiments are well done and add key quantitative insight into their manuscript. However, the mechanistic insight is limited. For example, what factor(s) from the gut microbiota drive these changes? They have provided evidence that the gut microbiota is important but no evidence about how the gut microbiota is actually driving this change.

Significant issues:

1. GF mice are hyperphagic. Is it possible that differences in food intake contributes to the changes the authors have observed? I wonder if a 2h fast is long enough? I am also surprised by the transit time data. It is well recognized that germ free and conventional mice have different gut motilities and hence transit times. Further, male and females also show differences in gut motility. This has been widely reported. How do the authors account for this difference in their data?

2. There are important contributions to bile acid metabolism from the nuclear receptor FXR and bacterial enzymes like bile salt hydrolase. How are these key host and microbial factors impacting this mechanism? This seems important since GF mice lack any BSH activity and will also have significantly different FXR activity (among many other nuclear receptors like CAR which they did not follow up on). Further the BSH activity in OMM12 vs conventional mice is also significantly different and hence could be a driving factor in the bile acid pools.

3. Based on the methods, it appears that the authors used a combination of male and female mice. I certainly appreciate challenges due to cost, breeding, etc but how balanced are the various groups? The figures should be labeled such that the male and female datapoints are clearly visible. Also it seems appropriate to assess statistically if there are sex-specific differences.

Other comments:

1. How and when was the bile collected?
2. Sample size in some cases seems to be an issue. For example, Figure 3D. What explains the significant variation in the GF mice? Is sex specific?

(Remarks on code availability)

Reviewer #2

(Remarks to the Author)
The study of Plagge et al reports that the gut microbiota decreases lipid absorption by inducing a phospholipase activity in bile that reduces glycerophospholipids required for emulsification. The study provides evidence that overlooked enzymatic reactions in the bile are regulators of lipid absorption, making it a potential therapeutic target. The authors also provide data about a potential strain in the microbiome that contributes to the phenotype, as well as signaling pathways implicated. However, the data seems to show only weak effects on these aspects.

Strong points of the study:
The implication of lipid metabolic reactions within the bile, rather than in the intestinal lumen, is very interesting.
The authors perform extensive proteomics and enzymology to provide convincing mechanistic explanations for bile lipid changes.

Major issues:
1. The methods are too briefly written, not allowing us to know what has been done and if the experiments are correctly designed. For example, for GC-MS, we cannot know if they analyze only free fatty acids or they include esterified ones. How the derivatization was done (or if it was done because there is really nothing explained, unless I missed a supplemental material) is also unclear. Similarly, there is little clue about how lipidomics was done.
2. The authors imply that Akkermansia muciniphila is implicated in the inhibition of lipid uptake. This is concluded from a comparison of OMM11 and OMM12, but the evidence is weak. In Figure 4B-D, the difference between OMM11 and OMM12 is not statistically significant, and the individual plots give the impression that the difference, if any, is very small. Even if there was statistical significance, a comparison of the averages would suggest that the contribution of this single strain is minor. Consider removing the data, or add more evidence.
3. The data that suggest the implication of Myd88 in microbiome-dependent lipid uptake regulation are weak. In Figure 7I, the change in Cyp7b1 levels is very minor compared to the drastic changes seen in Figure 7C. This shows that the role of Myd88 is minor and redundant. Similarly, the changes in bile acids are small in Figure 7JK and are not comparable with the changes in germ free versus SPF mice (Figure 6A). The changes in phospholipase A1 activity are large (Figure 7L), but no changes in lysophosphatidylcholine are reported, which makes me wondering why the experiment was not done. In other words, we see relatively large changes in phospholipase A1 activity without big changes in bile composition, which is against the claims made in Figure 6. Thus, without clarification, this could overturn the major claims of the study.
4. The gut microbiome induces large changes in bile acids. This in itself should have an effect on lipid uptake, regardless of phosphatidylcholine levels. Without comparison of germ free and SPF in a carboxyl ester lipase knockout background, it is difficult to discriminate the contribution of bile acids and phosphatidylcholine. Of course, any other experiment that would help the discrimination can replace the one I suggested.

Minor issues:
5. I have the impression that the values in Figure 1B and 1C-F do not match. For example, in Figure 1C, we see no difference at 6H, but in Figure 1B it says that FA 16:0[D5] is half.
6. I am not sure how useful the modeling in Figure 2 was. In Figure 2C, it seems that it does not predict very well many of the data. We do not see the full set of predictions in the supplements, and thus I am not convinced. The authors do not provide explanation on parameters that would provide the goodness of the initial model neither.
7. How were bile samples stored? This could affect the production of lysophosphatidylcholine.
8. The authors often use correlation plots (Figures 1EF, 6BG), but I believe they are often misused and are irrelevant for the conclusions. If two variables, for example TCA and LPC concentrations, change in the same direction between groups, it is quite normal that a positive correlation would appear. In that case, the positive correlation would just reflect the group effect, and does not necessarily show any causality between the variables. Plots would be relevant only if there is correlation even within groups, but with the small numbers that is often used in the study, this is hard to say. For example, in Figure 6B, the right panel suggests that TBMCA negatively correlates with LPC, but if we just look at OMM12 the correlation seems to be positive. Most of the plots can be simply replaced by different graphs that show the changes in variables separately, to show that they go on the same or opposite direction. This will be more correct without affecting the conclusions.
9. The authors propose that phospholipase A1 activity is affected by bile acid levels, rather than the expression levels of enzymes. This would be correct only if TBMCA or TCDCA have no effect on phospholipase A1 activity. Please refer to appropriate studies to show if it’s the case, or test it experimentally.
10. The protein data in Figure 7C would be strengthened if the authors analyze mRNA levels of relevant targets too. It will show if the changes occur at the transcriptional level, and this would make the association with Figure 7I better, as the latter provides mRNA data.
11. The comparison between deuterated fatty acid and deuterated triglyceride is clever, as it helps the discrimination between lipid digestion and lipid uptake. The authors explain this in the discussion. If the authors mention the rationale of this design earlier, for example early in the results, a reader would understand better why the experiment is done like this.

(Remarks on code availability)

Reviewer #3

(Remarks to the Author)
In this study, Johannes et al. systematically investigated how the gut microbiota influences host lipid absorption and the underlying molecular mechanisms. The authors propose that the microbiota suppresses hepatic Cyp7b1 activity, increases taurocholate synthesis, activates a phospholipase A1 in bile, accelerates phosphatidylcholine degradation, and ultimately inhibits intestinal lipid uptake. The work is methodologically rich, thereby uncovering a “microbiota–bile–lipid-absorption” axis. However, the causal evidence remains incomplete, and additional experiments are required for full validation.

Major
1. The higher absorption observed in GF mice contradicts previous reports (PMID: 37616368, 29649441). Please provide some explanation. Whether the housing conditions for GF, OMM12 and SPF mice in this study identical? Was there no difference in body weight among the three groups? If the GF mice were significantly lighter, an identical lipid gavage would yield a higher dose-to-weight ratio and could potentially exaggerating absorption. How do the authors explain lower body weight of age-matched GF versus SPF mice? Moreover, several groups have reported reduced intestinal lipid absorption after antibiotic treatment; how do the authors interpret?
2. The conclusion of the article states that "the gut microbiota limits fatty acid absorption from the gut lumen into intestinal tissue." However, in Figure 1B and 1E, the levels of FA16:0[D5] and FA16:0[D31] in the ileum tissue of SPF mice and OMM12 mice are higher than those in GF mice. Additionally, in Figure 1B, no reduction in lipids is observed in the duodenum tissue—the primary site of lipid absorption—in SPF and OMM12 mice compared to GF mice. Does this contradict the conclusion of the article?

3. Studies have reported lower intestinal CD36 expression in GF than in SPF mice (PMID: 28860383), yet Figure 3A shows the opposite trend. Please increase sample size and add Western blotting analysis to strengthen this conclusion. Studies have shown that the gut microbiota, such as Lactobacillus, can influence the palmitoylation of CD36 (PMID: 40767021). Additionally, the bile acid receptor TGR5 (for which TCA is a weak agonist) can also affect the palmitoylation and membrane localization of CD36 (PMID: 38698281). Therefore, the role of CD36 in the gut microbiota-lipid absorption axis requires further discussion.
4. The authors used physiology-based kinetic modelling to attribute the differences among the three tracers to “a single reduced absorption coefficient k_abs.” Please corroborate this conclusion with independent experiments such as a lymph-fistula model or a dual-isotope intestinal-flux assay.
5. Figure 3 only presents the relative composition 6 h post-gavage. Please provide absolute concentrations of lipids from GF, OMM12 and SPF mice, with statistical comparisons.
6. Tβ/ωMCA concentrations are negatively correlated with LPC levels, and the amounts of some unconjugated bile acids (e.g., CA) may also differ markedly among the mouse groups. How to determine which metabolite has the greatest impact on lipid absorption. Human bile is dominated by glycine-conjugated CA and CDCA. Could other bile acids modulate CEL hydrolytic activity?
7. An in vitro pure enzyme reaction demonstrating that CEL degrades PC to form LPC is required to confirm that CEL is the enzyme responsible for PC degradation in bile. Additionally, although the expression level of CEL remains unchanged in GF, OMM12, and SPF mice, how is its activity regulated? Further in vitro pure enzyme reactions should be supplemented to investigate whether TCA promotes the degradation of PC to LPC by CEL.
8. The authors could supplement the study with WT/MYD88-/- mice under different gut microbiota conditions (GF/SPF) to further elucidate the role of the MYD88-CYP7B1 signaling pathway in mediating the effects of gut microbiota on lipid absorption.

Minor
1. Supplement the PC composition of bile in GF, OMM12, OMM11, and SPF mice. Is there a difference in the content of PC34:2 in the bile of these mice?
2. Please list the exact species/strains comprising the OMM12 consortium in the Methods section.
3. Why is the total bile-acid concentration lower in OMM12 than in SPF and GF bile (Figure S6A)?
4. “Lipid” may imply both fat and cholesterol. Please discuss whether the TCA—PC/LPC axis also affects intestinal cholesterol absorption.
5. Have the authors used monocolonization experiments to verify the independent contribution of Akkermansia muciniphila to the TCA—PC/LPC axis and lipid absorption? Besides Akkermansia muciniphila, have the other bacteria in Oligo-MM12 been reported to affect lipid absorption?
6. In addition to influencing bile acid synthesis pathways in the liver via MYD88, the gut microbiota itself can perform various modifications on bile acids such as TCA, thereby affecting the content and proportion of bile acids in bile. The authors should provide a more detailed elaboration on this aspect.

(Remarks on code availability)

Decision Letter:

10th September 2025

Dear Professor Ecker,

Thank you for your patience while your manuscript "Intestinal lipid uptake is restricted by the gut microbiota via regulation of phosphatidylcholine metabolism in bile" was under peer review at Nature Microbiology. It has now been seen by our referees, whose expertise and comments you will find at the end of this email. In the light of their advice, we have decided that we cannot offer to publish your manuscript in Nature Microbiology.

From the reports, you will see that while they find your work of some potential interest, the referees raise concerns regarding weak mechanistic insights, lack of experimental support to the causal inferences, some technical issues, and some contradictory findings. Unfortunately, these criticisms are sufficiently important as to preclude publication of your work in Nature Microbiology.

Although we cannot offer to publish your manuscript, I have consulted with, Javier Martinez-Vesga, my colleague at Nature Communications, and he indicated that he would be willing to send an appropriately revised manuscript back to the referees, if transferred after revision. You can contact Javier directly to discuss it further at javier.martinez-vesga@nature.com

To transfer your manuscript please use our manuscript transfer portal. You will not have to re-supply manuscript metadata and files, unless you wish to make modifications. For more information, please see our manuscript transfer FAQ page.

I am sorry that we cannot be more positive on this occasion, but hope that you find the referees' comments helpful when preparing your paper for resubmission elsewhere.

Yours sincerely,

****
Reviewer Expertise:

Referee #1: Bile acid metabolism
Referee #2: host lipid metabolism
Referee #3: Microbiome, metabolism

Reviewers Comments:

Reviewer #1 (Remarks to the Author):

The authors describe a link between lipid absorption and the gut microbiota. They propose a connection between myd88, PLA1, and bile acid metabolism. I think the labeling experiments are well done and add key quantitative insight into their manuscript. However, the mechanistic insight is limited. For example, what factor(s) from the gut microbiota drive these changes? They have provided evidence that the gut microbiota is important but no evidence about how the gut microbiota is actually driving this change.

Significant issues:

1. GF mice are hyperphagic. Is it possible that differences in food intake contributes to the changes the authors have observed? I wonder if a 2h fast is long enough? I am also surprised by the transit time data. It is well recognized that germ free and conventional mice have different gut motilities and hence transit times. Further, male and females also show differences in gut motility. This has been widely reported. How do the authors account for this difference in their data?

2. There are important contributions to bile acid metabolism from the nuclear receptor FXR and bacterial enzymes like bile salt hydrolase. How are these key host and microbial factors impacting this mechanism? This seems important since GF mice lack any BSH activity and will also have significantly different FXR activity (among many other nuclear receptors like CAR which they did not follow up on). Further the BSH activity in OMM12 vs conventional mice is also significantly different and hence could be a driving factor in the bile acid pools.

3. Based on the methods, it appears that the authors used a combination of male and female mice. I certainly appreciate challenges due to cost, breeding, etc but how balanced are the various groups? The figures should be labeled such that the male and female datapoints are clearly visible. Also it seems appropriate to assess statistically if there are sex-specific differences.

Other comments:

1. How and when was the bile collected?
2. Sample size in some cases seems to be an issue. For example, Figure 3D. What explains the significant variation in the GF mice? Is sex specific?

Reviewer #2 (Remarks to the Author):

The study of Plagge et al reports that the gut microbiota decreases lipid absorption by inducing a phospholipase activity in bile that reduces glycerophospholipids required for emulsification. The study provides evidence that overlooked enzymatic reactions in the bile are regulators of lipid absorption, making it a potential therapeutic target. The authors also provide data about a potential strain in the microbiome that contributes to the phenotype, as well as signaling pathways implicated. However, the data seems to show only weak effects on these aspects.

Strong points of the study:
The implication of lipid metabolic reactions within the bile, rather than in the intestinal lumen, is very interesting.
The authors perform extensive proteomics and enzymology to provide convincing mechanistic explanations for bile lipid changes.

Major issues:
1. The methods are too briefly written, not allowing us to know what has been done and if the experiments are correctly designed. For example, for GC-MS, we cannot know if they analyze only free fatty acids or they include esterified ones. How the derivatization was done (or if it was done because there is really nothing explained, unless I missed a supplemental material) is also unclear. Similarly, there is little clue about how lipidomics was done.
2. The authors imply that Akkermansia muciniphila is implicated in the inhibition of lipid uptake. This is concluded from a comparison of OMM11 and OMM12, but the evidence is weak. In Figure 4B-D, the difference between OMM11 and OMM12 is not statistically significant, and the individual plots give the impression that the difference, if any, is very small. Even if there was statistical significance, a comparison of the averages would suggest that the contribution of this single strain is minor. Consider removing the data, or add more evidence.
3. The data that suggest the implication of Myd88 in microbiome-dependent lipid uptake regulation are weak. In Figure 7I, the change in Cyp7b1 levels is very minor compared to the drastic changes seen in Figure 7C. This shows that the role of Myd88 is minor and redundant. Similarly, the changes in bile acids are small in Figure 7JK and are not comparable with the changes in germ free versus SPF mice (Figure 6A). The changes in phospholipase A1 activity are large (Figure 7L), but no changes in lysophosphatidylcholine are reported, which makes me wondering why the experiment was not done. In other words, we see relatively large changes in phospholipase A1 activity without big changes in bile composition, which is against the claims made in Figure 6. Thus, without clarification, this could overturn the major claims of the study.
4. The gut microbiome induces large changes in bile acids. This in itself should have an effect on lipid uptake, regardless of

phosphatidylcholine levels. Without comparison of germ free and SPF in a carboxyl ester lipase knockout background, it is difficult to discriminate the contribution of bile acids and phosphatidylcholine. Of course, any other experiment that would help the discrimination can replace the one I suggested.

Minor issues:
5. I have the impression that the values in Figure 1B and 1C-F do not match. For example, in Figure 1C, we see no difference at 6H, but in Figure 1B it says that FA 16:0[D5] is half.
6. I am not sure how useful the modeling in Figure 2 was. In Figure 2C, it seems that it does not predict very well many of the data. We do not see the full set of predictions in the supplements, and thus I am not convinced. The authors do not provide explanation on parameters that would provide the goodness of the initial model neither.
7. How were bile samples stored? This could affect the production of lysophosphatidylcholine.
8. The authors often use correlation plots (Figures 1EF, 6BG), but I believe they are often misused and are irrelevant for the conclusions. If two variables, for example TCA and LPC concentrations, change in the same direction between groups, it is quite normal that a positive correlation would appear. In that case, the positive correlation would just reflect the group effect, and does not necessarily show any causality between the variables. Plots would be relevant only if there is correlation even within groups, but with the small numbers that is often used in the study, this is hard to say. For example, in Figure 6B, the right panel suggests that TBMCA negatively correlates with LPC, but if we just look at OMM12 the correlation seems to be positive. Most of the plots can be simply replaced by different graphs that show the changes in variables separately, to show that they go on the same or opposite direction. This will be more correct without affecting the conclusions.
9. The authors propose that phospholipase A1 activity is affected by bile acid levels, rather than the expression levels of enzymes. This would be correct only if TBMCA or TCDCA have no effect on phospholipase A1 activity. Please refer to appropriate studies to show if it's the case, or test it experimentally.
10. The protein data in Figure 7C would be strengthened if the authors analyze mRNA levels of relevant targets too. It will show if the changes occur at the transcriptional level, and this would make the association with Figure 7I better, as the latter provides mRNA data.
11. The comparison between deuterated fatty acid and deuterated triglyceride is clever, as it helps the discrimination between lipid digestion and lipid uptake. The authors explain this in the discussion. If the authors mention the rationale of this design earlier, for example early in the results, a reader would understand better why the experiment is done like this.

Reviewer #3 (Remarks to the Author):

In this study, Johannes et al. systematically investigated how the gut microbiota influences host lipid absorption and the underlying molecular mechanisms. The authors propose that the microbiota suppresses hepatic Cyp7b1 activity, increases taurocholate synthesis, activates a phospholipase A1 in bile, accelerates phosphatidylcholine degradation, and ultimately inhibits intestinal lipid uptake. The work is methodologically rich, thereby uncovering a "microbiota–bile–lipid-absorption" axis. However, the causal evidence remains incomplete, and additional experiments are required for full validation.

Major
1. The higher absorption observed in GF mice contradicts previous reports (PMID: 37616368, 29649441). Please provide some explanation. Whether the housing conditions for GF, OMM12 and SPF mice in this study identical? Was there no difference in body weight among the three groups? If the GF mice were significantly lighter, an identical lipid gavage would yield a higher dose-to-weight ratio and could potentially exaggerating absorption. How do the authors explain lower body weight of age-matched GF versus SPF mice? Moreover, several groups have reported reduced intestinal lipid absorption after antibiotic treatment; how do the authors interpret?
2. The conclusion of the article states that "the gut microbiota limits fatty acid absorption from the gut lumen into intestinal tissue." However, in Figure 1B and 1E, the levels of FA16:0[D5] and FA16:0[D31] in the ileum tissue of SPF mice and OMM12 mice are higher than those in GF mice. Additionally, in Figure 1B, no reduction in lipids is observed in the duodenum tissue—the primary site of lipid absorption—in SPF and OMM12 mice compared to GF mice. Does this contradict the conclusion of the article?
3. Studies have reported lower intestinal CD36 expression in GF than in SPF mice (PMID: 28860383), yet Figure 3A shows the opposite trend. Please increase sample size and add Western blotting analysis to strengthen this conclusion. Studies have shown that the gut microbiota, such as Lactobacillus, can influence the palmitoylation of CD36 (PMID: 40767021). Additionally, the bile acid receptor TGR5 (for which TCA is a weak agonist) can also affect the palmitoylation and membrane localization of CD36 (PMID: 38698281). Therefore, the role of CD36 in the gut microbiota-lipid absorption axis requires further discussion.
4. The authors used physiology-based kinetic modelling to attribute the differences among the three tracers to "a single reduced absorption coefficient k_abs." Please corroborate this conclusion with independent experiments such as a lymph-fistula model or a dual-isotope intestinal-flux assay.
5. Figure 3 only presents the relative composition 6 h post-gavage. Please provide absolute concentrations of lipids from GF, OMM12 and SPF mice, with statistical comparisons.
6. Tβ/ωMCA concentrations are negatively correlated with LPC levels, and the amounts of some unconjugated bile acids (e.g., CA) may also differ markedly among the mouse groups. How to determine which metabolite has the greatest impact on lipid absorption. Human bile is dominated by glycine-conjugated CA and CDCA. Could other bile acids modulate CEL hydrolytic activity?
7. An in vitro pure enzyme reaction demonstrating that CEL degrades PC to form LPC is required to confirm that CEL is the enzyme responsible for PC degradation in bile. Additionally, although the expression level of CEL remains unchanged in GF, OMM12, and SPF mice, how is its activity regulated? Further in vitro pure enzyme reactions should be supplemented to investigate whether TCA promotes the degradation of PC to LPC by CEL.
8. The authors could supplement the study with WT/MYD88-/- mice under different gut microbiota conditions (GF/SPF) to further elucidate the role of the MYD88-CYP7B1 signaling pathway in mediating the effects of gut microbiota on lipid absorption.

Minor

1. Supplement the PC composition of bile in GF, OMM12, OMM11, and SPF mice. Is there a difference in the content of PC34:2 in the bile of these mice?
2. Please list the exact species/strains comprising the OMM12 consortium in the Methods section.
3. Why is the total bile-acid concentration lower in OMM12 than in SPF and GF bile (Figure S6A)?
4. “Lipid” may imply both fat and cholesterol. Please discuss whether the TCA—PC/LPC axis also affects intestinal cholesterol absorption.
5. Have the authors used monocolonization experiments to verify the independent contribution of Akkermansia muciniphila to the TCA—PC/LPC axis and lipid absorption? Besides Akkermansia muciniphila, have the other bacteria in Oligo-MM12 been reported to affect lipid absorption?
6. In addition to influencing bile acid synthesis pathways in the liver via MYD88, the gut microbiota itself can perform various modifications on bile acids such as TCA, thereby affecting the content and proportion of bile acids in bile. The authors should provide a more detailed elaboration on this aspect.

****

Version 1:

Decision Letter:

15th October 2025

Dear Professor Ecker,

Thank you for your letter asking us to reconsider our decision on your Article entitled "Intestinal lipid uptake is restricted by the gut microbiota via regulation of phosphatidylcholine metabolism in bile". I am sending this decision on behalf of Atin while he is on annual leave. After careful consideration we have decided that we would be willing to consider a revised version of your manuscript in line with your revision plan.

Along with your revised manuscript, you should also submit a separate point-by-point response to all of the concerns raised by the referees, in each case describing what changes have been made to the manuscript or, alternatively, if no action has been taken, providing a compelling argument for why that is the case. If we feel that a substantial attempt has been made to address the referees' comments, this response will be sent back to the referees - along with the revised manuscript - so that they can judge whether their concerns have been addressed satisfactorily or otherwise.

I should stress, however, that we would be reluctant to trouble our referees again unless we thought that their comments had been addressed in full.

When revising your paper:

- ensure it complies with our format requirements for Letters as set out in our guide to authors at www.nature.com/nmicrobiol/authors/index.html

- state in a cover note the length of the text, methods and legends; the number of references and the number of display items.

Please ensure that all correspondence is marked with your Nature Microbiology reference number in the subject line.

Please use the following link to submit your revised manuscript:

Link Redacted

We hope to receive your revised paper within four weeks. If you cannot send it within this time, please let us know so that we can close your file. In this event, we will still be happy to reconsider your paper at a later date so long as nothing similar has been accepted for publication at Nature Microbiology or published elsewhere in the meantime. Should you miss the four-week deadline and your paper is eventually published, the received date will be that of the revised, not the original, version.

I would appreciate it if you could tell me if you think you will be able to submit a revised manuscript, and also the likely timescale.

I look forward to hearing from you soon.

Yours sincerely,

Version 2:

Reviewer comments:

Reviewer #1

(Remarks to the Author)
The authors have been responsive to the technical and some of the conceptual issues raised in the previous review. It is still not clear what signals from the gut microbiota are responsible for these effects and as such I think claims about new mechanistic insight are overstated. That said, I do think the study is important and likely of interest to readers. I would suggest as compromise that the authors temper their conclusions to more accurately reflect their findings.

Remaining issues:

1. The motility issue is still unclear. There are numerous papers where this has been quantified and appears to be independent of diet. I pasted a few below but note these and others are fairly consistent. It still remains possible that reduced motility is a factor in their results.

https://www.sciencedirect.com/science/article/pii/S0016508513001042?via=ihub
https://www.sciencedirect.com/science/article/pii/S2589004224021205?via=ihub

I recognize the authors may not have the capacity to redo these experiments with a dye to track motility along with their labeled lipid gavage. That would be ideal of course. However, I suggest that they carefully acknowledge and discuss their data in contrast to what others have shown.

(Remarks on code availability)

Reviewer #2

(Remarks to the Author)
The authors did a great job in their additional experiments and corrections. The new data on CEL KO mice is especially valuable. I have only a few comments.

Line 484: I believe that (40767021) is a personal note about the reference, which should be removed from the text.

Figure 3A is not well organized, as we see the same genes in different columns. It could be different regions of the small intestine, which should be better labeled.

Typo in line 606: hydrolyzes should be hydrolase

Line 652-663: It is nice that the authors discuss other similar studies, but they do not compare them to their own study. It would be important to discuss why there seems to be some discrepancy between their data and the literature.

(Remarks on code availability)

Reviewer #3

(Remarks to the Author)
They have addressed all of my concerns. It’s OK for me now.

(Remarks on code availability)

Decision Letter:

Our ref: NMICROBIOL-25072607B

16th April 2026

Dear Josef,

Thank you for submitting your revised manuscript "Intestinal lipid uptake is restricted by the gut microbiota via regulation of phosphatidylcholine metabolism in bile" (NMICROBIOL-25072607B). It has now been seen by the original referees and their comments are below. The reviewers find that the paper has improved in revision, and therefore we'll be happy in principle to publish it in Nature Microbiology, pending minor revisions to satisfy the referees' final requests and to comply with our editorial and formatting guidelines.

If the current version of your manuscript is in a PDF format, please email us a copy of the file in an editable format (Microsoft Word or LaTex)-- we can not proceed with PDFs at this stage.

We are now performing detailed checks on your paper and will send you a checklist detailing our editorial and formatting requirements in about a week. Please do not upload the final materials and make any revisions until you receive this additional information from us.

Thank you again for your interest in Nature Microbiology Please do not hesitate to contact me if you have any questions.

Sincerely,

Reviewer #1 (Remarks to the Author):

The authors have been responsive to the technical and some of the conceptual issues raised in the previous review. It is still not clear what signals from the gut microbiota are responsible for these effects and as such I think claims about new mechanistic insight are overstated. That said, I do think the study is important and likely of interest to readers. I would suggest as compromise that the authors temper their conclusions to more accurately reflect their findings.

Remaining issues:

1. The motility issue is still unclear. There are numerous papers where this has been quantified and appears to be independent of diet. I pasted a few below but note these and others are fairly consistent. It still remains possible that reduced motility is a factor in their results.

https://www.sciencedirect.com/science/article/pii/S0016508513001042?via=ihub
https://www.sciencedirect.com/science/article/pii/S2589004224021205?via=ihub

I recognize the authors may not have the capacity to redo these experiments with a dye to track motility along with their labeled lipid gavage. That would be ideal of course. However, I suggest that they carefully acknowledge and discuss their data in contrast to what others have shown.

Reviewer #2 (Remarks to the Author):

The authors did a great job in their additional experiments and corrections. The new data on CEL KO mice is especially valuable. I have only a few comments.

Line 484: I believe that (40767021) is a personal note about the reference, which should be removed from the text.

Figure 3A is not well organized, as we see the same genes in different columns. It could be different regions of the small intestine, which should be better labeled.

Typo in line 606: hydrolyzes should be hydrolase

Line 652-663: It is nice that the authors discuss other similar studies, but they do not compare them to their own study. It would be important to discuss why there seems to be some discrepancy between their data and the literature.

Reviewer #3 (Remarks to the Author):

They have addressed all of my concerns. It’s OK for me now.

Version 3:

Decision Letter:

2nd June 2026

Dear Josef,

I am pleased to accept your Article "Gut microbiota restricts intestinal lipid uptake via modulation of bile phosphatidylcholine metabolism in mice" for publication in Nature Microbiology. It took a while but it is a journey that came to a happy ending! Thank you for choosing Nature Microbiology to showcase your work and many congratulations.

Over the next few weeks, your paper will be copyedited to ensure that it conforms to Nature Microbiology style. We look particularly carefully at the titles of all papers to ensure that they are relatively brief and understandable.

Once your paper is typeset, you will receive an email with a link to choose the appropriate publishing options for your paper and our Author Services team will be in touch regarding any additional information that may be required. Once your paper has been scheduled for online publication, the Nature press office will be in touch to confirm the details.

You may wish to make your media relations office aware of your accepted publication, in case they consider it appropriate to organize some internal or external publicity. Once your paper has been scheduled you will receive an email confirming the publication details. This is normally 3-4 working days in advance of publication. If you need additional notice of the date and time of publication, please let the production team know when you receive the proof of your article to ensure there is sufficient time to coordinate. Further information on our embargo policies can be found here:
https://www.nature.com/authors/policies/embargo.html

After the grant of rights is completed, you will receive a link to your electronic proof via email with a request to make any corrections within 48 hours. If, when you receive your proof, you cannot meet this deadline, please inform us at rjsproduction@springernature.com immediately. You will not receive your proofs until the publishing agreement has been received through our system

Due to the importance of these deadlines, we ask you please us know now whether you will be difficult to contact over the next month. If this is the case, we ask you provide us with the contact information (email, phone and fax) of someone who will be able to check the proofs on your behalf, and who will be available to address any last-minute problems.

Acceptance of your manuscript is conditional on all authors' agreement with our publication policies (see https://www.nature.com/nmicrobiol/editorial-policies). In particular your manuscript must not be published elsewhere.

Authors may need to take specific actions to achieve compliance with funder and institutional open access mandates. If your research is supported by a funder that requires immediate open access (e.g. according to Plan S principles or the NIH public access policy) then you should select the gold OA route, and we will direct you to the compliant route where possible. Because authors warrant under our subscription licensing terms that they haven't committed to licensing any version of their article under a licence inconsistent with the terms of our agreement – including the applicable embargo period – publication under the subscription model isn't suitable for authors whose funders require no embargo.

If you have any questions about our publishing options, costs, Open Access requirements, or our legal forms, please contact ASJournals@springernature.com

An online order form for reprints of your paper is available at https://www.nature.com/reprints/author-reprints.html. All co-authors, authors' institutions and authors' funding agencies can order reprints using the form appropriate to their geographical region.

We welcome the submission of potential cover material (including a short caption of around 40 words) related to your manuscript; suggestions should be sent to Nature Microbiology as electronic files (the image should be 300 dpi at 210 x 297 mm in either TIFF or JPEG format). Please note that such pictures should be selected more for their aesthetic appeal than for their scientific content, and that colour images work better than black and white or grayscale images. Please do not try to design a cover with the Nature Microbiology logo etc., and please do not submit composites of images related to your work. I am sure you will understand that we cannot make any promise as to whether any of your suggestions might be selected for the cover of the journal.

You can now use a single sign-on for all your accounts, view the status of all your manuscript submissions and reviews, access usage statistics for your published articles and download a record of your refereeing activity for the Nature journals.

To assist our authors in disseminating their research to the broader community, our SharedIt initiative provides you with a unique shareable link that will allow anyone (with or without a subscription) to read the published article. Recipients of the link with a subscription will also be able to download and print the PDF.

As soon as your article is published, you will receive an automated email with your shareable link.

With kind regards,

P.S. Click on the following link if you would like to recommend Nature Microbiology to your librarian
http://www.nature.com/subscriptions/recommend.html#forms

** Visit the Springer Nature Editorial and Publishing website at www.springernature.com/editorial-and-publishing-jobs for more information about our career opportunities. If you have any questions please click here.**

Open Access This Peer Review File is licensed under a Creative Commons Attribution 4.0 International License, which permits use, sharing, adaptation, distribution and reproduction in any medium or format, as long as you give appropriate credit to the original author(s) and the source, provide a link to the Creative Commons license, and indicate if changes were made.
In cases where reviewers are anonymous, credit should be given to 'Anonymous Referee' and the source.
The images or other third party material in this Peer Review File are included in the article's Creative Commons license, unless indicated otherwise in a credit line to the material. If material is not included in the article's Creative Commons license and your intended use is not permitted by statutory regulation or exceeds the permitted use, you will need to obtain permission directly from the copyright holder.
To view a copy of this license, visit https://creativecommons.org/licenses/by/4.0/

NMICROBIOL-25072607:

Response to the major comments of the reviewers

Reviewer 1

1. GF mice are **hyperphagic**. Is it possible that differences in food intake contributes to the changes the authors have observed? I wonder if a **2h fast is long enough**? I am also surprised by the **transit time** data. It is well recognized that germ free and conventional mice have different gut motilities and hence transit times. Further, **male and females** also show **differences in gut motility**. This has been widely reported. How do the authors account for this difference in their data?

REPLY

- In our view, it is unreasonable that **hyperphagy** in GF mice could contribute to their increased systemic (labelled) lipid uptake. A higher food uptake (due to hyperphagy) increases the systemic lipid levels, which should as consequence decrease the absorption of labelled lipids supplied per oral gavage to the mice. We observe the contrary, an increased systemic absorption of labelled lipids in GF mice.
- We have found no difference in uptake of labelled between **2h fasting** and 4-6h fasting in SPF mice, associated data can be provided to the reviewer.
- In our experimental set-up, **transit times** in GF vs. OMM^{12} vs. SPF were similar (**Figure S2A**):

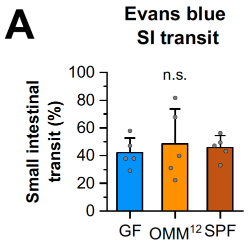

Figure S2A

- To test if transit times are sex-dependent, we will determine those in male and female GF, OMM^{12} and SPF mice (with an identical experiment as performed for the data shown in **Figure S2A**).

2. There are important contributions to bile acid metabolism from the nuclear receptor **FXR** and **bacterial enzymes like bile salt hydrolase**. How are these key host and microbial factors impacting this mechanism? This seems important since GF mice lack any activity and will also have significantly different FXR activity (among many other nuclear receptors like **CAR** which they did not follow up on). Further the BSH activity in OMM12 vs conventional mice is also significantly different and hence could be a driving factor in the bile acid pools.

REPLY

- The expression **FXR**, and **CAR** (including target genes) in liver will be investigated by a re-analysis of existing proteomic data and by additional mRNA analyses in GF, OMM^{12}, SPF mice.
- **BSH** cleaves the amid bond linking primary bile acids to taurine: TβMCA => βMCA; TCA => CA.

  We have previously published, that three strains of the OMM^{12} bacteria have a BSH activity (PMID: 33382950). We have not yet tested BSH activity of the SPF microbiota, but we assume, that it contains more BSH-positive bacteria due to a higher diversity. However, it`s difficult to directly compare the BSH activities between the OMM^{12} and SPF microbiota, since the few BSH-positive OMM^{12} strains, if dominant, could probably reach a similar biomass/cell numbers as the BSH-positive SPF strains. Hence, BSH activity must not necessarily be different between OMM^{12} and SPF bacteria.

- Most importantly, since we found **similar total levels of tauro-conjugated in biliary bile acids** in GF, OMM^{12} and SPF mice (**Figure I**), we can conclude, that the differential levels of TCA and TβMCA observed between GF, OMM^{12} and GF mice are not due to differential BSH activities (**Figure 6A**).

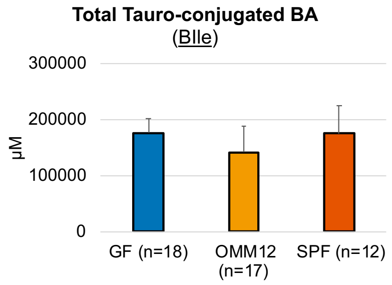

Figure I (new)

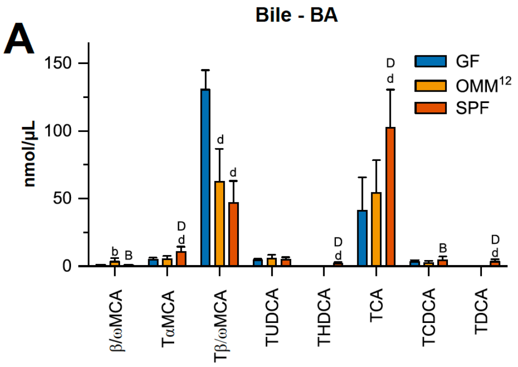

Figure 6A

3. Based on the methods, it appears that the authors used a combination of male and female mice. I certainly appreciate challenges due to cost, breeding, etc but how balanced are the various groups?

The figures should be **labeled such that the male and female datapoints** are clearly visible. Also it seems appropriate to **assess statistically if there are sex-specific differences**.

REPLY

- We will add the requested details and analyze the data **sex-specific** as well as display sex in the Figures as suggested.

Reviewer 2:

1. The **methods are too briefly written**, not allowing us to know what has been done and if the experiments are correctly designed. For example, for GC-MS, we cannot know if they analyze only free fatty acids or they include esterified ones. How the derivatization was done (or if it was done because there is really nothing explained, unless I missed a supplemental material) is also unclear. Similarly, there is little clue about how lipidomics was done.

REPLY

- All experimental details will be provided in Methods section.

2. The authors imply that **Akkermansia muciniphila** is implicated in the inhibition of lipid uptake. This is concluded from a comparison of OMM11 and OMM12, but the evidence is weak. In Figure 4B-D, the difference between OMM11 and OMM12 is not statistically significant, and the individual plots give the impression that the difference, if any, is very small. Even if there was statistical significance, a comparison of the averages would suggest that the contribution of this single strain is minor. **Consider removing the data, or add more evidence**.

REPLY

- Additional evidence for an involvement of **A. muc.** is available from an unpublished experiment, where mice were colonized with [Bacteroides thetaiotaomicron, Escherichia coli, and Eubacterium rectale] +/- A.muc. prior to oral gavage with labeled lipids for 1h.
  Those support an involvement of Akk. m. in intestinal absorption and systemic uptake of lipids: The levels of FA 16:0 [D5] and FA 16:0 [D31] are lower in plasma samples of mice colonized with [Bacteroides thetaiotaomicron, Escherichia coli, and Eubacterium rectale] plus Akk. m. 1h after gavage, compared to [Bacteroides thetaiotaomicron, Escherichia coli, and Eubacterium rectale]-colonized mice without Akk.m. (**Figure II**). In agreement, in luminal content (duodenum), the levels of the labelled lipids are higher in mice colonized Akk.m (since less lipids were taken up in the circulation) compared to those without Akk.m.

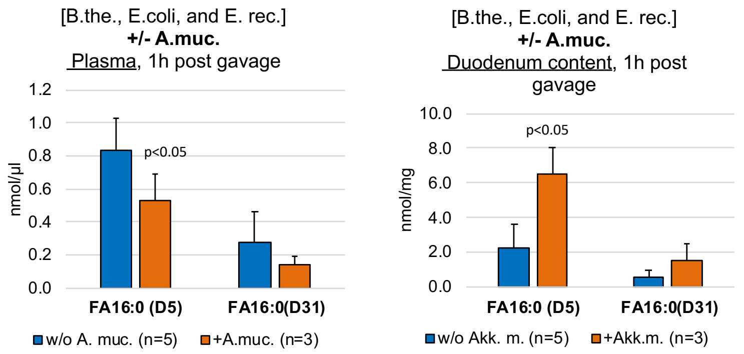

Figure II (new)

We suggest to add those data (as well as related data from this experiment), but still tone down the claims related to A.muc. in the abstract and results section ("A.muc., besides other microbial species, is involved in the regulation of intestinal lipid absorption" instead of "Specifically A.muc. lowers intestinal lipid absorption"); and discuss potential limitations of our experiments.

3. The data that suggest the implication of **Myd88** in microbiome-dependent lipid uptake regulation are weak. In Figure 7I, the change in **Cyp7b1 levels is very minor** compared to the drastic changes seen in Figure 7C. This shows that the role of Myd88 is minor and redundant. Similarly, the **changes in bile acids are small** in Figure 7JK and are not comparable with the changes in germ free versus SPF mice (Figure 6A). The changes in **phospholipase A1 activity are large (Figure 7L), but no changes in lysophosphatidylcholine are reported**, which makes me wondering why the experiment was not done. In other words, we see relatively large changes in phospholipase A1 activity without big changes in bile composition, which is against the claims made in Figure 6. Thus, without clarification, this could overturn the major claims of the study.

REPLY

- In our opinion, it is difficult to compare the **magnitudes of changes between the experiments** (Myd88 WT- Myd88 KO; GF-SPF), because we are working with mice that show naturally inter-individual differences, even if they have the same genotype and were housed identically.
- However, we understand the reviewer`s point and think that a reason for the "differences" might be, that the bile acid data in liver (referred to as rather "small" changes by the reviewer) are from a different mouse cohort (Cohort A), than the bile samples used for PLA assay (referred to as "large" changes by the reviewer) (Cohort B).
  We have liver samples from Cohort B available and will analyze those for bile acids, which might reduce the magnitudes of differences.
- Another reason for the differences might be, that we have mixed liver samples from male and female mice. In a preliminary re-analysis, we realized that bile acid levels and Cyp7b1 mRNA expression showed a remarkable sex-dependent difference. So, we will perform **a sex-specific analysis of bile acid levels** and **Cyp7b1** expression. This should also be in favor of Reviewer 1, who asked in general for sex-specific analyses.

- The missing **Lysophosphatidylcholine** levels are available and will be added to the manuscript.

4. The gut microbiome induces large changes in bile acids. This in itself should have an effect on lipid uptake, regardless of phosphatidylcholine levels. Without comparison of **germ free and SPF in a carboxyl ester lipase knockout background**, it is difficult to discriminate the contribution of bile acids and phosphatidylcholine. Of course, any other experiment that would help the discrimination can replace the one I suggested.

REPLY

- While the manuscript was in revision, we generated **CEL KO mice** (SPF and GF) and performed lipid uptake experiments (FA 16:0 [D5], FA 16:0 [D31]; 1h). We found that in CEL KO mice (SPF) 3-fold more labelled FA were taken up into plasma compared to CEL WT (SPF). The amounts of labelled lipids taken up in CEL KO (GF) mice were not significantly different to those determined in CEL KO (SPF) mice (**Figure III**).

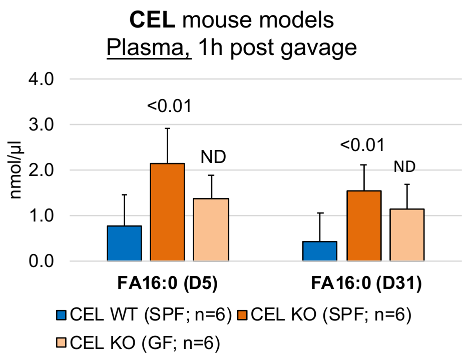

Figure III (new)

⇨ These results are a great advance for our manuscript, proofing the relevance of **CEL as PLA$_1$** in bile.
In **CEL WT SPF** animals, microbiome-activated CEL decreases biliary PC contents limiting intestinal and systemic lipid uptake (**Figure IV**), which is the main hypothesis of our paper. In the **absence of CEL in SPF mice**, less PC is degraded to LPC in bile, leading to a more efficient emulsification in gut lumen due to higher PC contents, and in turn, to higher lipid uptake. Hence, lipid uptake in **CEL KO GF** and **CEL KO SPF** mice is similar**.**

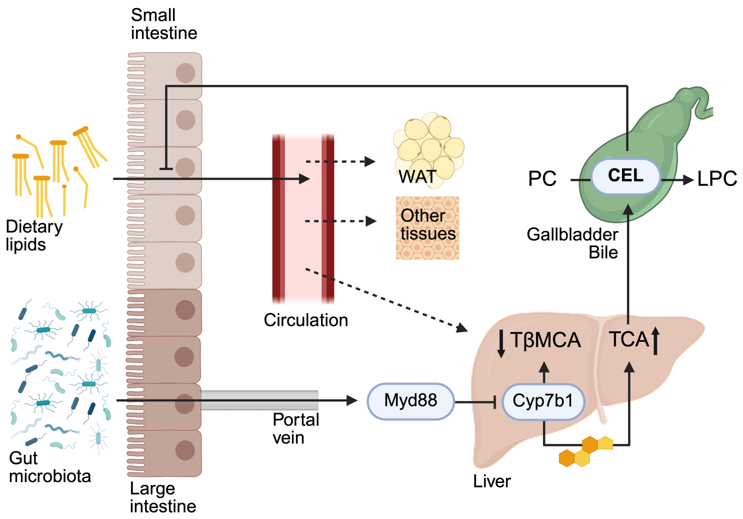

Figure IV (new)

These results as well as related data (mouse anatomies, lipid levels in tissues, etc.) will be included in the manuscript.

Reviewer 3:

1. The higher absorption observed in GF mice **contradicts previous reports** (**PMID: 37616368, 29649441**). Please provide some **explanation**. Whether the **housing conditions** for GF, OMM12 and SPF mice in this study identical? Was there no difference in **body weight** among the three groups? If the GF mice were significantly lighter, an identical lipid gavage would yield a higher dose-to-weight ratio and could potentially exaggerating absorption. How do the authors explain lower body weight of age-matched GF versus SPF mice? Moreover, several groups have reported **reduced intestinal lipid absorption after antibiotic treatment**; how do the authors interpret?

REPLY

- **PMID: 37616368**: This study investigates mice fed a high fat diet, which basically overloads all organs with lipids and significantly changes whole-body metabolism. Moreover, lipid absorption was “evaluated” from the determination of total (unlabeled) lipid contents in intestinal epithelial cells and fecal neutral lipids. We have applied the stable isotope labelling strategy in chow-fed mice to specifically determine lipid uptake/flux (which is the golden standard for metabolic analyses).
- **PMID 29649441**: Here, the authors injected a lipoprotein lipase (LPL) inhibitor in mice, prior to the gavage of radio-labelled lipids blocking peripheral uptake of lipids. As illustrated in **Figure V** (from PMID 29649441), the gavaged radiolabeled lipids accumulate over time (7h), because they cannot further be transported to peripheral tissues, which is un-physiological.

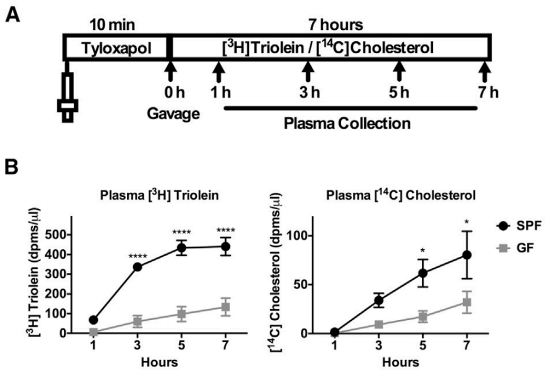

Figure V (from PMID: 29649441)

We aimed to develop and apply an experimental strategy allowing the investigation of intestinal and systemic lipid uptake in a physiological and "real life" context. Our strategy allows tracing whole-body flux of the labelled lipids. Therefore, as expected, the labelled lipids peak 1-2h h after gavage in plasma/bloodstream (**Figure 2C-D**), before transport to peripheral tissues like liver and adipose tissue.

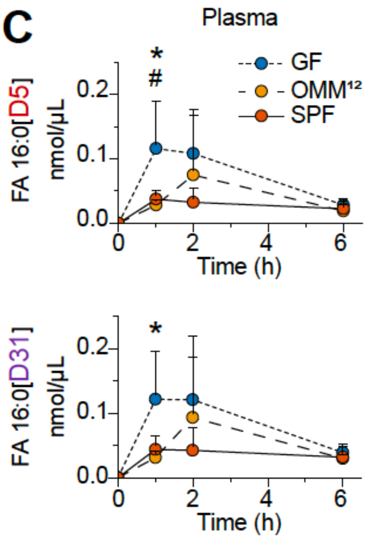

Figure 2C-D

- **Body weight** of our mouse models will be reported.
- Antibiotic treatments have a many side effects, including the disruption/change of some lipid metabolic processes. Therefore, is difficult to compare, results from studies using antibiotics with our findings.

⇨ **Most importantly**, our study includes **OMM12** mice housed identically to GF mice. All parameters measured in OMM12 are between those of SPF and GF, verifying the relevance of the microbiome. That's a major difference and advance compared to other published studies investigating the microbiome`s effect on host metabolism.

2. The conclusion of the article states that "the gut microbiota limits fatty acid absorption from the gut lumen into intestinal tissue." However, in **Figure 1B and 1E**, the levels of **FA16:0[D5] and FA16:0[D31] in the ileum tissue of SPF mice and OMM12 mice are higher than those in GF mice**. Additionally, in Figure 1B, no reduction in lipids is observed in the duodenum tissue—the primary site of lipid absorption—in SPF and OMM12 mice compared to GF mice. Does this contradict the conclusion of the article?

REPLY

- That's a misinterpretation that can be clarified. Lipids are already in bloodstream/plasma after 1h, therefore the amount determined in intestinal lumen and tissue has to be lower. To investigate specifically lipid uptake in intestinal tissue, shorter timepoints than 1h should must be chosen (e.g. 15 min). An explanation/clarification will be added in the results section.

3. Studies have **reported lower intestinal CD36 expression in GF than in SPF mice** (PMID: 28860383), yet Figure 3A shows the opposite trend. **Please increase sample size and add Western blotting analysis** to strengthen this conclusion. Studies have shown that the gut microbiota, such as Lactobacillus, can influence the **palmitoylation of CD36** (PMID: 40767021). Additionally, the bile acid receptor TGR5 (for which TCA is a weak agonist) can also affect the **palmitoylation and membrane localization of CD36** (PMID: 38698281). Therefore, **the role of CD36 in the gut microbiota-lipid absorption axis requires further discussion**.

REPLY

- **CD36 mRNA** will be analyzed in more replicates of intestinal tissue from GF, OMM^{12} and SPF mice.
- **CD36 protein** expression will be analyzed with Western blots in intestinal tissue from GF, OMM^{12} and SPF mice.
- A possible **palmitoylation of CD36** (reported in PMID: 40767021) will be investigated by mRNA expression analysis of the palmitoyl acyltransferases DHHC4 and DHCC5 (mediating CD36 palmitoylation) in intestinal tissue samples from GF, OMM12 and SPF mice.

4. The authors used physiology-based kinetic modelling to attribute the differences among the three tracers to "a single reduced absorption coefficient **k_abs**." Please corroborate this conclusion with independent experiments **such as a lymph-fistula model** or **a dual-isotope intestinal-flux assay**.

REPLY

- We think, that this suggestion is a "little bit too much" and that a validation of k_abs is not necessary, since it describes only very small component of the whole manuscript. Additionally, the suggested lymph-fistula model or a dual-isotope intestinal-flux assay are unrealistic to perform. They would require a specific request of an approval for animal experiments by the local authorities, which takes at least 6-9 months in Germany; as well as a practical experience in exactly these techniques, which we do not have.

- However, we will try to support the k_abs values by a re-analysis of the existing data with a different (e.g. bioinformatic) strategy.

5. Figure 3 only presents the relative composition 6 h post-gavage. Please provide **absolute concentrations of lipids** from GF, OMM12 and SPF mice, with statistical comparisons.

REPLY

- Will be done as suggested.

6. Tβ/ωMCA concentrations are negatively correlated with LPC levels, and the amounts of some unconjugated bile acids (e.g., CA) may also differ markedly among the mouse groups. How to determine which metabolite has the greatest impact on lipid absorption. Human bile is dominated by **glycine-conjugated CA and CDCA**. **Could other bile acids modulate CEL hydrolytic activity**?

REPLY

- The effect **of glycine-conjugated CA and CDCA** can be tested by performing PLA1 assays with mouse bile stimulated +/- CA and CDCA (similar to the TCA stimulation experiments shown in Figure 6C).

7. An **in vitro pure enzyme reaction demonstrating that CEL degrades PC to form LPC** is required to confirm that CEL is the enzyme responsible for PC degradation in bile. Additionally, although the expression level of CEL remains unchanged in GF, OMM12, and SPF mice, how is its activity regulated? Further in vitro pure enzyme reactions should be supplemented to investigate whether TCA promotes the degradation of PC to LPC by CEL.

REPLY

Most points of the comment have already been discussed in the manuscript. However, we will add some additional explanations for further clarification.

- Others have already published that CEL degrades PC to LPC (**PMID: 9148961**), which will be highlighted in the discussion.
  [Hence, in our opinion, an additional validation is not necessary. However, we could determine PC to LPC degradation by CEL performing PLA assays with bile from CEL WT (SPF) and CEL KO (SPF) mice.]
- The finding that CEL`s expression is unchanged between GF, OMM^{12}, and SPF mice (Figure 5E) indicates that it must be regulated **post-translationally**. It is very well known, that CEL is regulated by bile acids, particularly TCA [CEL= previously known as bile salt-dependent lipase (BSDL)], as explained in the discussion (PMIDs: 12454261, 21081507, 9331420)

8. The authors **could** supplement the study with **WT/MYD88-/- mice under different gut microbiota conditions (GF/SPF)** to further elucidate the role of the MYD88-CYP7B1 signaling pathway in mediating the effects of gut microbiota on lipid absorption.

REPLY

- Actually, we would have Myd88 KO mice in breeding. But, we think that the suggested experiments are not necessarily productive and misleading, since Myd88 regulates various processes including immunological reactions and lipid synthesis, and not only Cyp7b1. Absorption of dietary lipids, *de novo* lipid synthesis and metabolism are closely related and balanced. Consequently, we cannot expect e.g. a comparable uptake of labelled lipids in Myd88 KO mice (SPF) and GF WT mice.
- To support a Myd88-dependent regulation of hepatocyte bile acid metabolism (which seems to be most important point for the reviewer), we suggest to knock down Myd88 in HepG2 cells (hepatocyte cell line) using RNAi, prior to analysis of Cyp7b1 expression and bile acid profiles.

Point to point response to the referees (NMICROBIOL-25072607)

Thank you very much for reviewing the paper, your valuable comments and interest in our work. Your input greatly helped us to improve the manuscript. We tried our best to include the most important data from a lot of new experiments in the paper without overloading it.

Please find in the following our response to your comments.

All major changes are marked in red in the manuscript.

Reviewer 1

The authors describe a link between lipid absorption and the gut microbiota. They propose a connection between myd88, PLA1, and bile acid metabolism. I think the labeling experiments are well done and add key quantitative insight into their manuscript. However, the mechanistic insight is limited. For example, what factor(s) from the gut microbiota drive these changes? They have provided evidence that the gut microbiota is important but no evidence about how the gut microbiota is actually driving this change.

MAJOR COMMENTS

[1] GF mice are hyperphagic. Is it possible that differences in food intake contributes to the changes the authors have observed? I wonder if a 2h fast is long enough? I am also surprised by the transit time data. It is well recognized that germ free and conventional mice have different gut motilities and hence transit times. Further, male and females also show differences in gut motility. This has been widely reported. How do the authors account for this difference in their data?

REPLY: Food intake and energy absorption of male, adult age-matched GF, OMM12 and SPF mice with a C57BL/6J genetic background on a standard chow diet (as used in our study) were compared and reported previously (PMID: 36126044). GF animals ingest on average 15% more standard chow/day compared to OligoMM^{12} and SPF mice.

	Daily food intake (g)	
	Mean	SD
GF (n=24)	3.81	0.54
OMM12 (n=19)	3.29	0.49
SPF (n=10)	3.30	0.52

We think that the (minor) hyperphagy observed in GF mice has no influence on the experimental data and the results of our study, because:

(A) Oral gavage used to administer the labelled lipids the animals secures that the lipid mix enters the stomach and small intestine at a defined starting point, independently of the mouse`s food intake. The labelled lipids supplied by oral gavage “must” be absorbed in the small intestinal epithelium once they enter the intestinal lumen.

(B) The mice had no access to food two hours before and thus, a comparable baseline.

(C) We would expect that a 15% higher daily food intake in GF animals, elevating general systemic lipid levels, would rather decrease the absorption of labelled FA supplied by oral gavage. We observed the opposite.

(D) Application of a “correction factor” of ≈15% to the measured levels of labelled FA in GF mice, in one or the other direction, would not change the main result and outcome of the labeling studies, i.e. that the systemic uptake decreases from GF>OMM^{12}>SPF.

We chose “only” 2h of fasting, since gastric emptying occurs primarily during the first 30-60 minutes after meal ingestion in mice (PMID: 36371706). Further, we wanted to keep the deprivation period as short as possible as requested by the legal and regulatory requirements for animal experiments in Germany with the main aim to achieve standardized basal conditions before oral gavage. And, according to our experience, the variations of measured lipids between the individual animals per group were low enough indicating that 2h fasting is sufficient. An additional advantage of the short fast period is that the animal stress is kept as low as possible and interferes only minimally with physiology and behavior of the animals. Certainly, it is also important that the total duration of the animal experiments (fast-oral gavage-sampling = ∑8h for the 6h timepoint per mouse) allows a practicable execution, is doable within a reasonable timeframe by the experimenter, respectively, to yield robust and comparable results.

To the best of our knowledge, there are currently no accurate published transit time data of GF mice compared to colonized mice fed with a standard (chow) diet available (although often communicated). Therefore, we have repeated the experiment indicated by the reviewer. For repetition, we have included more replicates per group and to test if transit times are sex-dependent, male and female GF, OMM^{12} and SPF mice. We could confirm our previous results: The transit times of the gavaged lipid mix are not different between GF, OMM^{12}, SPF animals and are independent of sex.

The new data are shown in **Figure 2F** and commented in the RESULTS:

The body mass between GF, OMM^{12} and SPF mice fed with a standard chow diet is not significantly different [33].

RESULTS, Lines 418-420

Transit times of the lipid mix supplied per oral gavage are similar in the small intestine of male and female GF, OMM^{12} and SPF mice (**Figure 2F**).

RESULTS, Lines 467-468

---

[2] There are important contributions to bile acid metabolism from the nuclear receptor FXR and bacterial enzymes like bile salt hydrolase. How are these key host and microbial factors impacting this mechanism? This seems important since GF mice lack any activity and will also have significantly different FXR activity (among many other nuclear receptors like CAR which they did not follow up on). Further the BSH activity in OMM12 vs conventional mice is also significantly different and hence could be a driving factor in the bile acid pools.

REPLY: Bacterial BSH cleaves the amid bond linking primary bile acids to taurine and glycine. Most relevant for this study are the of conversions TβMCA (=> βMCA) and TCA (=> CA). We have previously reported, that three strains of the OMM^{12} bacteria have a BSH activity (PMID: 33382950). BSH activity of the SPF microbiota was not yet tested, but we assume, that it could more BSH-positive bacteria due to a higher diversity. However, it`s difficult to directly compare the BSH activities between the OMM^{12} and SPF microbiota, since the few BSH-positive OMM^{12} strains, if dominant, could probably reach a similar biomass/cell numbers as the BSH-positive SPF strains. Therefore, BSH activity must not necessarily be different between OMM^{12} and SPF bacteria.

To determine BSH activity for deconjugation of TβMCA and TCA by the gut microbiota of OMM^{12} and SPF mice, caecal contents were incubated with TβMCA and TCA for 0, 30 and 90 min revealing similar βMCA and CA levels, deconjugation capacities, respectively. BSH activity for TβMCA and TCA in GF mice was identical as when incubated with PBS (negative control). Hence, we conclude that concomitant increase of the TCA to TβMCA ratio in GF>OMM12>SPF (Figure 7G) cannot be related with bacterial BSH.

The new data are shown in **Figure 7F** and commented in the RESULTS:

Bacterial bile salt hydrolyzes (BSH) cleaves the amide bond linking BA to taurine, deconjugating TβMCA to βMCA and TCA to CA, respectively. To test BSH activity in the gut microbiota of OMM^{12} and SPF mice, caecal contents were incubated with TβMCA and TCA for 30 and 90 min revealing similar βMCA and CA levels and thus, deconjugation capacities at both timepoints (**Figure 7F**). BSH activity for TβMCA and TCA in GF mice was identical as in the incubations with PBS (negative control). Hence, we conclude that concomitant increase of the TCA to TβMCA ratio in GF>OMM^{12}>SPF is not related to bacterial BSH. Instead, the major driver is the downregulation of Cyp7b1 associated with gut microbial colonization.
RESULTS, Lines 606-613

In the intestine, FXR is activated by the presence of the gut microbiota of mice (PMID: 23395169, PMID 33568795), while in the liver, the gut microbiota was reported to have no effect on expression of FXR or its downstream targets like small heterodimer partner (SHP) (PMID: 23395169). However, if activated in liver, FXR promotes mRNA and protein expression of SHP. In turn, SHP represses the transcription of Cyp8a1 and Cyp7a1, which decreases the flux through the classic BA synthesis pathway (PMID: 38664391). Our results indicate the opposite, i.e. that the presence of the gut microbiota decreases the BA synthesis via the alternative pathway and thereby increase BA flux and synthesis via the classic pathway. Thus, we conclude that the differential levels of TβMCA and TCA determined in livers of GF, OMM[12] and SPF mice cannot be explained by a microbiome-dependent regulation of FXR.

Diindoles produced from the gut microbiota were shown to function as ligands for CAR (PMID: 38519460). Diindoles can up-regulate CAR and its target gene expression in primary human hepatocytes and mouse liver. In mice, CAR activation with synthetic CAR ligands, increase Cyp7a1 expression, but decreases TCA levels (by app. 50%) in liver (PMID: 28283178, PMID: 26984780). TβMCA concentrations remain unaltered. Our results are different with unchanged Cyp7a1 expression and an increase of TCA levels mice harboring gut microbiota (that according to PMID 38519460 could produce diindoles/CAR ligands). Hence, as for FXR, we conclude that the differential levels of TβMCA and TCA determined in livers of GF, OMM[12] and SPF mice also cannot be explained by a microbiome-dependent regulation of CAR.

These pathways/scenarios were explained in the DISCUSSION:

Other regulators of microbiome-dependent BA metabolism and FA absorption, might be farnesoid X repeptor (FXR), a well-investigated and key regulator of BA metabolism, and constitutive androstane repeptor (CAR) identified as potential candidate in our study. However, to the current knowledge FXR is only activated in the intestine by the gut microbiota, but not in the liver [68, 69]. If activated in liver, FXR induces small heterodimer partner (SHP), which represses Cyp7a1 and Cyp8a1 and in turn, decreases the flux through the classic BA synthesis pathway [7]. CAR was just recently reported to be activated by diindoles produced by the gut microbiota in liver [70] and synthetic CAR ligands can increase Cyp7a1 expression and TCA levels in liver [71]. Our results are different and indicate rather the opposite, a decreased BA synthesis via the alternative pathway leading to an increased BA synthesis via the classic pathway, induced by the gut microbiota.

DISCUSSION, Lines 699-708

---

[3] Based on the methods, it appears that the authors used a combination of male and female mice. I certainly appreciate challenges due to cost, breeding, etc but how balanced are the various groups? The figures should be labeled such that the male and female datapoints are clearly visible. Also, it seems appropriate to assess statistically if there are sex-specific differences.

REPLY: Indeed, the reasons for using a combination of male and female mice were due to challenges outlined by the reviewer, but certainly also to follow the 3R guidelines for animal experiments and prevent an un-necessary killing of female mice. We tried to balance the groups as good as possible. However, while that was achievable for plasma and liver samples, we have to admit that it was not possible for the collection of bile from SPF mice, because the sampling of gallbladder bile is per se very challenging plus the amount of bile stored in the gallbladder varies between the individual mice (also within the groups). As consequence, we had to use all bile samples that we could get independent of sex (Bile: GF - 8F,7M; OMM^{12} – 10F,1M; SPF - 10F,1M). We could not identify any sex specific differences related to BA or lipids measured in plasma, bile and tissues.

As suggested, in all main figures, female and male datapoints are indicated ("+" for female, "•" for male).

When and how sex-balanced groups were, was explained in the METHODS:

Whenever male and female animals were mixed, we tried to balance the groups as good as possible. However, while this was achievable for plasma and tissue samples, it was not possible for the collection of bile, because the sampling of gallbladder bile is per se very challenging plus the amount of bile stored in the gallbladder varied between the individual mice. We could not identify any sex specific differences related to BA or lipids measured in plasma, bile and tissues. Males are indicated with "•" and females with "+" in the main figures.
METHODS, Lines 119-124

MINOR COMMENTS

1. How and when was the bile collected?

REPLY: The bile was collected directly from the gallbladder using an insulin syringe and immediately frozen in liquid nitrogen, stored at -80°C.

This was explained in the METHODS:

Bile was collected directly from the gallbladder using an insulin syringe, immediately snap frozen in liquid nitrogen and stored at –80 °C.
METHODS, Lines 111-113

---

2. Sample size in some cases seems to be an issue. For example, Figure 3D. What explains the significant variation in the GF mice? Is sex specific?

REPLY: We have increased the sample sizes per group whenever possible for all analyses, including for the GF group (to n=26) shown in Figure 3C-G (previously Figure 3D). We cannot explain the significant variation in this group. The distributions of data points for male GF and female GF groups are similar.

Reviewer 2

The study of Plagge et al reports that the gut microbiota decreases lipid absorption by inducing a phospholipase activity in bile that reduces glycerophospholipids required for emulsification. The study provides evidence that overlooked enzymatic reactions in the bile are regulators of lipid absorption, making it a potential therapeutic target. The authors also provide data about a potential strain in the microbiome that contributes to the phenotype, as well as signaling pathways implicated. However, the data seems to show only weak effects on these aspects.

Strong points of the study:
The implication of lipid metabolic reactions within the bile, rather than in the intestinal lumen, is very interesting.
The authors perform extensive proteomics and enzymology to provide convincing mechanistic explanations for bile lipid changes.

MAJOR COMMENTS

[1] The methods are too briefly written, not allowing us to know what has been done and if the experiments are correctly designed. For example, for GC-MS, we cannot know if they analyze only free fatty acids or they include esterified ones. How the derivatization was done (or if it was done because there is really nothing explained, unless I missed a supplemental material) is also unclear. Similarly, there is little clue about how lipidomics was done.

REPLY: We apologize for providing not enough details in the methods section, which has been completely revised including the GC-MS analysis of total fatty acids and lipidomic analyses of complex lipids.

---

[2] The authors imply that Akkermansia muciniphila is implicated in the inhibition of lipid uptake. This is concluded from a comparison of OMM11 and OMM12, but the evidence is weak. In Figure 4B-D, the difference between OMM11 and OMM12 is not statistically significant, and the individual plots give the impression that the difference, if any, is very small. Even if there was statistical significance, a comparison of the averages would suggest that the contribution of this single strain is minor. Consider removing the data, or add more evidence.

REPLY: We understand the reviewer`s criticism and admit, that a relevance of *A. muciniphila* is certainly not the key finding of our paper plus its contribution to systemic lipid absorption might be only minor. However, we think that it might be still worth mentioning, because *A. muciniphila* was previously

assumed to inhibit systemic lipid uptake and accumulation (PMID: 23671105, PMID: 31263284), which is per se consistent with our findings. Hence, we decided to tone down the claims related to *A. muciniphila*. (to: "*A.muciniphila* besides other microbial species contributes to intestinal lipid absorption") and add more evidence:

(i) The GF-OMM[11]-OMM[12] data were reanalyzed. We show now the absolute concentrations of FA16:0[D5] and FA16:0[D31] in Figure 4B-C (uniformly for all uptake experiments in the manuscript; before FA16:0[D5] and FA16:0[D31] were presented as % of total lipids for this experiment). In addition, the concentrations in liver and plasma were now tested for significance of difference between the groups of OMM[11] and OMM[12]. We found that the levels of FA taken up are significantly different between OMM[11] and OMM[12] in the liver ($p<0.05$), but not in plasma ($p=0.1$ and 0.07) (Figure 4B-C). The PC/LPC ratios in bile were found significantly different between OMM[11] and OMM[12] (Figure 4D).

(ii) Additional data from another experiment were added, where the uptake of FA16:0[D5] and FA16:0[D31] in plasma and liver of mice colonized with [Bacteroides thetaiotaomicron, Escherichia coli, and Eubacterium rectale] +/- *A.muciniphila* prior to oral gavage with labeled lipids for 1h was quantified. In agreement, with the OMM[11]-OMM[12] data, the levels of the labelled FA were lower when *A. muciniphila* was added to the EAM consortium. (Unfortunately, bile samples for measurements of PC/LPC ratios were not available from this experiment due to challenges/issues in bile collection from gallbladder.)

The reanalyzed and new data are shown in **Figure 4B-F** and commented in the RESULTS:

To support that *A. muciniphila* can affect systemic lipid absorption, the uptake of labelled lipids in plasma and liver of mice colonized with easy accessible microbiota (EAM) without or with *A.muciniphila* was tested. The EAM consortium consists of the three bacterial strains *Bacteroides thetaiotaomicron, Escherichia coli, and Eubacterium rectale*, representing the three most abundant bacterial phyla of human gut microbiota [13]. Addition of *A.muciniphila* to the EAM consortium decreased the amounts of FA16:0[D5] and FA16:0[D31] in plasma and liver one hour after oral gavage with tracers (**Figure 4E-F**) providing further evidence that *A.muciniphila* besides other microbial species contributes to lipid absorption.

RESULTS, Lines 505-512

---

[3] The data that suggest the implication of Myd88 in microbiome-dependent lipid uptake regulation are weak. In Figure 7I, the change in Cyp7b1 levels is very minor compared to the drastic changes seen in Figure 7C. This shows that the role of Myd88 is minor and redundant. Similarly, the changes in bile acids are small in Figure 7JK and are not comparable with the changes in germ free versus SPF mice (Figure 6A). The changes in phospholipase A1 activity are large (Figure 7L), but no changes in lysophosphatidylcholine are reported, which makes me wondering why the experiment was not done. In other words, we see relatively large changes in phospholipase A1 activity without big changes in bile

composition, which is against the claims made in Figure 6. Thus, without clarification, this could overturn the major claims of the study.

REPLY:

The LOG2 abundances of proteins in Figure 7C cannot directly be compared with the (un-logged) relative mRNA expression levels shown in Figure 8B (formerly Figure 7I). The common presentation of protein abundances in proteomics is as LOG2. We apologize for not having explained this well enough. Because, for the first version of our manuscript, we have mixed for the mRNA and BA analyses Myd88 KO and control mice from different experiments including female and male mice in some but not all groups, we reanalyzed the data and show only male mice from the same experiment. Still, the changes between Myd88 KO vs. Controls might be not as large as for the comparison GF vs. SPF, but the direction of changes is similar for 5 of the 6 major BA found in liver. We principally understand the reviewer`s point, but please also consider, that it is generally quite difficult to compare the magnitudes of changes between different animal experiments due to natural inter-individual differences, which are can even be present if from the same genotype and housed identically.

The requested LPC and PC/LPC ratios were included in Figures 8F-G and those are in line with the results from the PLA_1 assay. We apologize for not showing those before.

Additionally, to provide further evidence for a relevance of Myd88 for lipid absorption, we performed new and supporting experiments. Myd88 KO and control mice were supplement for 1 hour with FA16:0[D5]) and tripalmitin $(TG(16:0[D31])_3$ per oral gavage, before GC-MS analyses of FA16:0[D5] and FA16:0[D31] revealing higher levels in plasma of Myd88 KO mice compared to controls (similar to GF vs. SPF mice). To confirm a regulation of Cyp7b1 expression by Myd88, we knocked down Myd88 in the HepG2 hepatocyte cell line using RNAi. In cells treated with siRNAs against Myd88 the expression of Cyp7b1 was 3.5-fold increased (Figure 8I), which is in agreement with mRNA expression profiles detected in liver samples from Myd88 KO animals (Figure 8B).

We admit, that there might be additional signaling cascades and mechanisms that contribute to the differences in TβMCA and TCA found in liver of GF, OMM^{12} and SPF mice, but we think that together our data provide enough evidence for a major relevance of Myd88.

The reanalyzed and new data are shown in **Figure 8B-J** and commented in the RESULTS:

We could detect a 2-fold induced Cyp7b1 transcription (**Figure 8B**), reduced TCA, but increased TβMCA contents in livers of mice with a global gene-deficiency of Myd88 compared to those from control animals (**Figure 8C**) as well as shift towards classical BA synthesis indicated by the lowered TCA/TβMCA ratios (**Figure 8D**). TUDCA, TDCA and TDCA contents were reduced in livers Myd88 KO animals compared to controls, as observed for the GF vs. SPF comparison (**Figure 8C**). *RESULTS, Lines 626-630*

In agreement, total LPC levels were 2.3-fold increased, but PC/LPC ratios 2.8-fold decreased in Myd88 KO animals as well as the systemic uptake of FA16:0[D5] and FA16:0[D31] one hour after oral gavage

(**Figure 8F-H**) strengthening a relevance of Myd88 for lipid absorption. To confirm an involvement of Myd88 in regulation of Cyp7b1 expression, we finally knocked down Myd88 in the HepG2 hepatocyte cell line using RNAi (**Figure 8I-J**). These experiments revealed that in cells treated with siRNAs against Myd88, the mRNA expression of Cyp7b1 was 3.5-fold increased, which is in line with mRNA expression profiles detected in liver samples from Myd88 KO animals (**Figure 8B**).
RESULTS, Lines 633-640

---

[4] The gut microbiome induces large changes in bile acids. This in itself should have an effect on lipid uptake, regardless of phosphatidylcholine levels. Without comparison of germ free and SPF in a carboxyl ester lipase knockout background, it is difficult to discriminate the contribution of bile acids and phosphatidylcholine. Of course, any other experiment that would help the discrimination can replace the one I suggested.

REPLY: While the manuscript was under review, mice with a global CEL deficiency were bred. Investigation of PLA_1 activity in bile revealed a significant reduction when CEL was absent. In agreement, PC/LPC ratios were higher in CEL than in controls. One hour after oral gavage with the labelled lipid mix, the concentrations of [D5]- and [D31]-FA 16:0 were elevated in plasma and liver of SPF CEL KO mice compared to SPF wildtypes demonstrating that CEL is involved in lipid absorption. But, FA16:0[D5] and FA16:0[D31] levels were similar in GF CEL KO and SPF CEL KO mice demonstrating that the gut microbiota limits systemic lipid uptake via CEL. Together, these data confirm that CEL is involved in systemic lipid uptake regulated by the gut microbiota.

The new data are shown in **Figure 5F-I** and commented in the RESULTS and DISCUSSION:

To confirm an involvement of CEL in gut microbiota-dependent lipid absorption, mice with a global CEL deficiency were bred. Investigation of PLA1 activity in bile after incubation with PC15:0/18:1[D7] for 2 and 15 min revealed a significant reduction when CEL was absent (**Figure 5F**). The LPC18:1[D7] levels were 2.0-fold lower in CEL-deficient compared to control mice. Accordingly, PC/LPC ratios were 2.5-fold higher in CEL KOs than in controls (**Figure 5G**). Most importantly, one hour after oral gavage with FA16:0[D5] and $TG(16:0[D31])_3$, the concentrations of FA16:0[D5] and FA16:0[D31] were 2.5 to 5.0-fold elevated in plasma and liver in SPF CEL KO mice compared to SPF controls demonstrating that CEL is involved in lipid absorption(**Figure 5H-I**). The result that FA16:0[D5] and FA16:0[D31] levels were not significantly different in GF CEL KO and SPF CEL KO mice indicates that the gut microbiota limits systemic lipid absorption via CEL.
RESULTS, Lines 551-560

CEL deficiency decreases PLA_1 activity in bile and increases systemic lipid uptake in SPF mice.
RESULTS, Lines 563-564

In CEL-deficient mice housed in a SPF environment, PLA1 activity in bile was reduced and the lipid uptakes in plasma and liver increased, but similar to those determined in GF CEL KO mice demonstrating that CEL is involved in gut microbiota-dependent regulation of lipid absorption. *DISCUSSION, Lines 668-671*

MINOR COMMENTS

[5] I have the impression that the values in Figure 1B and 1C-F do not match. For example, in Figure 1C, we see no difference at 6H, but in Figure 1B it says that FA 16:0[D5] is half.

REPLY: We apologize for the confusion. The values shown in Figure 1B and 1C-F cannot be directly compared, as in 1B the fold-changes of the area under the curves (AUC) for all timepoints (0-6h) after gavage, while in 1C-F the time courses of the absolute concentrations at 1, 2 and 6h (equaling the flux kinetics) are shown.

To avoid confusion, we have added a statement to the FIGURE LEGENDS:

Note, when comparing Figure 1B with 1C-F, that in 1B the fold-changes of the area under AUCs for all timepoints (0-6 h) are presented, while in 1C-F the time courses of the absolute concentrations at 0, 1, 2 and 6 h are shown.
FIGURE LEGENDS, Lines 756-758

---

[6] I am not sure how useful the modeling in Figure 2 was. In Figure 2C, it seems that it does not predict very well many of the data. We do not see the full set of predictions in the supplements, and thus I am not convinced. The authors do not provide explanation on parameters that would provide the goodness of the initial model neither.

REPLY: We thank the reviewer for pointing out that the use of the modeling approach was not well explained. We now added Supplementary Figure 2 depicting the fits of the original and the extended model with k_abs parameter being group-specific to the experimental data, and provide the corresponding model fits and parameter estimates in Supplementary Data 1. We provide a more detailed description of the model and its parameters in materials and methods, and extended motivation and interpretation of the model results in the results section.
We note that while the basic model is not capturing every experimental data profile in every tissue with high accuracy, it is still a powerful tool to analyze which lipid absorption processes are changing in microbiota-specific way, that would explain the differences in fatty acid profiles in different tissues simultaneously. Therefore, we decided to propose a coarse-grain model that contains only 6 parameters that describe the major lipid metabolism processes, and test which of this parameter should be altered in a microbiota-specific way in order to explain the observed differences in fatty acid levels in several

tissues. While changing 3 out of 6 parameters resulted in a substantial reduction in Akaike information criterion (AIC), which assesses whether model fits the data better while penalizing for increasing complexity, the parameter k_abs responsible for intestinal absorption resulted in by far the largest decrease in AIC. These results indicate that among all processes, microbial effect on intestinal absorption (and not on luminal flow or other parameters) would result in differences between FA profiles in different tissues (plasma, liver, small intestine, iWAT, eWAT) between the mouse groups that are similar to the ones observed experimentally.

For a better explanation the model`s description was extended in the METHODS and RESULTS. In addition, the related **Supplemental Data 2** were extended and **Supplemental Figure 2** was added:

The multi-compartment kinetic model of fatty acid metabolism in the mouse contained 11 compartments (duodenum, jejunum, ileum and colon lumen, duodenum, jejunum, ileum and colon tissue, plasma, liver and fat tissue). One additional compartment (small_intestine_gi) was used as reservoir for the initial fatty acid abundance. The plasma compartment incorporated processes occurring in all other body parts apart from the gastrointestinal tract (GIT), liver and fat tissue. Labelled fatty acid administration was modelled as an input to the small_intestine_gi compartment of the initial amount of D. Label propagation through the body was driven by the flow of gastrointestinal material in different GIT sections and lumen:tissue and tissue:serum diffusion and absorption coefficients. Model parameters and equations are provided in **Supplemental Data 1**. All equations were defined for fatty acid amounts. Parameters for FA 16:0[D5] and FA 16:0[D31] were assumed to be the same and were fitted simultaneously to the data from both fatty acid measurements. For the parameter fitting, fatty acid concentrations were converted into amounts using estimated compartment volumes provided in **Supplemental Data 1**. The model was created using the MatLab 2023a SimBiology Toolbox (MathWorks). To assess model improvement when each of the model parameters is set to be mouse group-specific, a general model with six parameters was built, which included equations for FA 16:0[D5] and FA 16:0[D31] from each mouse group, and parameters were fitted to the data profiles from the three mouse groups simultaneously. Next, each of the six parameters were defined as mouse-group specific, and the equations containing this parameter for each mouse group were changed accordingly. The modified models thus contained eight parameters (since one of the original six parameters was represented as three mouse group-specific parameters). For each of the modified models, mean squared error, Akaike information criterion (AIC), and Bayesian information criterion (BIC) were recorded. The model with the largest negative change of the AIC was considered the best improvement compared to the general model.

METHODS, Lines 131-153

To analyze what processes involved in lipid flux are potentially affected by microbiota resulting in the observed differences in labelled FA levels across tissues, we decided to model lipid metabolism at the system level.
RESULTS, Lines 442-44

The advantage of using model selection with Akaike information criterion is that it provides a balance between model complexity and its goodness-of-fit, and helps to identify parameters which, when added to the model, improve its fitting to experimental data across several tissues simultaneously.
RESULTS, Lines 469-471

---

[7] How were bile samples stored? This could affect the production of lysophosphatidylcholine.

REPLY: Bile was collected directly from the gallbladder using an insulin syringe, immediately snap frozen in liquid nitrogen and stored at –80 °C to prevent production of LPC from PC.

<u>This was explained in the METHODS:</u>

Bile was collected directly from the gallbladder using an insulin syringe, immediately snap frozen in liquid nitrogen and stored at –80 °C.
METHODS, Lines 111-113

---

[8] The authors often use correlation plots (Figures 1EF, 6BG), but I believe they are often misused and are irrelevant for the conclusions. If two variables, for example TCA and LPC concentrations, change in the same direction between groups, it is quite normal that a positive correlation would appear. In that case, the positive correlation would just reflect the group effect, and does not necessarily show any causality between the variables. Plots would be relevant only if there is correlation even within groups, but with the small numbers that is often used in the study, this is hard to say. For example, in Figure 6B, the right panel suggests that TBMCA negatively correlates with LPC, but if we just look at OMM12 the correlation seems to be positive. Most of the plots can be simply replaced by different graphs that show the changes in variables separately, to show that they go on the same or opposite direction. This will be more correct without affecting the conclusions.

REPLY: It was not our intention to mislead the reader, but we understand the reviewer´s point. Therefore, we omitted all correlation plots from the paper. It was not necessary to replace them by the related barplots, since those were already in the manuscript before.

---

[9] The authors propose that phospholipase A1 activity is affected by bile acid levels, rather than the expression levels of enzymes. This would be correct only if TBMCA or TCDCA have no effect on phospholipase A1 activity. Please refer to appropriate studies to show if it's the case, or test it experimentally.

REPLY: We agree and think that specifically the effect of TβMCA on PLA_1 is of major relevance for our findings. Therefore, we tested it experimentally. We found that addition of TβMCA, whose levels are lower in GF compared to colonized mice, reduces PLA_1 activity when added to GF bile. These data provide further and important evidence for BA-dependent regulation of PLA_1 by the microbiome: Both, the elevated TCA and reduced TβMCA stimulate PLA1 activity in bile of SPF and OMM^{12} mice compared to GF animals. TCDCA, whose levels are reduced by trend in GF mice, was reported to stimulate CEL (PMID: 6799328).

The new data are shown in **Figure 5H-I** and commented in the RESULTS and DISCUSSION:

Of note, addition of TβMCA, whose levels are strongly lowered in bile of SPF and OMM^{12} mice (**Figure 6A**) reduced PLA1-mediated LPC18:1[D7] generation from PC15:0/18:1[D7] almost 2-fold (**Figure 6H**).
RESULTS, Lines 580-581

Together, these findings demonstrate, that in bile, PC to LPC conversion by PLA1 is stimulated by TCA and inhibited by TβMCA, whose levels depend on microbial colonization.
RESULTS, Lines 584-585

---

[10] The protein data in Figure 7C would be strengthened if the authors analyze mRNA levels of relevant targets too. It will show if the changes occur at the transcriptional level, and this would make the association with Figure 7I better, as the latter provides mRNA data.

REPLY: We totally agree with the reviewer`s suggestion. But, unfortunately, we do not have any remaining liver tissue from the mice that were used for this experiment, as liver tissues were used up for the proteomic measurements (also BA analyses and lipidomics, etc.). Showing mRNA expressions obtained from a different sample cohort might be suboptimal, since it will not allow a fair comparison of the mRNA expressions with the protein abundances.

---

[11] The comparison between deuterated fatty acid and deuterated triglyceride is clever, as it helps the discrimination between lipid digestion and lipid uptake. The authors explain this in the discussion. If the authors mention the rationale of this design earlier, for example early in the results, a reader would understand better why the experiment is done like this.

REPLY: As suggested, an explanation for the rationale of combining the deuterated fatty acid and deuterated triglyceride in the lipid mix was added at the very beginning of the RESULTS:

If the gut microbiota affects TG lipolysis in the gut lumen, different time-dependent concentrations profiles (1-6 h) of FA16:0[D5] and FA16:0[D31] (derived from TG(16:0[D31])$_3$) will be expected between GF, OMM[12] and SPF mice in blood plasma and tissues.
RESULTS, Lines 442-426

The results were almost identical for FA16:0[D5] and FA16:0[D31] indicating that TG lipolysis is not affected by the gut microbiota.
RESULTS, Lines 433-434

Reviewer 3

In this study, Johannes et al. systematically investigated how the gut microbiota influences host lipid absorption and the underlying molecular mechanisms. The authors propose that the microbiota suppresses hepatic Cyp7b1 activity, increases taurocholate synthesis, activates a phospholipase A1 in bile, accelerates phosphatidylcholine degradation, and ultimately inhibits intestinal lipid uptake. The work is methodologically rich, thereby uncovering a "microbiota–bile–lipid-absorption" axis. However, the causal evidence remains incomplete, and additional experiments are required for full validation.

MAJOR COMMENTS

[1] The higher absorption observed in GF mice contradicts previous reports (PMID: 37616368, 29649441). Please provide some explanation. Whether the housing conditions for GF, OMM12 and SPF mice in this study identical? Was there no difference in body weight among the three groups? If the GF mice were significantly lighter, an identical lipid gavage would yield a higher dose-to-weight ratio and could potentially exaggerating absorption. How do the authors explain lower body weight of age-matched GF versus SPF mice? Moreover, several groups have reported reduced intestinal lipid absorption after antibiotic treatment; how do the authors interpret?

REPLY: Both articles referenced by the reviewer (PMID: 37616368, PMID: 29649441) are excellent and valuable studies reporting important findings. However, when comparing those with ours, it is important to consider the different experimental conditions and analytical methods used. We have applied stable isotope-labelled lipids that can be accurately traced and quantified with mass spectrometry, which is currently the gold standard in flux analysis. Our experimental setup allows the investigation of lipid fluxes and metabolism as close to physiological conditions as possible, which was a major aim of the study.
Wang and colleagues (PMID: 37616368) reported that GF mice fed for 10 weeks with a high fat diet contained lower amounts of unsaturated FAs in their intestinal epithelial cells compared to those of conventional mice using a commercially available colorimetric assay based on the sulfo-phospho-vanillin method (PMID: 36261171). Saturated FA could not be detected with the used method.
Martinez-Guryn et a. (PMID: 29649441) analyzed triglyceride (TG) accumulation in plasma after injecting GF and SPF mice a lipoprotein lipase (LPL) inhibitor (tyloxapol), prior to gavage of radioactive-labelled [^{3}H] TG, and revealed a higher accumulation of [^{3}H] over 7h in plasma of SPF mice compared to GF animals on a chow and high fat diet (PMID: 29649441; see Figure 2A-B below, for chow diet). Tyloxapol blocks peripheral lipid uptake and interferes with whole body lipid flux and transport. Hence, lipids accumulate over time in plasma. However, the authors also tested lipid accumulation in GF and SPF mice without prior application of tyloxapol, which revealed similar uptake kinetics in plasma as when tyloxapol was used (Figure 2D-E below, for chow diet). This is odd and very surprising, since

plasma lipid levels should decrease after the first hours due to flux in peripheral tissues (as observed in our study, Figure 1C of our manuscript), and not accumulate over the time.

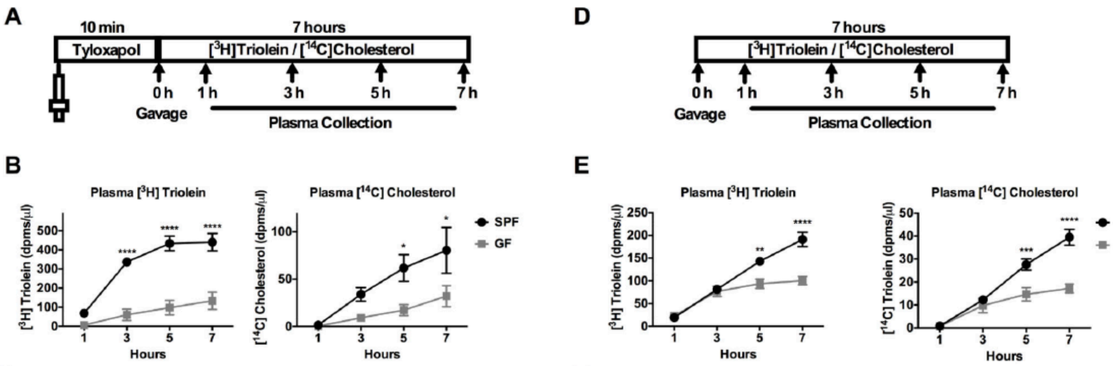

Figure 2A-B, D-E from PMID: 29649441

Very recently, Gao et al., showed that gut microbiota-induced interleukin-22 production in small intestinal T-cells reduces absorption of [^{3}H] -labelled TG in enterocytes that can be restored by the application of broad-spectrum antibiotics/dysbiosis (PMID: 41066556).

However, in our opinion, it is also difficult to compare our results with those obtained using antibiotics to disrupt the microbiome. Antibiotics have side effects, including alterations of lipid metabolic processes. Also, the effect of the antibiotics on the microbiome essentially depends on the type and duration. During antibiotic treatment the total bacterial load in the gut is severely reduced, particularly during the first days of treatment, but recovers after one to five weeks and can even overreach baseline levels (before antibiotic treatment) in mice and humans (PMID: 24748167, PMID: 22185696).

We have added some explanation related to the studies discussed above in the DISCUSSION:

A major aim our study was to investigate lipid flux and metabolism as close to physiological conditions as possible. Therefore, we applied stable isotope-labelled lipids that can be accurately traced and quantified with mass spectrometry, the gold standard in flux analysis. When comparing our results with those obtained in other studies, it is important to consider the individual experimental and methodical conditions. Wang and colleagues reported that GF mice fed for 10 weeks with a high fat diet contained lower amounts of unsaturated FAs in their intestinal epithelial cells compared to those of conventional mice using a colorimetric assay based on the sulfo-phospho-vanillin method [56]. Application of lipoprotein lipase (LPL) inhibitor, which blocks peripheral lipid uptake, prior to gavage of [^{3}H] -labelled TG, revealed a higher accumulation of [^{3}H] over 7h in plasma of SPF compared to GF on a chow and high fat diet [9]. Just recently Gao et al., showed that gut microbiota-induced interleukin-22 production in small intestinal T-cells reduces absorption of [^{3}H] -labelled TG in enterocytes of mice fed a standard diet, which can be restored after treatment with broad-spectrum antibiotics [57, 58].
DISCUSSION, Lines 652-663

Details of the housing conditions were described in the methods section. Briefly, GF and gnotobiotic animals (OMM[12], OMM[11]) were bred in plastic film isolators, SPF mice in individually ventilated cages; located in rooms with a controlled environment (20–22 °C, 50–55 % humidity) and 12 h light/dark cycles. GF, gnotobiotic and SPF mice received pelleted 50 kGy gamma-irradiated feed (V1124-927, Ssniff Spezialdiäten GmbH, Germany) and autoclaved water ad libitum.

The body mass of GF, OMM[12] and SPF mice was not significantly different, as reported previously (PMID: 36126044).

This was added to the results section:

The body mass between GF, OMM[12] and SPF mice fed with a standard chow diet is not significantly different [33].
RESULTS, Lines 418-420

---

[2] The conclusion of the article states that "the gut microbiota limits fatty acid absorption from the gut lumen into intestinal tissue." However, in Figure 1B and 1E, the levels of FA16:0[D5] and FA16:0[D31] in the ileum tissue of SPF mice and OMM12 mice are higher than those in GF mice. Additionally, in Figure 1B, no reduction in lipids is observed in the duodenum tissue—the primary site of lipid absorption—in SPF and OMM12 mice compared to GF mice. Does this contradict the conclusion of the article?

REPLY: We apologize for the confusion and not having explained the kinetics of the labelling approach well enough. The values shown in Figure 1B and 1C-F cannot be directly compared, as in 1B the fold-changes of the area under the curves (AUC) for all timepoints (0-6h) after gavage, while in 1C-F the time courses of the absolute concentrations per timepoint (equaling the flux kinetics) are shown.

The basic flux kinetics in our experiments are as follows:

I. In the first 20 min the labelled lipids reach approximately the half of the jejunum.
II. After on hour the labelled lipids can be detected in colon tissue and they peak in blood plasma.
III. Peripheral tissues including liver and adipose tissues are reached at 1-2h and six hours.

Therefore, the levels of FA detected at 1h are ideal to estimate the uptake in the circulation (and liver), but not for the flux in intestinal tissues. For intestinal absorption, the 1h timepoint is basically “already too late”.
The AUCs and levels of FA16:0[D5] and FA16:0[D31] (Figure 1B and 1E) in the ileum tissue of SPF mice and OMM[12] mice are higher than those in GF, because less amounts of FA16:0[D5] and

FA16:0[D31] were taken up in plasma of OMM^{12} and SPF compared to GF mice. Thus, these results do not contradict the conclusion of the article.

To avoid confusion the flux kinetics and the related findings were explained in the RESULTS:

The basic flux kinetics are: In the first 20 min the lipid mix reaches the half of the jejunum. After one hour the labelled lipids can be detected in colon tissue and they peak in blood plasma, before peripheral tissues including liver at two hours and white adipose tissues at six hours are reached. If the gut microbiota affects TG lipolysis in the gut lumen, different time-dependent concentrations profiles (1-6 h) of FA16:0[D5] and FA16:0[D31] (derived from $TG(16:0[D31])_3$) will be expected between GF, OMM^{12} and SPF mice in blood plasma and tissues.
RESULTS, Lines 421-426

Correspondingly, in ileum and colon contents, the AUCs of both labelled FA were much larger in colonized compared to GF animals (log2FC between 1.8 and 3.6, **Figure 1B, E-F**), because less labelled FA reached the circulation in SPF and OMM^{12} compared to GF mice. The results were almost identical for FA16:0[D5] and FA16:0[D31] indicating that TG lipolysis is not affected by the gut microbiota.
RESULTS, Lines 430-434

To assess absorption in intestinal tissues ($=k_{Abs}$) we have applied the physiology-based kinetic modelling (Figure 2). To experimentally confirm k_{Abs} estimated with the modeling approach, we have now added an experiment, in that GF, OMM^{12} and SPF mice were supplied with labelled FA for only 20 minutes allowing an analysis of absorption in duodenum tissue/intestinal absorption. (See also response to Major Comment 4).

These new data are shown in **Figure 2E** and commented in the RESULTS:

This is supported by the finding that 20 minutes after oral gavage, in duodenum tissue, 2.4-3.2 % of total FA 16:0 originated from absorption (FA16:0[D31] and FA 16:0[D5]), while in OMM^{12} and SPF mice only 1.3-1.6% and 1.0-1.4% (**Figure 2E**).
RESULTS, Lines 463-465

---

[3] Studies have reported lower intestinal CD36 expression in GF than in SPF mice (PMID: 28860383), yet Figure 3A shows the opposite trend. Please increase sample size and add Western blotting analysis to strengthen this conclusion. Studies have shown that the gut microbiota, such as Lactobacillus, can influence the palmitoylation of CD36 (PMID: 40767021). Additionally, the bile acid receptor TGR5 (for which TCA is a weak agonist) can also affect the palmitoylation and membrane localization of CD36

(PMID: 38698281). Therefore, the role of CD36 in the gut microbiota-lipid absorption axis requires further discussion.

REPLY: As suggested, we have increased the sample size for the mRNA analyses in small intestinal tissues of GF, OMM12 and SPF mice to n=19-28/group. In addition to CD36, we have measured in all samples the mRNA expressions of DHHC4 and DHHC5, that palmitoylate CD36 (PMID: 40767021, PMID: 38698281); and FATP4. The reference PMID: 28860383 indicated by the reviewer shows a lower expression of CD36 in intestinal epithelial cells isolated from ileum of GF mice compared to conventional mice. However, also in our extended sample set, no difference in CD36 mRNA expression between the groups in duodenum, ileum and jejunum tissue was observed (Figure 3A). Also, no major differences in small intestinal DHHC4 and 5 mRNA expressions between GF and OMM^{12} or SPF animals could be detected suggesting no differences in CD36 palmitoylation.

The extended and new analyses are shown in **Figure 3A** and explained in the RESULTS:

A lower expression of Cd36 in intestinal epithelial cells isolated from ileum of GF mice compared to conventional mice was reported previously [36]. In our study, no difference in Cd36 mRNA expression between GF, OMM^{12} and SPF mice in duodenum, ileum and jejunum tissue was found (**Figure 3A**). Palmitoylation of Cd36 mediated by palmitoylacyltransferases (Dhhc) 4 and 5 affects its membrane localization and activity [37, 38]. However, although the gut microbiota, such as Lactobacillus, can affect CD36 palmitoylation [39] (40767021), as well as the bile acid receptor TGR5 [40], for which the secondary bile acid deoxycholate is a agonist [41], similar DHCC4 and 5 expressions were detected in duodenum, jejunum and ileum between GF, OMM^{12} and SPF mice (**Figure 3A**). Interestingly, Fatp4 mRNA expression was elevated in duodenum and jejunum tissue of GF compared to OMM^{12} and SPF mice (**Figure 3A**), which could contribute to their higher absorption of FA 16:0[D5] and FA16:0[D31] in plasma and peripheral tissues (**Figure 1B-F**).
RESULTS, Lines 479-489

Also, we have analyzed the protein expression of CD36 in intestinal tissue samples of GF, OMM^{12} and SPF mice, but, unfortunately, it was quite challenging to find a satisfying and suitable antibody against mouse CD36 in small intestinal tissues. Of four tested commercially available "validated" antibodies, only one (PA1-16813 Invitrogen) allowed a detection of signal/band for CD36 at the expected size, but still with a relatively poor overall performance. CD36 consistently appeared as broad and smeared bands (probably due to its extensive glycosylation). Below a representative Western Blot for ileum tissue of GF, OMM^{12} and SPF mice is shown. A quantification of CD36 signals (relative to β-actin as housekeeper) revealed a quite large variation of protein abundances between all animals analyzed, but no significant differences between GF, OMM^{12} and SPF mice in duodenum, jejunum and ileum tissue (see Figure below). This result would be in agreement with our mRNA data. However, we chose not to include the western blot analyses in the paper due to the outlined experimental challenges. Further, we

think that the mRNA data are convincing enough. They show that CD36 cannot be related with the elevated intestinal absorption in GF mice, as do the findings reported in reference PMID: 28860383.

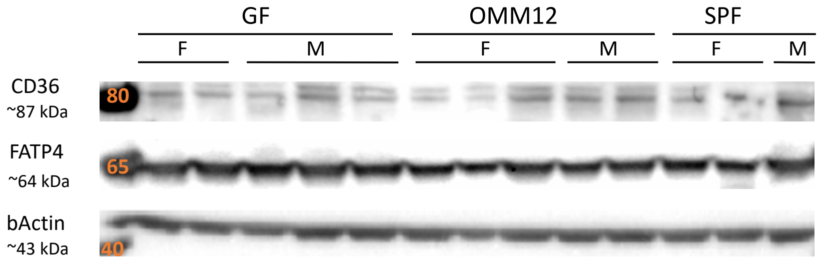

Representative western blot for CD36 in ileum samples of GF, OMM12 and SPF mice (Antibody: PA1-16813 Invitrogen).

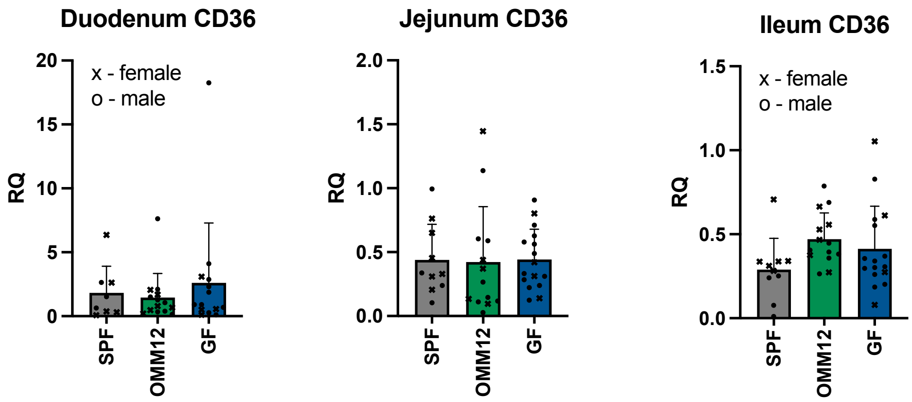

Quantification of CD36 bands in duodenum, jejunum and ileum tissue of GF, OMM^{12} and SPF mice (n = 9-16/tissue and group).

[4] The authors used physiology-based kinetic modelling to attribute the differences among the three tracers to "a single reduced absorption coefficient k_abs." Please corroborate this conclusion with independent experiments such as a lymph-fistula model or a dual-isotope intestinal-flux assay.

REPLY: As independent experiment to corroborate the K_{abs} reported in Figure 2, we have now added an experiment, in that GF, OMM^{12} and SPF mice were supplied with labelled FA for only 20 minutes aiming to determine absorption in duodenum tissue.
[Please let us also note, that according to the legal and regulatory requirements for animal experiments in Germany the suggested lymph-fistula model or a dual-isotope intestinal-flux assay are not doable in an adequate timeframe. These experiments would require a specific and new request of an approval for animal experiments by the local authorities, which takes at least 6-9 months in Germany before the experiments could just be started; as well as a practical experience in exactly these techniques, which we unfortunately do not have.]

The new data are shown in **Figure 2E** and commented in the RESULTS:

This is supported by the finding that 20 minutes after oral gavage, in duodenum tissue, 2.4-3.2 % of total FA 16:0 originated from absorption (FA16:0[D31] and FA 16:0[D5]), while in OMM^{12} and SPF mice only 1.3-1.6% and 1.0-1.4% (**Figure 2E**).
RESULTS, Lines 463-465

---

[5] Figure 3 only presents the relative composition 6 h post-gavage. Please provide absolute concentrations of lipids from GF, OMM12 and SPF mice, with statistical comparisons.

REPLY
As requested, we show now absolute concentrations (in addition to the lipid class composition presented as % of total) in **Figure 3E-F**.

---

[6] Tβ/ωMCA concentrations are negatively correlated with LPC levels, and the amounts of some unconjugated bile acids (e.g., CA) may also differ markedly among the mouse groups. How to determine which metabolite has the greatest impact on lipid absorption. Human bile is dominated by glycine-conjugated CA and CDCA. Could other bile acids modulate CEL hydrolytic activity?

REPLY: We agree and think that specifically the effect of TβMCA on PLA1 is of major relevance for our findings. Therefore, we tested it experimentally. We found that addition of TβMCA, whose levels are lower in GF compared to colonized mice, reduces PLA1 activity when added to GF bile. These data provide further and important evidence for BA-dependent regulation of PLA1 by the microbiome: Both, the elevated TCA and reduced TβMCA stimulate PLA1 activity in bile of SPF and OMM^{12} mice compared to GF animals.
Also, GCA and GCDCA was tested. While GCDCA had no effects, GCA increased PLA1 activity by app. 20% suggesting a potential relevance of our findings in humans.

The new data are shown in **Figure 6H-I** and commented in the RESULTS and DISCUSSION:

Of note, addition of TβMCA, whose levels are strongly lowered in bile of SPF and OMM^{12} mice (**Figure 6A**) reduced PLA1-mediated LPC18:1[D7] generation from PC15:0/18:1[D7] almost 2-fold (**Figure 6H**). Glyco-conjugated CA (GCA), a major human BA, increases PLA1 activity by 20%, while glyco-conjugated CDCA (GCDCA) had no effect (**Figure 6I**).
Together, these findings demonstrate, that in bile, PC to LPC conversion by PLA1 is stimulated by TCA and inhibited by TβMCA, whose levels depend on microbial colonization.

RESULTS, Lines 580-585

Human bile contains high amounts of glyco-conjugated 3α,7α-OH-containing CA (GCA) [61], which stimulates PLA1 activity, and comprises CEL implying a potential translational relevance of our findings. *RESULTS, Lines 680-681*

---

[7] An in vitro pure enzyme reaction demonstrating that CEL degrades PC to form LPC is required to confirm that CEL is the enzyme responsible for PC degradation in bile. Additionally, although the expression level of CEL remains unchanged in GF, OMM12, and SPF mice, how is its activity regulated? Further in vitro pure enzyme reactions should be supplemented to investigate whether TCA promotes the degradation of PC to LPC by CEL.

REPLY

While the manuscript was under review mice with a global CEL deficiency were bred. Investigation of PLA1 activity in bile after incubation with PC15:0/18:1[D7] for 2 and 15 min revealed a significant reduction when CEL was absent (Figure 5F). The LPC18:1[D7] levels were 50% lower in CEL-deficient compared to control mice. Accordingly, PC/LPC ratios were higher in CEL KOs than in controls. Please note, that it was also already previously shown that CEL hydrolyzes PC (PMID: 9148961). The reference was added to the discussion.

In addition, one hour after oral gavage with FA16:0[D5] and TG(16:0[D31])3, the concentrations of [D5]- and [D31]-labelled FA 16:0 were elevated by 60-60% in plasma and liver in SPF CEL KO mice compared to SPF controls (Figure 3H-I) demonstrating that CEL is involved in lipid absorption. The result that FA16:0[D5] and FA16:0[D31] levels were similar in GF and SPF CEL-knockout mice indicates that the gut microbiota limits systemic lipid uptake via CEL. Together, these data confirm that CEL is involved in systemic lipid uptake regulated by the gut microbiota.

The result, that CEL`s protein expression was found unchanged between GF, OMM[12], and SPF mice (Figure 5E) in bile led us to the idea that it could be regulated post-translationally. Because it was reported, that CEL is regulated by bile acids, particularly by TCA [CEL= previously known as bile salt-dependent lipase (BSDL)], as explained in the discussion (PMIDs: 12454261, PMID: 21081507, PMID: 9331420), we started to investigate the triad "Microbiota-BA-CEL/PLA1".

The new data are shown in **Figure 5F-I** and commented in the RESULTS and DISCUSSION:

To confirm an involvement of CEL in gut microbiota-dependent lipid absorption, mice with a global CEL deficiency were bred. Investigation of PLA1 activity in bile after incubation with PC15:0/18:1[D7] for 2 and 15 min revealed a significant reduction when CEL was absent (**Figure 5F**). The LPC18:1[D7] levels were 2.0-fold lower in CEL-deficient compared to control mice. Accordingly, PC/LPC ratios were 2.5-fold higher in CEL KOs than in controls (**Figure 5G**). Most importantly, one hour after oral gavage with

FA16:0[D5] and TG(16:0[D31])$_3$, the concentrations of FA16:0[D5] and FA16:0[D31] were 2.5 to 5.0-fold elevated in plasma and liver in SPF CEL KO mice compared to SPF controls demonstrating that CEL is involved in lipid absorption(**Figure 5H-I**). The result that FA16:0[D5] and FA16:0[D31] levels were not significantly different in GF CEL KO and SPF CEL KO mice indicates that the gut microbiota limits systemic lipid absorption via CEL.
RESULTS, Lines 551-560

CEL deficiency decreases PLA_1 activity in bile and increases systemic lipid uptake in SPF mice. *RESULTS, Lines 563-564*

In CEL-deficient mice housed in a SPF environment, PLA1 activity in bile was reduced and the lipid uptakes in plasma and liver increased, but similar to those determined in GF CEL KO mice demonstrating that CEL is involved in gut microbiota-dependent regulation of lipid absorption. *DISCUSSION, Lines 668-671*

---

[8] The authors could supplement the study with WT/MYD88-/- mice under different gut microbiota conditions (GF/SPF) to further elucidate the role of the MYD88-CYP7B1 signaling pathway in mediating the effects of gut microbiota on lipid absorption.

REPLY

To support the relevance of Myd88 signaling pathway for gut microbiota-dependent FA absorption, we have supplied Myd88-/- mice (housed in an SPF environment) with tracers for 1h, before the levels of labelled FA were quantified in plasma and liver tissue. We could detect decreased concentrations of FA16:0[D5] and FA16:0[D31] in plasma and liver of Myd88-/- mice compared to controls (Figure 8H), which is almost identical to the comparison GF vs. SPF.
To strengthen the evidence that Myd88 in involved in the regulation of Cyp7b1, Myd88 was knocked down in hepatocytes of the HepG2 cell line using RNAi (Figure 8I-J). These cell cultures experiments revealed a more than 3-fold higher mRNA expression of Cyp7b1 when cells were treated with siRNAs against Cyp7b1 than when treated with a non-targeting control siRNA.

The new data are shown in **Figure 8H-J** and commented in the RESULTS

In agreement, total LPC levels were 2.3-fold increased, but PC/LPC ratios 2.8-fold decreased in Myd88 KO animals as well as the systemic uptake of FA16:0[D5] and FA16:0[D31] one hour after oral gavage (**Figure 8F-H**) strengthening a relevance of Myd88 for lipid absorption. To confirm an involvement of Myd88 in regulation of Cyp7b1 expression, we finally knocked down Myd88 in the HepG2 hepatocyte cell line using RNAi (**Figure 8I-J**). These experiments revealed that in cells treated with siRNAs against Myd88, the mRNA expression of Cyp7b1 was 3.5-fold increased, which is in line with mRNA expression profiles detected in liver samples from Myd88 KO animals (**Figure 8B**).

RESULTS, Lines 633-640

MINOR COMMENTS

[1] Supplement the PC composition of bile in GF, OMM12, OMM11, and SPF mice. Is there a difference in the content of PC34:2 in the bile of these mice?

REPLY

As suggested, PC contents/composition in bile of GF, OMM^{11}, OMM^{12} and SPF were added as supplemental Figure. PC34:2 is by far the main PC species in all groups. Its contents are slightly higher in OMM^{11} and OMM^{12} than in GF and SPF mice.

The new data are shown in **Supplemental Figure 5** and commented in the RESULTS:

SPF mice were administered the tracer mix ±100nmol PC34:2 as major PC species in mouse bile, with slightly elevated contents in OMM^{11} and OMM^{12} compared to GF and SPF mice (**Figure S5**).
RESULTS, Lines 516-518

---

[2] Please list the exact species/strains comprising the OMM12 consortium in the Methods section.

REPLY: The OMM^{12} strains are shown in Figure 4A and were additionally added to the METHODS:

The strains of the OMM12 consortium are: *Turicimonas muris* (YL45), *Lactobacillus reuteri* (I49), *Bacteroides caecimuris* (I48), *Clostridium innocuum* (I46), *Blautia coccoides* (YL58), Muribaculum intestinale (YL27), *Enterocloster clostridioformis* (YL32), *Flavonifractor plautii* (YL31), *Akkermansia muciniphila* (YL44), *Acutalibacter muris* (KB18), *Bifidobacterium animalis* (YL2), *Enteroccocus faecalis* (KB1) [12].
METHODS, Lines 61-65

---

[3] Why is the total bile-acid concentration lower in OMM12 than in SPF and GF bile (Figure S6A)?

REPLY: Unfortunately, we do not have an explanation for these findings (now Figure S7). It seems that the mean values shown are driven by three outliers.

---

[4] "Lipid" may imply both fat and cholesterol. Please discuss whether the TCA—PC/LPC axis also affects intestinal cholesterol absorption.

REPLY: A potential relevance of gut microbiota-dependent PC degradation in bile for intestinal absorption of cholesterol was briefly discussed in the DISCUSSION:

We speculate that intestinal absorption of cholesterol, which was not investigated in this study, might be stimulated by gut microbiota-dependent PC degradation in bile, because luminal PC interferes with cholesterol absorption. For example, in human subjects, dietary supplementation with PC or an intraduodenally infusion a cholesterol-rich lipid emulsion containing PC resulted led to reduced cholesterol absorption [74-76].
DISCUSSION, Lines 723-727

---

[5] Have the authors used monocolonization experiments to verify the independent contribution of Akkermansia muciniphila to the TCA—PC/LPC axis and lipid absorption? Besides Akkermansia muciniphila, have the other bacteria in Oligo-MM12 been **reported** to affect lipid absorption?

REPLY

A relevance of *A. muciniphila* is certainly not the key finding of our paper plus its contribution to systemic lipid absorption might be only minor. However, we think that it might be still worth mentioning, because *A. muciniphila* was previously assumed to inhibit systemic lipid uptake and accumulation (PMID: 23671105, PMID: 31263284), which is per se consistent with our findings. Hence, we decided to tone down the claims related to *A. muciniphila*. (to: "*A.muciniphila* besides other microbial species contributes to intestinal lipid absorption") and add more evidence:

(i) The GF-OMM^{11}-OMM^{12} data were reanalyzed. We show now the absolute concentrations of FA16:0[D5] and FA16:0[D31] in Figure 4B-C (uniformly for all uptake experiments in the manuscript; before FA16:0[D5] and FA16:0[D31] were presented as % of total lipids for this experiment). In addition, the concentrations in liver and plasma were now tested for significance of difference between the groups of OMM^{11} and OMM^{12}. We found that the levels of FA taken up are significantly different between OMM^{11} and OMM^{12} in the liver ($p<0.05$), but not in plasma ($p=0.1$ and 0.07) (Figure 4B-C). The PC/LPC ratios in bile were found significantly different between OMM^{11} and OMM^{12} (Figure 4D).

(ii) Additional data from another experiment were added, where the uptake of FA16:0[D5] and FA16:0[D31] in plasma and liver of mice colonized with [Bacteroides thetaiotaomicron, Escherichia coli, and Eubacterium rectale] +/- *A.muciniphila* prior to oral gavage with labeled lipids for 1h was quantified. In agreement, with the OMM^{11}-OMM^{12} data, the levels of the labelled FA were lower when *A. muciniphila* was added to the EAM consortium. (Unfortunately, bile samples for measurements of PC/LPC ratios were not available from this experiment due to challenges/issues in bile collection from gallbladder.)

The reanalyzed and new data are shown in **Figure 4B-F** and commented in the RESULTS:

To support that *A. muciniphila* can affect systemic lipid absorption, the uptake of labelled lipids in plasma and liver of mice colonized with easy accessible microbiota (EAM) without or with *A.muciniphila* was tested. The EAM consortium consists of the three bacterial strains *Bacteroides thetaiotaomicron, Escherichia coli, and Eubacterium rectale*, representing the three most abundant bacterial phyla of human gut microbiota [13]. Addition of *A.muciniphila* to the EAM consortium decreased the amounts of FA16:0[D5] and FA16:0[D31] in plasma and liver one hour after oral gavage with tracers (**Figure 4E-F**) providing further evidence that *A.muciniphila* besides other microbial species contributes to lipid absorption.
RESULTS, Lines 505-512

To the best of our knowledge, no other bacteria of the OMM^{12} consortium were reported to affect TG or FA absorption.

---

[6] In addition to influencing bile acid synthesis pathways in the liver via MYD88, the gut microbiota itself can perform various modifications on bile acids such as TCA, thereby affecting the content and proportion of bile acids in bile. The authors should provide a more detailed elaboration on this aspect.

REPLY
Bacterial BSH cleaves the amid bond linking primary bile acids to taurine and glycine. Most relevant for this study are the of conversions TβMCA (=> βMCA) and TCA (=> CA). We have previously reported, that three strains of the OMM^{12} bacteria have a BSH activity (PMID: 33382950). BSH activity of the SPF microbiota was not yet tested, but we assume, that it could more BSH-positive bacteria due to a higher diversity. However, it`s difficult to directly compare the BSH activities between the OMM^{12} and SPF microbiota, since the few BSH-positive OMM^{12} strains, if dominant, could probably reach a similar biomass/cell numbers as the BSH-positive SPF strains. Therefore, BSH activity must not necessarily be different between OMM^{12} and SPF bacteria.

To determine BSH activity for deconjugation of TβMCA and TCA by the gut microbiota of OMM^{12} and SPF mice, caecal contents were incubated with TβMCA and TCA for 0, 30 and 90 min revealing similar βMCA and CA levels, deconjugation capacities, respectively. BSH activity for TβMCA and TCA in GF mice was identical as when incubated with PBS (negative control). Hence, we conclude that concomitant increase of the TCA to TβMCA ratio in GF>OMM12>SPF (Figure 7G) cannot be related with bacterial BSH.

The new data are shown in **Figure 7F** and commented in the RESULTS:

Bacterial bile salt hydrolyzes (BSH) cleaves the amide bond linking BA to taurine, deconjugating TβMCA to βMCA and TCA to CA, respectively. To test BSH activity in the gut microbiota of OMM^{12} and SPF mice, caecal contents were incubated with TβMCA and TCA for 30 and 90 min revealing similar βMCA and CA levels and thus, deconjugation capacities at both timepoints (**Figure 7F**). BSH activity for TβMCA and TCA in GF mice was identical as in the incubations with PBS (negative control). Hence, we conclude that concomitant increase of the TCA to TβMCA ratio in GF>OMM^{12}>SPF is not related to bacterial BSH. Instead, the major driver is the downregulation of Cyp7b1 associated with gut microbial colonization.

RESULTS, Lines 606-613

Point to point response to the referees (NMICROBIOL-25072607B)

Please find in the following our response to your comments.

All major changes are tracked in the manuscript and described in the following.

Reviewer 1

The authors have been responsive to the technical and some of the conceptual issues raised in the previous review. It is still not clear what signals from the gut microbiota are responsible for these effects and as such I think claims about new mechanistic insight are overstated. That said, I do think the study is important and likely of interest to readers. I would suggest as compromise that the authors temper their conclusions to more accurately reflect their findings.

Remaining issues:

1. The motility issue is still unclear. There are numerous papers where this has been quantified and appears to be independent of diet. I pasted a few below but note these and others are fairly consistent. It still remains possible that reduced motility is a factor in their results.

https://www.sciencedirect.com/science/article/pii/S0016508513001042?via=ihub
https://www.sciencedirect.com/science/article/pii/S2589004224021205?via=ihub

I recognize the authors may not have the capacity to redo these experiments with a dye to track motility along with their labeled lipid gavage. That would be ideal of course. However, I suggest that they carefully acknowledge and discuss their data in contrast to what others have shown.

REPLY:
The results of the two suggested papers were included in the DISCUSSION section:

Although others showed that microbial colonization of GF mice decreases whole gut transit times [39,40], here, the transit times of the gavaged lipid mix in the small intestine, which is most relevant for systemic FA uptake, were similar in GF, OMM[12] and SPF mice.
DISCUSSION, Lines 274-277

Reviewer 2

The authors did a great job in their additional experiments and corrections. The new data on CEL KO mice is especially valuable. I have only a few comments.

Line 484: I believe that (40767021) is a personal note about the reference, which should be removed from the text.

Figure 3A is not well organized, as we see the same genes in different columns. It could be different regions of the small intestine, which should be better labeled.

Typo in line 606: hydrolyzes should be hydrolase

Line 652-663: It is nice that the authors discuss other similar studies, but they do not compare them to their own study. It would be important to discuss why there seems to be some discrepancy between their data and the literature.

REPLY:
The typos were corrected and previous Figure 3A, which is now Extended Data Figure 3C, was modified. It was labelled with the different regions of the small intestine.

The results of the indicated studies and differences compared to our work were further described in the DISCUSSION:

Wang and colleagues reported that GF mice fed a high fat diet contained lower amounts of unsaturated FAs in their intestinal epithelial cells compared to those of conventional mice using a colorimetric assay, which does not detect saturated FA[36]. Application of lipoprotein lipase (LPL) inhibitor blocking peripheral lipid uptake prior to gavage of [3H] -labelled TG, revealed a higher accumulation of [3H] over 7h in plasma of SPF compared to GF on a chow and high fat diet [9]. Surprisingly, [3H]-TG uptake kinetics were largely similar in plasma of LPL inhibitor treated and untreated mice, in which, after a first peak, a decrease of systemic lipids would be expected due to flux in peripheral tissues, as observed here. *DISCUSSION, Lines 265-272*

Reviewer 3

They have addressed all of my concerns. It's OK for me now.
